# A Long Non-coding RNA Signature to Improve Prognostic Prediction of Pancreatic Ductal Adenocarcinoma

**DOI:** 10.3389/fonc.2019.01160

**Published:** 2019-11-08

**Authors:** Chenhao Zhou, Shun Wang, Qiang Zhou, Jin Zhao, Xianghou Xia, Wanyong Chen, Yan Zheng, Min Xue, Feng Yang, Deliang Fu, Yirui Yin, Manar Atyah, Lunxiu Qin, Yue Zhao, Christiane Bruns, Huliang Jia, Ning Ren, Qiongzhu Dong

**Affiliations:** ^1^Department of Liver Surgery, Liver Cancer Institute, Zhongshan Hospital, Fudan University, Shanghai, China; ^2^Department of General Surgery, Huashan Hospital & Cancer Metastasis Institute, Fudan University, Shanghai, China; ^3^Department of Breast Surgery, Zhejiang Cancer Hospital, Zhejiang, China; ^4^Institutes of Biomedical Sciences, Fudan University, Shanghai, China; ^5^Department of Pancreatic Surgery, Pancreatic Disease Institute, Huashan Hospital, Fudan University, Shanghai, China; ^6^Department of General, Visceral and Cancer Surgery, University Hospital of Cologne, Cologne, Germany; ^7^Institute of Fudan Minhang Academic Health System, Minhang Hospital, Fudan University, Shanghai, China

**Keywords:** pancreatic ductal adenocarcinoma, lncRNA, signature, prognosis, overall survival

## Abstract

**Background:** Pancreatic ductal adenocarcinoma (PDAC) remains one of the most aggressive solid malignant tumors worldwide. Increasing investigations demonstrate that long non-coding RNAs (lncRNAs) expression is abnormally dysregulated in cancers. It is crucial to identify and predict the prognosis of patients for the selection of further therapeutic treatment.

**Methods:** PDAC lncRNAs abundance profiles were used to establish a signature that could better predict the prognosis of PDAC patients. The least absolute shrinkage and selection operator (LASSO) Cox regression model was applied to establish a multi-lncRNA signature in the TCGA training cohort (*N* = 107). The signature was then validated in a TCGA validation cohort (*N* = 70) and another independent Fudan cohort (*N* = 46).

**Results:** A five-lncRNA signature was constructed and it was significantly related to the overall survival (OS), either in the training or validation cohorts. Through the subgroup and Cox regression analyses, the signature was proven to be independent of other clinic-pathologic parameters. Receiver operating characteristic curve (ROC) analysis also indicated that our signature had a better predictive capacity of PDAC prognosis. Furthermore, ClueGO and CluePedia analyses showed that a number of cancer-related and drug response pathways were enriched in high risk groups.

**Conclusions:** Identifying the five-lncRNA signature (RP11-159F24.5, RP11-744N12.2, RP11-388M20.1, RP11-356C4.5, CTC-459F4.9) may provide insight into personalized prognosis prediction and new therapies for PDAC patients.

## Introduction

As reported in Cancer Statistics (2019), pancreatic cancer (PC), one of the most aggressive solid malignant tumors, is the fourth cause of cancer related deaths in the USA and it is estimated that 56,770 PC cases occurred and 45,750 patients died from PC in 2019. Additionally, PC incidence rate and death rate continues to increase, and a 5-year relative survival rate for it is the lowest (9%) compared with other cancers (1). Furthermore, pancreatic ductal adenocarcinoma (PDAC) accounts for the most part of PC (2). Due to atypical symptoms of PDAC and ineffective diagnosis methods, the majority of PDAC patients were diagnosed at an advanced stage, leading to patients not having the chance to be resected (1–4). Given current medical capabilities, surgical resection for resectable PDAC patients offers the only chance of a cure, but the survival of patients has not improved and commonly recurs within 12 months (5, 6). As for PDAC patients being diagnosed at an advanced stage, chemotherapy such as nab-paclitaxel plus gemcitabine and other methods including radiotherapy, molecular targeted therapy (MTT), and immunotherapy have only yielded modest improvements in survival (7, 8). Therefore, further exploration concerning the molecular mechanism underling PDAC occurrence and progression are essential to improve the early diagnostic biomarkers and therapeutic targets.

Long non-coding RNAs (lncRNAs) are defined as transcripts with a length of over 200 nucleotides, and lack protein-coding potential (9, 10). Studies have demonstrated that lncRNAs play important roles in a variety of biological processes, including epigenetic regulation, alternative splicing, imprinting, cell cycle control, cell differentiation, drug resistance, and tumorigenesis (11–14). Furthermore, emerging investigations have indicated that lncRNAs expression is abnormally dysregulated in numerous cancers and many lncRNAs are correlated with cancer recurrence, metastasis, and poor prognosis (15–17). For instance, the dysregulation of lncRNAs, including Linc01060, AGAP2-AS1, LINC00958, participate in the incidence, metastasis and progression of PDAC (18–20). Carbohydrate antigen 19-9 (CA19-9) is currently confirmed as the prognostic biomarkers for PDAC, but its low specificity urges us to discover and identify more potential and valuable molecular biomarkers for patients with PDAC (21). Increasing evidence suggests that lncRNA could serve as a good choice in predicting the prognosis of cancer (22–24). Meanwhile, a lot of gene expression signatures were successfully established to predict the clinical outcome of many different types of cancer (25, 26).

There are many lncRNAs which have reportedly been associated with PDAC prognosis (27, 28). However, they are seldomly used in clinical practice considering that their signature has not been validated in an independent cohort, or they do not adopt the appropriate statistical approach to generate the model. Here, we aim to identify the potential minimum number of robust lncRNAs as a signature to predict the prognosis of PDAC patients. Therefore, we mined the lncRNA data from The Cancer Genome Atlas (TCGA) database using the least absolute shrinkage and selection operator method (LASSO) algorithm, which can effectively analyze the high-dimensional sequencing data (29). We then validated this 5-lncRNA signature in our own PDAC patients from Fudan University using qRT-PCR technology and evaluated the accuracy of this signature and predictive capacity in the entire TCGA cohort.

## Methods

### PDAC Datasets Preparation

The level three RNA sequencing data and relevant clinical information of 177 PDAC patients were downloaded from the TCGA database (http://cancergenome.nih.gov/) and enrolled in our study. Additionally, 46 fresh frozen primary PDAC samples from the Fudan validation cohort were collected consecutively at Huashan Hospital from October 2010 to February 2014. All enrolled patients met the inclusion and exclusion criteria as follows: (1) pathologic diagnosis of PDAC without other types of pancreatic cancer; (2) no cases with other malignant cancers. Conventional clinicopathologic variables like age, gender, AJCC Tumor Node Metastasis (TNM) stage, histologic grade, and microsatellite instability (MSI) status were analyzed in our study. Written informed consent was obtained from all patients. The study was conducted in accordance with the Declaration of Helsinki, and the Ethical Committee of Huashan Hospital, Fudan University approved the study.

### RNA-seq Data Processing and lncRNA Profile Mining

To obtain the lncRNA expression profile, we mapped the probes of the TCGA RNA-seq data to lncRNA annotation files in the GENCODE database (http://www.gencodegenes.org). The annotation data (antisense, lincRNA, and sense_intronic) of probes was recognized as lncRNA. Fifteen thousand eight hundred and ninety-nine lncRNA probes were acquired in the RNA-seq data of PDAC. After removing lncRNAs whose expression was zero in more than 20% of the samples, 6,010 annotated lncRNA probes were generated for further study.

### RNA Extraction and Quantitative Reverse Transcription PCR (qRT-PCR)

TRIzol reagent (Invitrogen, USA) was used to extract total RNA from 46 PDAC samples (Fudan validation cohort) following the manufacture's protocol. Reverse-transcription was then carried out using a PrimeScript RT reagent kit (Takara, Japan). An ABI Prism 7500 Sequence Detection System (Applied Biosystems, Foster City, CA, USA) was used to carry out the qRT-PCR using SYBR® Premix ExTaq™ (Takara, Japan). ACTB (β-actin) was utilized as an internal control to normalize the expression of lncRNAs. The –ΔCT method (ΔCT = CT lncRNA – CT ACTB RNA) was applied to calculate each lncRNA expression level. The primers of related lncRNAs are shown in Table S1.

### Identification and Validation of the Prognostic lncRNA Signature

First, all the TCGA PDAC patients were randomly divided into two cohorts: the first 107 patients (60%) were termed the training cohort, and the remaining 70 (40%) the validation cohort. A univariate Cox regression model was applied to the training cohort to detect the prognostic lncRNAs. A set of lncRNAs whose *P*-value was < 0.05 were identified. These lncRNAs were then analyzed in a training cohort utilizing R software (version 3.6.0) and the “glmnet” package (R Foundation for Statistical Computing, Vienna, Austria) to carry out the LASSO Cox regression model analysis. Ten-times cross-validations were used to find the best penalty parameter lambda (29, 30). A list of prognostic lncRNAs with related coefficients were obtained from the lncRNA expression profile and the patient's overall survival (OS) according to the best lambda value. Furthermore, the risk score of every patient was calculated according to the expression level of each prognostic lncRNA and its corresponding coefficient. PDAC patients were assigned to a high-risk or low-risk group using the median risk score as the cut-off point. In the end, the OS differences between the two groups were evaluated by the Kaplan–Meier survival curves. Meanwhile, the prognostic lncRNA signature was validated in the TCGA and Fudan validation cohort. Based on the median risk score, the validation cohort was also split into a high-risk or low-risk group, and OS differences were analyzed as described earlier.

### Statistical Analysis

All statistical analyses were performed using R software (version 3.6.0) and Bioconductor (31). For further analysis, the lncRNA expression profile was log2-transformed. Univariable, multivariable Cox regression, and stratified analyses were performed to evaluate if the lncRNA signature was independent of age, gender, TNM stage, grade, and MSI status. Student's *t* or the Fisher's exact test was used to assess the relationship between lncRNA signature and other clinicopathologic variables. The method of Kaplan–Meier and the log-rank test were utilized to evaluate the OS differences between the high or low-risk groups. Receiver operating characteristic (ROC) analysis was further used to assess the prognostic value based on the multi-lncRNA risk score, histologic grade, TNM stage, and the combined model of risk score and other indexes. “pROC” package was applied to the ROC curve analysis, and “delong” methodology was adopted to study significant differences between the ROC curves. When a two-sided *P*-value was < 0.05, the statistical analyses were defined as statistically significant.

### Functional Enrichment Analysis

Differentially expressed genes (DEGs) between the high and low-risk PDAC patients in the TCGA dataset were identified using the classical *t*-test. The top 1,000 up-regulated and down-regulated DEGs were included in the functional enrichment analysis (Tables S2, S3). The Database for Annotation, Visualization and Integrated Discovery (DAVID, https://david.ncifcrf.gov/) and Cytoscape plug-in ClueGO and CluePedia were used to perform the enrichment analysis (32–34). The threshold for the analyses was set as a *P* < 0.05. By using Cytoscape software, the significant functional enrichment results were visualized in our study.

## Results

### Clinical Characteristics of PDAC Patients

**Figure 1:**
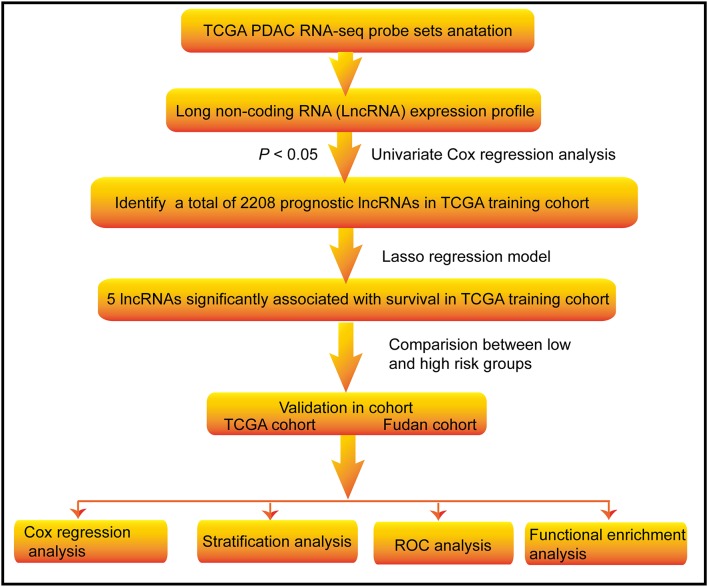
Flow chart of the study. The study was carried out in TCGA and Fudan lncRNA dataset of PDAC patients. The TCGA training cohort was used to identify prognostic lncRNAs. The LASSO regression model was used to establish a prognostic signature based on the prognostic lncRNAs. The prognosis analysis was validated in the TCGA and Fudan validation cohort, respectively.

The flowchart of this study is shown in Figure 1. A total of 223 PDAC patients were enrolled in our study. The detailed clinical characteristics of these patients are summarized in Table 1. The 177 TCGA PDAC patients were randomly assigned to a TCGA training cohort (*N* = 107) and TCGA validation cohort (*N* = 70). Additionally, 46 PDAC patients from Huashan Hospital, Fudan University were recruited as another independent validation cohort. As shown in Table 1, the vast majority of patients (65.47%) were aged over 60 years, and about 54.71% of PDAC patients were male. One-hundred-and-forty TCGA patients (79.10%) has a microsatellite stable (MSS) status. Moreover, most tumors were diagnosed in well-differentiated groups (histologic grade 1&2; 71.43%) and in early stage groups (TNM I&II; 90.37%).

**Table 1:** Clinical characteristics of 223 pancreatic adenocarcinoma patients involved in the study.

**Characteristics**	**All (*N* = 223)**	**Detailed data**		
		**Training cohort (*N* = 107)**	**Validation cohort (*N* = 70)**	**Fudan cohort (*N* = 46)**
Age at diagnosis, years				
≤ 60	77 (34.53%)	35 (15.70%)	19 (8.52%)	23 (10.31%)
≥60	146 (65.47%)	72 (32.29%)	51 (22.87%)	23 (10.31%)
Gender				
Female	101 (45.29%)	50 (22.42%)	30 (13.45%)	21 (9.42%)
Male	122 (54.71%)	57 (25.56%)	40 (17.94%)	25 (11.21%)
MSI status				
MSI-I	28 (15.82%)	14 (7.91%)	14 (7.91%)	–
MSI-L	9 (5.08%)	2 (1.13%)	7 (3.95%)	–
MSS	140 (79.10%)	91 (51.41%)	49 (27.68%)	–
Histologic grade				
G1	30 (17.14%)	22 (12.57%)	8 (4.57%)	–
G2	95 (54.29%)	55 (31.43%)	40 (22.86%)	–
G3	48 (27.43%)	28 (16%)	20 (11.43%)	–
G4	2 (1.14%)	1 (0.57%)	1 (0.57%)	–
TNM stage				
I	31 (14.22%)	12 (5.50%)	9 (4.13%)	10 (4.59%)
II	166 (76.15%)	88 (40.37%)	57 (26.15%)	21 (9.63%)
III	15 (6.88%)	2 (0.92%)	2 (0.92%)	11 (5.05%)
IV	6 (2.75%)	4 (1.83%)	1 (0.46%)	1 (0.46%)

*MSI, Microsatellite instability; MSS, Microsatellite stable; MSI-I, MSI-indeterminate; MSI-L, MSI-low; TNM, Tumor node metastasis*.

### Generate Prognostic lncRNAs From TCGA Training Cohort

Using the univariate Cox regression analysis method, a set of 2,208 prognostic lncRNAs were identified in the TCGA training cohort (*P* < 0.05). A LASSO Cox regression model was further applied to those 2,208 lncRNAs to generate a prognostic signature in the training cohort. As a result, we recognized the five-lncRNA signature that was highly associated with OS in PDAC patients. A list of lncRNAs with their acquired coefficients, gene symbol, ensembel ID, gene type, *P*-values, and hazard ratio are demonstrated in Table 2. Among those lncRNAs, lower lncRNA expression levels were revealed by negative coefficients. Interestingly, the five lncRNAs identified, had completely negative coefficients - RP11-159F24.5, RP11-744N12.2, RP11-388M20.1, RP11-356C4.5, and CTC-459F4.9, which meant they were correlated with better survival.

**Table 2:** lncRNAs significantly associated with the overall survival.

**Gene symbol**	**Ensembel ID**	**Coefficient**	**Gene type**	***P*-value**	**Hazard ratio**
RP11-159F24.5	ENSG00000248240.1	−0.00507	Antisense	0.001618	0.608225
RP11-744N12.2	ENSG00000254703.2	−0.01977	Antisense	0.03337	0.756667
RP11-388M20.1	ENSG00000260060.1	−0.00315	Antisense	0.000288	0.518596
RP11-356C4.5	ENSG00000261172.1	−0.04621	LincRNA	0.013033	0.724935
CTC-459F4.9	ENSG00000281468.1	−0.03738	Sense_intronic	3.08E-05	0.568016

### The 5-lncRNA Signature and the Patients' Survival in the Training Cohort

**Figure 2:**
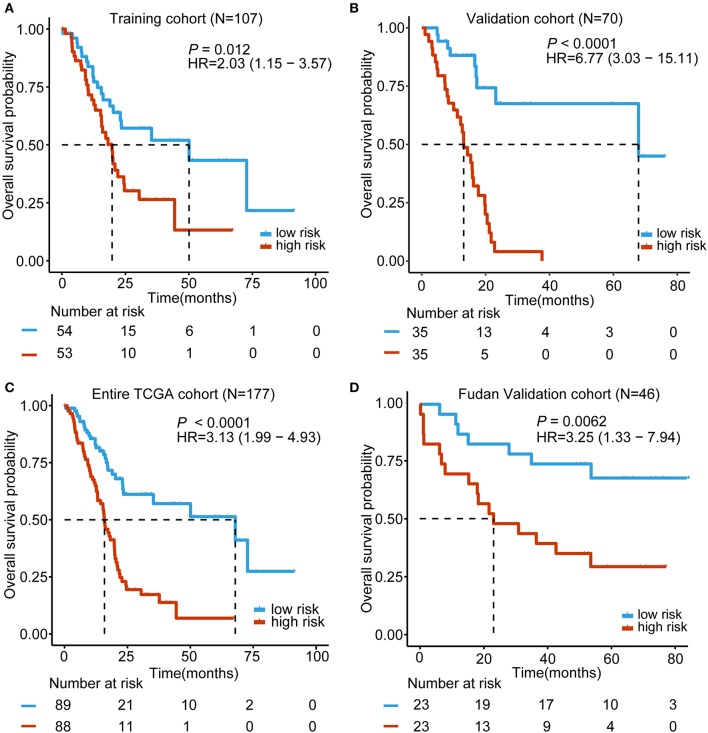
Kaplan–Meier analyses of the overall survival (OS) based on the 5-lncRNA signature. **(A)** TCGA training cohort (*N* = 107); **(B)** TCGA validation cohort (*N* = 70); **(C)** Entire TCGA cohort (combined training and validation patients, *N* = 177); **(D)** Fudan validation cohort (*N* = 46). The tick marks on the Kaplan–Meier curves represent the censored subjects. The differences between the two curves were determined by the two-side log-rank test. The number of patients at risk is listed below the survival curves.

Based on the expression of these five lncRNAs for OS prediction, we established a risk-score formula: Risk score = (−0.00507^*^expression level of RP11-159F24.5) + (−0.019766164^*^ expression level of RP11-744N12.2) + (−0.003146176^*^ expression level of RP11-388M20.1) + (−0.046208838^*^ expression level of RP11-356C4.5) + (−0.037384417^*^ expression level of CTC-459F4.9). Furthermore, we worked out the 5-lncRNA signature risk score for every patient in the TCGA training cohort. Using the median risk score as the cut-off point, the patients were categorized into a low risk group (*N* = 54) and high-risk group (*N* = 53). The Kaplan–Meier survival analysis indicated that patients in the high-risk group were associated with a tendency toward worse outcomes in the training cohort (HR = 2.03, 95% CI = 1.15–3.57, *P* = 0.012) (Figure 2A).

### Validation of the 5-lncRNA Signature for Survival Prediction in the Validation Cohorts and Entire TCGA Cohort

In order to confirm the power of the 5-lncRNA signature in predicting the OS of PDAC patients, we validated our results in the internal validation cohort. By utilizing the same classification method, patients were classified into a high-risk group (*N* = 35) and a low risk group (*N* = 35). Consistent with previous findings, patients in the high-risk group revealed significantly worse OS compared to the other patients (HR = 6.77, 95% CI = 3.03–15.11, *P* < 0.0001) (Figure 2B). Moreover, in the entire TCGA cohort, which included the training and validation cohort, the 5-lncRNA signature also had similar results (HR = 3.13, 95% CI = 1.99–4.93, *P* < 0.0001) (Figure 2C). What is more, we validated the risk score-based classification in the independent cohort from Huashan hospital, Fudan university. The Fudan validation cohort verified the capacity of the signature in predicting OS. As shown in Figure 2D, our signature can also effectively discriminate the risk of OS (HR = 3.25, 95% CI = 1.33–7.94, *P* = 0.0062).

### Association Between 5-lncRNA Signature and Clinic-Pathologic Characteristics in PDAC Patients

The patients of the TCGA cohort and the Fudan validation cohort were divided into two groups for the purpose of investigating the importance of the 5-lncRNA signature in PDAC clinic-pathologic sides. As depicted in Table S4, a high-risk score of the signature was associated with aggressive tumor clinic-pathologic parameters, including TNM stage (*P* = 0.024, TCGA cohort) and OS status (*P* < 0.001, TCGA cohort; *P* = 0.008, Fudan validation cohort). Although there was no statistical significance of the histologic grade in the TCGA cohort and TNM stage in the Fudan validation cohort, it still had the trend that patients in the high-risk group showed a poor differentiated grade and an advanced tumor stage.

### Investigate the 5-lncRNA Signature Prognostic Capacities by Univariate and Multivariate Analyses

**Figure 3:**
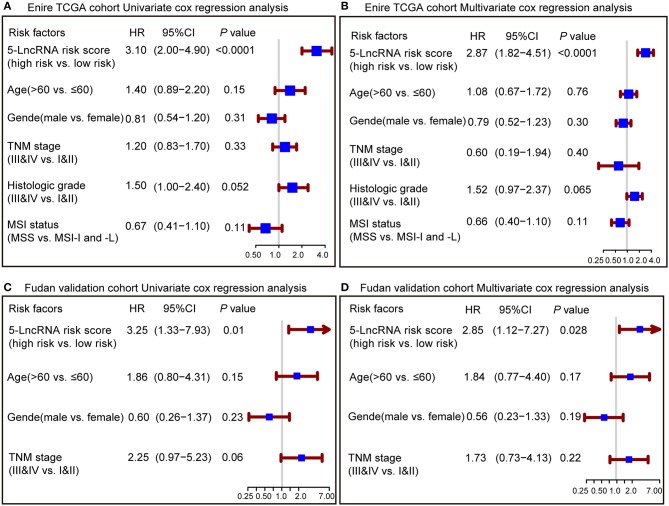
Forest plot summary of analyses of overall survival (OS). Univariate and multivariate analyses based on the 5-lncRNA signature and clinical covariates in the entire TCGA cohort **(A,B)** and Fudan validation cohort **(C,D)**. The blue solid squares represent the hazard ratio (HR), and the red transverse lines represent 95% confidence intervals (CI). All *P*-values were calculated using Cox regression hazards analysis.

To confirm whether the prognostic capacity of our 5-lncRNA signature was independent from the clinic-pathologic characteristics, univariate and multivariate Cox analyses were carried out by analyzing the available co-variables like 5-lncRNA risk score, age, gender, TNM stage, histologic grade, and MSI status in the entire TCGA cohort and Fudan validation cohort. In the univariate Cox regression analyses, the 5-lncRNA signature was a powerful variable associated with prognosis in both the entire TCGA and Fudan validation cohort (HR = 3.10, 95% CI = 2.00–4.90, *P* < 0.0001; HR = 3.25, 95% CI = 1.33–7.93, *P* = 0.01, respectively) (Figures 3A,C). By using other clinical variables to adjust the multivariate analyses, the 5-lncRNA signature still proved to be a strong and independent variable in the above described cohorts (HR = 2.87, 95% CI = 1.82–4.51, *P* < 0.0001; HR = 2.85, 95% CI = 1.12–7.27, *P* = 0.028, respectively) (Figures 3B,D).

### Subgroup Analyses Based on the 5-lncRNA Signature According to TNM Stage, Histologic Grade, and MSI Status

**Figure 4:**
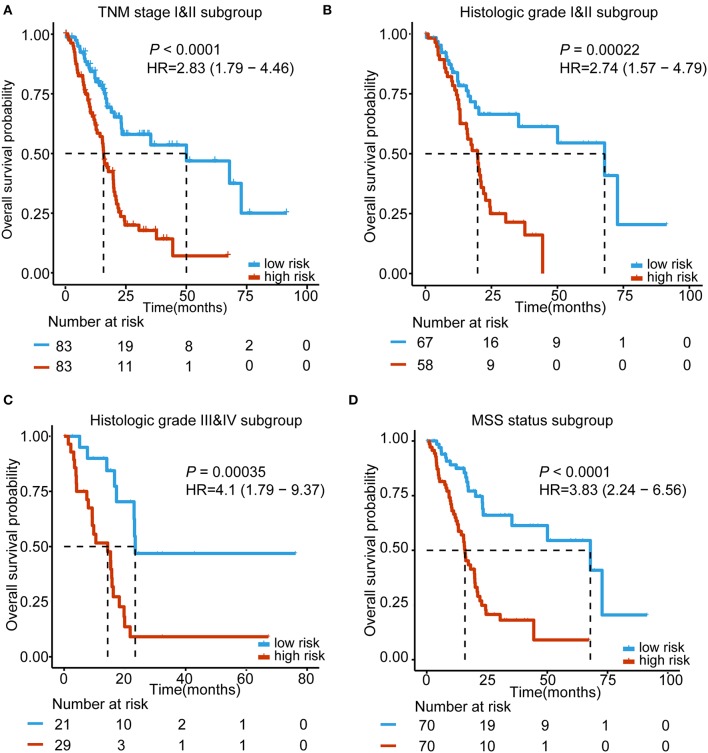
Kaplan–Meier survival analysis to assess the independence of the 5-lncRNA signature from the TNM stage, histological grade, and MSS status. The patients from the entire TCGA were stratified into subgroups. The 5-lncRNA signature was applied to the TNM stage II and III patients **(A)**, histological grade I&II patients **(B)**, histological grade III&IV patients **(C)**, MSS status patients **(D)**, separately. The number of patients at risk is listed below the survival curves. The tick marks on the Kaplan–Meier curves represents the censored subjects. Two-sided log-rank test was adopted to determine the differences between the two curves.

For the purpose of testing whether our 5-lncRNA signature can play a role in different TNM stages, histologic grades and MSI status, subgroup analyses were carried out, respectively. Because the number of TNM stage III&IV and not MSS status patients was small, we performed our subgroup analysis in the early stage (TNM stage I&II) and MSS status of TCGA PDAC patients. Figure 4A shows that our 5-lncRNA signature could successfully predict the survival outcome in this subgroup (HR = 2.83, 95% CI = 1.79–4.46, *P* < 0.0001). Furthermore, TCGA PDAC patients were stratified into a well-differentiated group (histologic grade I&II) and poor-differentiated group (histologic grade III&IV). The subgroup analyses demonstrated that the 5-lncRNA signature could be beneficial to divide patients into low risk and high risk groups in every grade with a statistically significant difference for the well-differentiated group (HR = 2.74, 95% CI = 1.57–4.79, *P* = 0.00022; Figure 4B) and poor-differentiated group (HR = 4.1, 95% CI = 1.79–9.37, *P* = 0.00035; Figure 4C). Figure 4D indicates that patients with an MSS status in the high-risk group had importantly shorter median OS than the low risk patients (HR = 3.83, 95% CI = 2.24–6.56, *P* < 0.0001). These results revealed that the prognostic ability of the 5-lncRNA signature was independent of the TNM stage, histologic grade and MSI status.

### Subgroup Analyses Based on the 5-lncRNA Signature According to Age and Gender

As we know, age is an important risk factor in the progress of carcinogenesis (35). We performed the subgroup analyses by separating these patients into ≤ 60-year-old and ≥60-year-old subgroups. Regardless of patients in the younger or older groups, our 5-lncRNA signature still had the capacity to identify patients with different prognoses (Figures S1A,B). We also studied whether our 5-lncRNA signature was independent of gender by adopting similar methods as described above. In the entire TCGA PDAC cohort, the high-risk score of our signature significantly associated with an advert OS either in female or male patients (*P* = 0.0039, *P* < 0.0001, respectively; Figures S1C,D). These results further demonstrates that our 5-lncRNA signature was definitely independent of age and gender.

### ROC Analysis to Assess the Prognostic Value of the 5-lncRNA Signature

**Figure 5:**
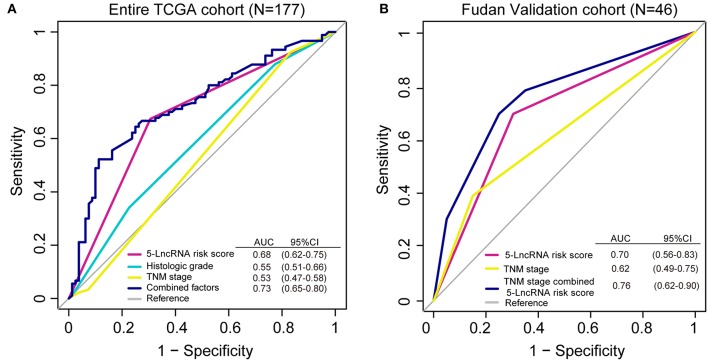
Receiver operating characteristic (ROC) analysis of the sensitivity and specificity of the overall survival (OS) prediction by the 5-lncRNA risk score, histologic grade, TNM stage and all combined risk factors in the entire TCGA cohort (**A**; *N* = 177) and the Fudan validation cohort (**B**; *N* = 46). As shown, the 5-lncRNA risk score combined with other factors shows a better prediction of OS either in the TCGA cohort or Fudan validation cohort.

We conducted a ROC analysis to assess whether our 5-lncRNA signature could behave better than the present clinical parameters in predicting OS prognosis. As Figure 5A shows, the area under receiving operating curve (AUROC) of the 5-lncRNA risk score model was superior to those of TNM stage and histologic grade (0.68 vs. 0.53; 0.68 vs. 0.55). In addition, the AUROC of 5-lncRNA risk score combined with TNM stage and histologic grade showed significant differences when compared to the signature alone (0.73 vs. 0.68). This phenomenon was also confirmed in our Fudan validation cohort (Figure 5B). Because there was no data related to the histologic grade of our patients, ROC analysis was performed according to the 5-lncRNA risk score and TNM stage. The results were similar to those in the entire TCGA cohort. The 5-lncRNA risk score possessed a better performance than TNM stage (0.70 vs. 0.62). What is more, when combined with our signature TNM stage, the combined model had a strong power for OS prediction (AUROC = 0.76).

### Enriched Functions and Pathways Associated With 5-lncRNA Signature

**Figure 6:**
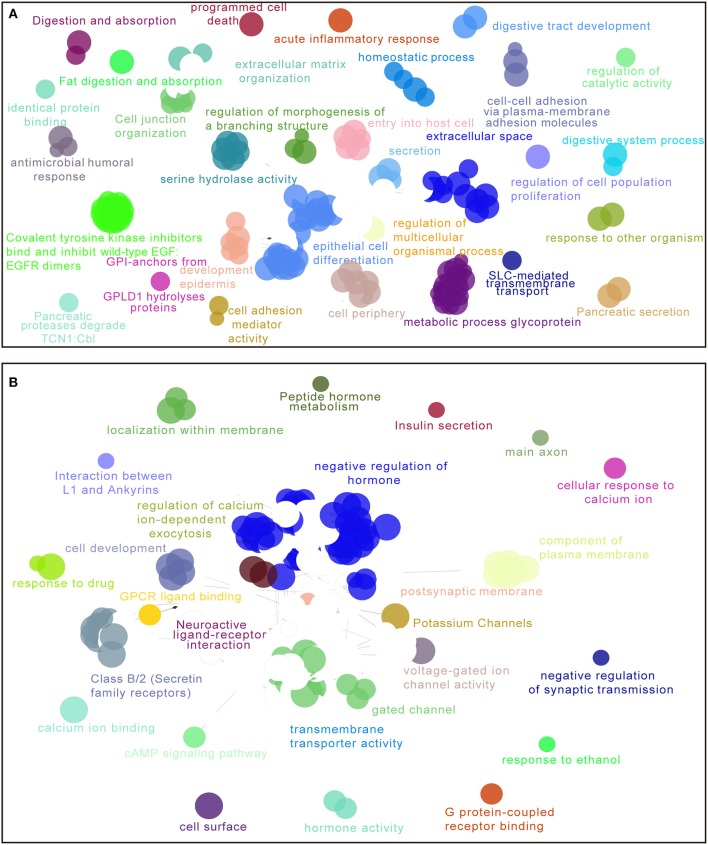
Enriched functions and pathways of the top 1,000 significantly differentially expressed genes (DEGs) in high vs. low risk PDAC patients in the TCGA dataset. The interaction network was generated with the Cytoscape plug-in ClueGO and CluePedia. Functions and pathways of up-regulated DEGs **(A)**, down-regulated DEGs **(B)**. The size of the nodes shows the term significance after Bonferroni correction. The significant term of each group is highlighted.

For the purpose of elucidating the 5-lncRNA related biological functions and pathways, we used cytoscape plug-in ClueGO and CluePedia to analyze the entire TCGA PDAC cohort according to the classification through our signature. Figure 6 shows a set of enriched functions and pathways of the top 1,000 significantly DEGs in high vs. low risk PDAC patients in the TCGA dataset. In the analyses of the top 1,000 up-regulated DEGs in high risk groups, we found that a group of cancer-related pathways like programmed cell death, regulation of cell population proliferation, epithelial cell differentiation, cell adhesion, and the secretion of the pancreas were involved (Figure 6A). Similarly, several drug response pathways, signal transmission pathways including gated channel, negative regulation of synaptic transmission, and the cAMP signaling pathway, as well as the secretin receptors related to the pancreas were also found to be changed in the pathways of the top 1,000 down-regulated DEGs in high risk groups (Figure 6B). Based on the above analyses, we propose that our 5-lncRNA signature could play a significant role in the carcinogenesis of PDAC.

## Discussion

At present, with the emergence of many lncRNAs, researchers have altered their study attention from the traditional protein-coding genes to non-coding RNAs. Many publications nowadays have revealed that lncRNA plays an important role in the process of tumor development. Zhang et al. and Song et al. have reported, respectively, that they found the multi-lncRNA signatures that can predict the prognosis of pancreatic cancer. However, several limitations exist in their studies. For example, their signatures have not been validated in an independent validation cohort (27, 28). In addition, Zhang et al. only used the Cox proportional hazards regression method to generate the model. This method was reported to be inappropriate for high-dimensional sequencing data when the ratio between the parameters and sample size is too low (36).

In our study, we constructed the PDAC lncRNA prognosis signature by mining the lncRNA data from the TCGA database using a LASSO algorithm. By investigating the relationship between the clinical data and lncRNA expression profiles of PDAC patients in TCGA training cohort, we established a novel 5-lncRNA signature that was significantly related to the OS. By utilizing this signature to the patients in the TCGA PDAC training cohort, statistical significance was seen in the survival curve between the high and low risk groups. The prognostic capacity of this 5-lncRNA signature could be internally verified in the TCGA PDAC validation cohort and our independent Fudan validation cohort, revealing the wide application and efficacy of this signature in predicting PDAC prognosis.

Moreover, we performed univariate and multivariate analyses to verify that our 5-lncRNA signature can be an independent risk variable in predicting PDAC OS. Although those clinical parameters like age, gender, histologic grade, and TNM stage did not have statistical significance in the TCGA cohort and Fudan validation cohort by multivariate analyses, we applied the subgroup analyses to further confirm the independence of our 5-lncRNA signature. As we know, it is widely agreed that TNM stage can work as an influential factor in predicting the prognosis of PDAC. TNM stage I&II generally indicates the early stage of PDAC, while TNM stage III&IV indicates the late stage. We classified all the TCGA PDAC patients into early stage and late stage stratums to promote further analysis. The stratified patients were successfully separated into high and low risk subgroups based on the 5-lncRNA signature, and there was an obvious split in the overall survival curve of TNM I&II between them. The number of TNM III&IV patients was little, so we did not further analyze this subgroup. Additionally, tumor histologic grade has also been reported to be an effective prognostic factor in PDAC (37, 38). Our 5-lncRNA signature could also successfully discriminate the high or low risk of patients in both the well and poor differentiated subgroup. MSI status was reported to be a significant predictor for resectable pancreatic cancer patients, and the prognosis of MSI-H status patients was better than MSS status patients (39). Given the terrible survival of MSS status patients and because the number of MSI-indeterminate (MSI-I)/MSI-low (MSI-L) patients was not enough for analysis, we tested the independence of our signature in the MSS status group. Patients of MSS status in the high-risk group were found to have shorter median OS than low risk patients.

In addition, there are many theories on aging. Some researchers think that the accumulation of genomic and epigenomic instability promotes cancer, while some think that tissue overstimulation and the hyper-activation of the DNA damage response causes cancer (40). PDAC was reported to be an age-dependent cancer (35). Our 5-lncRNA risk model maintained its powerful prognostic capacity when stratified by age distribution. Furthermore, there are reports that males have higher susceptibility in cancer than females (41). By applying our risk model to the stratification analysis of gender, our 5-lncRNA signature also proved to be independent of gender.

To evaluate the predictive capacity of the 5-lncRNA signature, ROC analysis was carried out. In the diagnostic test, we can use AUROC to detect the accuracy and determine the predictive value of biomarkers (42). ROC analysis demonstrated that this 5-lncRNA signature was superior to the TNM stage in PDAC OS evaluation (in the entire TCGA and Fudan validation cohort), and histologic grade (in the entire TCGA cohort). Interestingly, when we combined the 5-lncRNA risk score with TNM stage and histologic grade (if available), the prognostic ability was better than any parameter alone in this model. The AUROC of the combined model reached 0.73 for the entire TCGA cohort, and 0.76 for the Fudan validation cohort, suggesting that it might enhance clinic-pathologic characteristics and strengthen the predictive accuracy of OS prognosis in PDAC.

Because the 5-lncRNA signature was able to separate high risk patients according to the risk score, we assumed that several biological functions and pathways related to this signature might affect the OS prognosis of PDAC. Nowadays, the main challenges for PDAC patients are metastatic problems and the lack of sensitive drugs for clinical treatment (1, 43), which seriously influence the prognosis of these patients. According to the analyses of cytoscape plug-in ClueGO and CluePedia, a number of cancer-related pathways were highlighted in the high-risk groups such as programmed cell death, regulation of cell population proliferation, epithelial cell differentiation, cell adhesion, and the secretion of pancreas pathway. Furthermore, several drug response pathways, signal transmission pathways, and the secretin receptors related to the pancreas were also found to be changed in the high-risk group. Therefore, these biological functions and pathways obtained from our 5-lncRNA signature might provide a strong backing for studying the molecular mechanisms and developing the potential targeted therapies.

The 5-lncRNA signature has been proven to be significantly connected with the overall survival of PDAC. However, the underlying function of these 5 lncRNAs have not been fully illuminated in this study. According to the negative coefficients and the downregulation of the five lncRNAs identified in high risk PDAC patients, we hypothesize that these lncRNAs are associated with better survival and may act as tumor suppressors in PDAC. RP11-159F24.5 was reported to be the antisense to nicotinamide nucleotide transhydrogenase (NNT) (44), and the deficiency of NNT will impede cell proliferation and tumorigenicity (45). RP11-744N12.2 is also named a smooth muscle and endothelial cell enriched migration/differentiation-associated long non-coding RNA (lncRNA SENCR) or FLI1-AS1 (46). Lyu et al. demonstrated that SENCR interacted with CKAP4 to stabilize vascular endothelial cell adherens junctions (47). RP11-388M20.1 is the antisense to PYCARD, and PYCRAFD is reported to play an important role in promoting cellular apoptosis (48). In addition, the expression of RP11-356C4.5 is found to be up-regulated in colorectal tumor samples compared to normal control tissues (49). There are extremely rare reports regarding CTC-459F4.9. More studies about this lncRNA are needed in the near future.

Several limitations in our study should be pointed out. First, our 5-lncRNA signature was generated from the retrospective data, and more prospective datasets are needed to prove the clinical utility of our model. Second, owing to the limited number of patients recruited in our study, some subgroup analyses cannot be implemented. Third, there was no experimental data about the expression and mechanisms of the lncRNAs in PDAC samples, so more efforts should be invested to illuminate the potential association between our signature and the prognosis in PDAC.

## Conclusion

In summary, our study defined an innovative 5-lncRNA signature in PDAC. It is an integrated analysis of the available RNA-sequencing data and qPCR results from our own samples. The 5-lncRNA signature was proven to be independently associated with the OS of classical prognostic parameters and remains a good classifier in different subtypes of PDAC patients. This signature may provide insight into the prediction of PDAC prognosis. What is more, the obtained functions and pathways associated with our signature may facilitate the development of novel therapies for PDAC treatment.

## Data Availability Statement

The level three RNA sequencing data and relevant clinical information of 177 PDAC patients were downloaded from TCGA database (http://cancergenome.nih.gov/). Other data generated or analyzed during this study are included in this published article and the Supplementary Material.

## Ethics Statement

Written informed consent was obtained from all our patients. The study was conducted in accordance with the Declaration of Helsinki, and the Ethical Committee of Huashan Hospital, Fudan University approved the study.

## Author Contributions

HJ, NR, and QD: conceptualization and funding acquisition. CZ: data curation. JZ, XX, and YZhe: formal analysis. WC, MX, and YY: investigation. SW, QZ, and MA: methodology. FY, DF, LQ, and HJ: resources. NR and QD: supervision. CZ, SW, and QZ: writing—original draft. YZha, CB, HJ, NR, and QD: writing—review and editing. All authors read and approved the final manuscript.

### Conflict of Interest

The authors declare that the research was conducted in the absence of any commercial or financial relationships that could be construed as a potential conflict of interest.

## Abbreviations

AUROC: Area under receiving operating curve
CI: Confidence interval
DEGs: Differentially expressed genes
FC: Fold change
HR: Hazard ratio
LASSO: least absolute shrinkage and selection operator
lincRNA: large intergenic non-coding RNAs
lncRNAs: long non-coding RNAs
MSI: microsatellite instability
MSI-I: MSI-indeterminate
MSI-L: MSI-low
MSS: microsatellite stable
OS: Overall survival
PC: pancreatic cancer
PDAC: Pancreatic ductal adenocarcinoma
ROC: Receiver operating characteristic curve
TCGA: The Cancer Genome Atlas
TNM: Tumor Node Metastasis.
