# Supplementary material for: A Long Non-coding RNA Signature to Improve Prognostic Prediction of Pancreatic Ductal Adenocarcinoma
Source: Front Oncol. 2019 Nov 8;9:1160. doi: 10.3389/fonc.2019.01160 (PMC6857660; doi:10.3389/fonc.2019.01160)
Supplement: Supplementary file 1 [file Data_Sheet_1.pdf]

# *Supplementary Material*

## Supplementary Figures

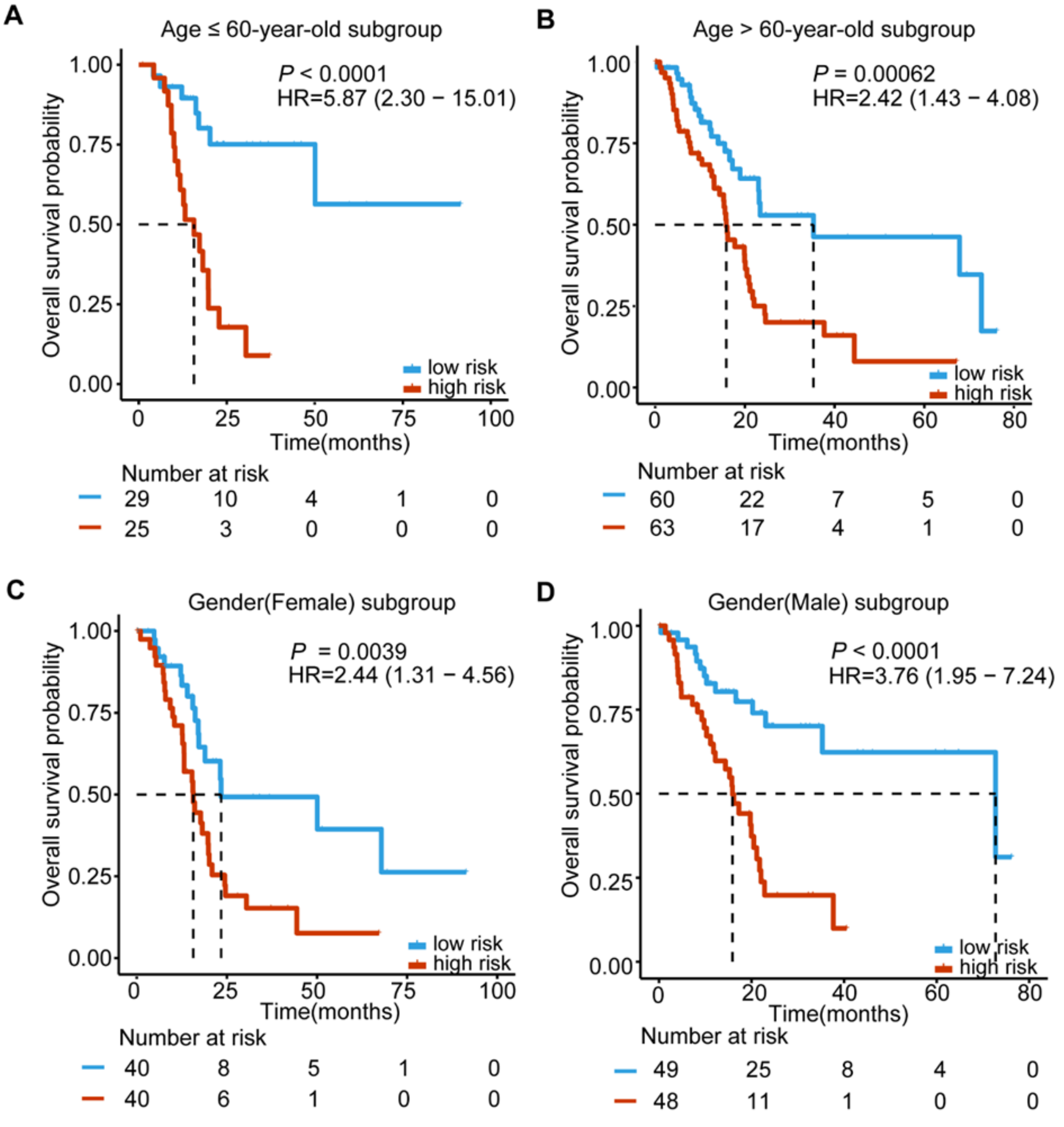


**Figure S1.** Kaplan–Meier survival analysis to assess the independence of the 5-lncRNA signature from age and gender. The patients from the entire TCGA were stratified into subgroups. The 5-lncRNA signature was applied to ≤60-year-old patients (A), >60-year-old patients (B), female patients (C), and male patients (D), separately. The number of patients at risk is listed below the survival curves. The tick marks on the Kaplan–Meier curves represents the censored subjects. Two-sided log-rank test was adopted to determine the differences between the two curves.

**Supplementary Tables**

**Table S1.** Primers of five lncRNAs and internal control for qRT-PCR.

| **Primers** | | **5'→3'** |
|---|---|---|
| RP11-159F24.5 | Forward | CCAGCCGAGAACTAACCACA |
| | Reverse | TGGCACCCATACACTTGCTC |
| RP11-744N12.2 | Forward | CTCTGGACCGTCCTCCCTAC |
| | Reverse | ACCTGTTTTGGATGTCCGGG |
| RP11-388M20.1 | Forward | CACCCATGTTAGCGGCAAGA |
| | Reverse | ACTTTGTCGTTGGCTCCAGA |
| RP11-356C4.5 | Forward | ACCGACCGGAGACCATTTG |
| | Reverse | TCAGCTGCACTGTCCAGAAAT |
| CTC-459F4.9 | Forward | TCATGAGCAGAAGCCTCACG |
| | Reverse | AGGTGTTTGAACAACTGAACTGA |
| ACTB | Forward | GGACCTGACTGACTACCTCAT |
| | Reverse | CGTAGCACAGCTTCTCCTTAAT |

**Table S2.** Top 1000 significantly up-regulated DEGs in high versus low-risk PDAC patients in TCGA dataset

| Gene symbol | logFC | AveExpr | t | Fold Change (FC) |
|---|---|---|---|---|
| PNLIP | 2.045237 | 8.453061 | 2.17314 | 4.12741 |
| TRY6 | 1.936282 | 7.877335 | 2.924158 | 3.82718 |
| PRSS1 | 1.932067 | 9.50307 | 2.584373 | 3.816017 |
| KLK1 | 1.836875 | 4.869373 | 3.719546 | 3.572355 |
| PRSS3 | 1.831064 | 7.309151 | 4.558847 | 3.557995 |
| CLPS | 1.800149 | 7.168339 | 2.144448 | 3.482562 |
| GP2 | 1.747505 | 7.158523 | 2.259119 | 3.357774 |
| CELA2A | 1.712074 | 6.783706 | 1.929192 | 3.276315 |
| CPA1 | 1.705388 | 8.563021 | 1.90517 | 3.261167 |
| CLDN18 | 1.675434 | 8.244075 | 2.829766 | 3.194155 |
| CELA3B | 1.651266 | 7.094141 | 1.927081 | 3.141091 |
| CELA3A | 1.639917 | 8.746538 | 1.802875 | 3.11648 |
| REG4 | 1.572373 | 8.039968 | 2.624788 | 2.973934 |
| CEACAM5 | 1.544647 | 7.380381 | 2.832229 | 2.917327 |
| CELP | 1.543434 | 3.674671 | 2.066348 | 2.914874 |
| CTRB2 | 1.510808 | 9.171878 | 1.63593 | 2.849696 |
| PLA2G1B | 1.468344 | 5.978326 | 1.887268 | 2.767041 |
| CPB1 | 1.462721 | 7.696889 | 1.701681 | 2.756277 |
| IL22RA1 | 1.452328 | 3.720659 | 5.757311 | 2.736494 |
| S100P | 1.441578 | 5.436965 | 3.282831 | 2.716179 |
| CELA2B | 1.426758 | 5.360154 | 1.839683 | 2.688419 |
| AMY2A | 1.417243 | 6.080361 | 1.702134 | 2.670747 |
| TNNT1 | 1.409591 | 0.431291 | 3.405826 | 2.656619 |
| CTRB1 | 1.395167 | 8.510534 | 1.543311 | 2.63019 |
| MUC17 | 1.387793 | 7.150697 | 2.378906 | 2.616781 |
| SYCN | 1.379225 | 5.298962 | 1.859538 | 2.601285 |
| REG3A | 1.361771 | 8.532898 | 1.708451 | 2.570006 |
| CPA2 | 1.356443 | 6.727316 | 1.614509 | 2.560531 |
| CTRC | 1.356094 | 7.267493 | 1.615037 | 2.559912 |
| KLK7 | 1.345123 | 4.540572 | 2.79319 | 2.540518 |
| FER1L6 | 1.338335 | 4.602734 | 2.779934 | 2.528593 |
| KLK8 | 1.338004 | 3.185317 | 2.807883 | 2.528013 |
| CLCA1 | 1.331749 | 2.611451 | 2.387968 | 2.517076 |
| COL17A1 | 1.31386 | 4.283096 | 3.049201 | 2.486059 |
| SERPINB5 | 1.293013 | 4.339273 | 3.599424 | 2.450393 |
| B3GNT6 | 1.277622 | 3.791894 | 2.782279 | 2.42439 |
| ANXA10 | 1.268813 | 8.148976 | 2.927272 | 2.409632 |
| PSAPL1 | 1.26565 | 3.235157 | 2.883912 | 2.404356 |
| REG1A | 1.265105 | 9.807173 | 1.776378 | 2.403446 |
| TM4SF20 | 1.258912 | 3.466394 | 2.522031 | 2.393152 |
| CA12 | 1.255527 | -0.25065 | 4.597573 | 2.387544 |
| EREG | 1.226257 | 2.792088 | 3.371004 | 2.339593 |
| KRT20 | 1.20095 | 2.633724 | 2.143313 | 2.29891 |

| | | | | |
|---|---|---|---|---|
| PNLIPRP2 | 1.188325 | 5.814802 | 1.5164 | 2.278881 |
| RASEF | 1.187435 | 1.79485 | 4.01505 | 2.277475 |
| ZG16 | 1.179663 | 0.956104 | 3.527917 | 2.265239 |
| RNF186 | 1.170385 | 3.244428 | 4.222422 | 2.250718 |
| DHRS9 | 1.168287 | 3.243168 | 2.946626 | 2.247446 |
| PNLIPRP1 | 1.163862 | 5.978338 | 1.484242 | 2.240564 |
| REG1B | 1.157885 | 7.161506 | 1.400606 | 2.231301 |
| DSG3 | 1.155498 | 2.196709 | 2.539544 | 2.227612 |
| GABRP | 1.154746 | 5.274919 | 2.554025 | 2.226451 |
| FAM83A | 1.151405 | 3.29532 | 2.67913 | 2.221302 |
| SLC6A14 | 1.132297 | 5.08291 | 2.85339 | 2.192075 |
| C12orf36 | 1.122629 | 5.128698 | 2.892948 | 2.177434 |
| KBTBD12 | 1.120947 | 2.995614 | 3.296496 | 2.174897 |
| SERPINI2 | 1.112764 | 3.386536 | 1.835151 | 2.162596 |
| SDR16C5 | 1.108183 | 4.381438 | 3.329082 | 2.15574 |
| HMGA2 | 1.102451 | 1.877789 | 3.357439 | 2.147191 |
| CEL | 1.088219 | 4.669586 | 1.448419 | 2.126115 |
| KLK11 | 1.085085 | 4.590395 | 3.006377 | 2.1215 |
| TMEM52 | 1.078618 | 0.708351 | 3.704898 | 2.112012 |
| CTSE | 1.07263 | 8.917552 | 2.378092 | 2.103264 |
| NIPAL1 | 1.069147 | 0.829704 | 5.379772 | 2.098193 |
| CUZD1 | 1.067097 | 3.754478 | 2.074998 | 2.095214 |
| AQP8 | 1.063333 | 3.078192 | 1.798379 | 2.089754 |
| DPCR1 | 1.050901 | 8.331693 | 1.75928 | 2.071824 |
| CEACAM6 | 1.049887 | 7.632522 | 2.520969 | 2.070367 |
| LIPH | 1.046411 | 3.802355 | 4.364037 | 2.065386 |
| UGT1A1 | 1.041587 | 2.252977 | 2.701012 | 2.058491 |
| OVOL1 | 1.03308 | 1.258374 | 3.640215 | 2.046389 |
| MT1H | 1.03163 | 1.290242 | 3.256935 | 2.044332 |
| GALNT5 | 1.024633 | 3.823432 | 3.302446 | 2.034441 |
| GPRC5A | 1.019827 | 3.377046 | 2.946854 | 2.027676 |
| TMPRSS4 | 1.015572 | 5.586462 | 2.602024 | 2.021704 |
| C6orf127 | 1.013906 | 0.944088 | 3.050764 | 2.019371 |
| SPINK1 | 1.013341 | 8.9673 | 2.667077 | 2.01858 |
| MYBPC1 | 1.001539 | 0.511259 | 1.998928 | 2.002135 |
| DKK1 | 0.99502 | 3.023303 | 2.60283 | 1.993109 |
| ITLN1 | 0.992204 | 2.043106 | 1.883063 | 1.989221 |
| GSTA2 | 0.987485 | 2.212679 | 2.383432 | 1.982725 |
| SERPINB3 | 0.986562 | 2.56507 | 1.995614 | 1.981458 |
| PTF1A | 0.983989 | 2.018834 | 2.395128 | 1.977927 |
| PLA2G10 | 0.982515 | 4.708538 | 3.095579 | 1.975907 |
| NTSR1 | 0.978507 | 3.379295 | 2.426189 | 1.970425 |
| VSIG1 | 0.975145 | 4.864617 | 2.210536 | 1.965839 |
| UGT1A10 | 0.968658 | 6.646577 | 2.130871 | 1.95702 |
| C6orf222 | 0.967486 | 5.913323 | 2.948966 | 1.95543 |
| POF1B | 0.96675 | 4.090089 | 3.742872 | 1.954434 |

| | | | | |
|---|---|---|---|---|
| REG3G | 0.960502 | 5.520569 | 1.296624 | 1.945987 |
| TCN1 | 0.959011 | 7.266813 | 2.309854 | 1.943977 |
| MYEOV | 0.950976 | 4.806619 | 2.614094 | 1.93318 |
| RAB27B | 0.950622 | 1.618789 | 4.742788 | 1.932706 |
| LOC84740 | 0.949802 | 4.530855 | 2.362915 | 1.931607 |
| GKN2 | 0.94896 | 0.864808 | 2.821109 | 1.93048 |
| PTPRR | 0.945743 | 2.872663 | 4.235012 | 1.926181 |
| TMPRSS2 | 0.944387 | 3.328939 | 4.219548 | 1.924371 |
| TFF2 | 0.943765 | 8.588441 | 1.909446 | 1.923541 |
| GJB4 | 0.942718 | 4.102233 | 2.852665 | 1.922146 |
| PLA2G2F | 0.941451 | 1.397294 | 2.680619 | 1.920459 |
| REG1P | 0.939568 | 3.724706 | 1.589798 | 1.917954 |
| PDIA2 | 0.939336 | 4.076639 | 1.795237 | 1.917646 |
| LGALS9B | 0.938744 | 1.540188 | 2.978236 | 1.916859 |
| FAM160A1 | 0.9368 | 0.322419 | 6.534918 | 1.914278 |
| GRHL2 | 0.926936 | 1.798755 | 3.585336 | 1.901234 |
| BCL2L14 | 0.92171 | 3.159743 | 3.48345 | 1.89436 |
| INHBA | 0.914457 | 2.583742 | 3.3976 | 1.884859 |
| MFSD4 | 0.914169 | 1.809884 | 4.58528 | 1.884483 |
| TMC5 | 0.913916 | 5.071053 | 3.210688 | 1.884152 |
| GUCA1C | 0.912901 | 1.215964 | 2.747557 | 1.882828 |
| CST1 | 0.909541 | 5.39497 | 2.190184 | 1.878448 |
| ATP10B | 0.905309 | 4.294987 | 2.614667 | 1.872946 |
| MUC4 | 0.904553 | 3.960972 | 2.179851 | 1.871964 |
| MT1G | 0.899999 | 1.405627 | 2.838821 | 1.866065 |
| TFF1 | 0.89616 | 7.966299 | 1.766033 | 1.861105 |
| POU2F3 | 0.889619 | 2.190491 | 3.042959 | 1.852687 |
| AQP5 | 0.889275 | 4.962929 | 2.136345 | 1.852246 |
| HOXA13 | 0.888346 | 0.964563 | 2.190174 | 1.851052 |
| PIGR | 0.888136 | 4.95877 | 2.092307 | 1.850783 |
| RPL3L | 0.887931 | 0.245597 | 3.197851 | 1.85052 |
| PROM1 | 0.88725 | 3.923407 | 2.715317 | 1.849647 |
| ITGB6 | 0.885785 | 4.245603 | 2.50132 | 1.847769 |
| SFN | 0.881216 | 2.159017 | 2.553622 | 1.841927 |
| SULT1B1 | 0.880216 | 3.483334 | 2.621595 | 1.840651 |
| GPHA2 | 0.878487 | 1.465142 | 2.204613 | 1.838446 |
| CA9 | 0.876063 | 3.926881 | 2.181182 | 1.83536 |
| CCK | 0.872326 | 2.531196 | 2.276491 | 1.830613 |
| GREM1 | 0.872313 | 4.284427 | 2.683446 | 1.830595 |
| TRIM29 | 0.864428 | 3.28395 | 2.033675 | 1.820618 |
| SLC7A11 | 0.862562 | 0.882459 | 4.29852 | 1.818265 |
| KLK10 | 0.859477 | 4.604143 | 2.087932 | 1.814381 |
| PRSS8 | 0.85628 | 2.29208 | 2.703458 | 1.810364 |
| RBPJL | 0.856249 | 3.253398 | 1.501925 | 1.810325 |
| GJB5 | 0.85553 | 1.851036 | 2.343432 | 1.809424 |
| C4orf31 | 0.854169 | 2.006111 | 3.382072 | 1.807717 |

| | | | | |
|---|---|---|---|---|
| FERMT1 | 0.853051 | 2.840035 | 3.650136 | 1.806317 |
| SYTL5 | 0.849604 | 2.021682 | 2.903647 | 1.802006 |
| CAPN9 | 0.844943 | 3.748889 | 2.008684 | 1.796194 |
| PGC | 0.844704 | 5.560279 | 1.255339 | 1.795896 |
| CXADR | 0.841587 | 0.306294 | 4.970806 | 1.79202 |
| TNS4 | 0.837799 | 3.29951 | 1.971267 | 1.787321 |
| IL8 | 0.836707 | 2.026944 | 2.976542 | 1.785969 |
| PRSS21 | 0.836214 | 2.213042 | 2.093533 | 1.785359 |
| CDKL5 | 0.836006 | 0.334445 | 3.607123 | 1.785102 |
| PLS1 | 0.835158 | 2.662009 | 4.997824 | 1.784053 |
| GJB6 | 0.834507 | 0.342266 | 2.39603 | 1.783247 |
| GKN1 | 0.834312 | 1.433918 | 2.163139 | 1.783007 |
| AQP12B | 0.831016 | 3.242953 | 1.884399 | 1.778938 |
| XK | 0.830579 | 1.151301 | 4.315545 | 1.778399 |
| ST6GALNAC1 | 0.829691 | 4.07503 | 2.559686 | 1.777305 |
| PSCA | 0.827775 | 5.297038 | 1.536011 | 1.774946 |
| LOC284578 | 0.827017 | 1.109339 | 3.671941 | 1.774014 |
| LGALS4 | 0.825858 | 8.279622 | 2.103017 | 1.772589 |
| KCTD14 | 0.824236 | 1.20242 | 4.173201 | 1.770597 |
| FUT3 | 0.819629 | 4.754432 | 2.723474 | 1.764952 |
| MET | 0.817474 | 1.352748 | 4.385717 | 1.762317 |
| TSPAN8 | 0.814402 | 6.983448 | 2.6331 | 1.758569 |
| UCA1 | 0.813314 | 4.066285 | 2.117989 | 1.757243 |
| FA2H | 0.811204 | 3.798353 | 3.741077 | 1.754675 |
| BCAS1 | 0.807348 | 3.948085 | 2.491267 | 1.749992 |
| TRPA1 | 0.805426 | 0.596266 | 3.114298 | 1.747662 |
| SLC6A20 | 0.802443 | 5.195894 | 2.748006 | 1.744052 |
| ZNF488 | 0.80203 | 1.661028 | 3.34535 | 1.743552 |
| GGT6 | 0.801249 | 2.756491 | 2.513291 | 1.74261 |
| PLEK2 | 0.801035 | 2.420527 | 3.059009 | 1.74235 |
| FMN1 | 0.798599 | 1.594724 | 4.171465 | 1.739411 |
| AQP12A | 0.798564 | 1.285981 | 2.43861 | 1.739369 |
| SCNN1A | 0.793061 | 2.678335 | 2.990431 | 1.732747 |
| AKR7A3 | 0.791122 | 3.292027 | 3.012125 | 1.730419 |
| CYP2S1 | 0.790862 | 2.931377 | 2.566307 | 1.730108 |
| EHF | 0.790309 | 2.889067 | 3.732564 | 1.729444 |
| AGR2 | 0.787031 | 5.640002 | 2.383154 | 1.72552 |
| GATM | 0.786502 | 1.89692 | 2.882587 | 1.724887 |
| EGF | 0.785981 | -0.45758 | 2.141989 | 1.724265 |
| OASL | 0.78577 | 1.441034 | 3.097817 | 1.724013 |
| MUC5B | 0.785727 | 6.585296 | 1.788664 | 1.723961 |
| SLC28A3 | 0.784503 | 3.628833 | 2.574978 | 1.722499 |
| C11orf86 | 0.784252 | 3.2291 | 2.194782 | 1.722199 |
| STX19 | 0.780797 | 2.157941 | 3.606698 | 1.718079 |
| ALDH3A1 | 0.780318 | 1.88238 | 2.167104 | 1.71751 |
| FAM83B | 0.77989 | 2.245068 | 3.315089 | 1.716999 |

| | | | | |
|---|---|---|---|---|
| HHIP | 0.777465 | 2.332269 | 2.914495 | 1.714117 |
| APOBEC1 | 0.771732 | 4.451296 | 2.136908 | 1.707318 |
| ARL14 | 0.771596 | 6.141638 | 2.307938 | 1.707158 |
| ACER2 | 0.769909 | 0.562279 | 3.90462 | 1.705163 |
| AMY1A | 0.768485 | 0.665036 | 1.689157 | 1.70348 |
| BHLHA15 | 0.766278 | 1.035745 | 2.445375 | 1.700876 |
| LAMA3 | 0.763877 | 3.209131 | 3.445911 | 1.698047 |
| PLAC8 | 0.763575 | 3.724412 | 3.128725 | 1.697692 |
| ZG16B | 0.761853 | 3.604737 | 2.556861 | 1.695668 |
| IMPA2 | 0.760173 | 0.395199 | 5.078067 | 1.693694 |
| ACTBL2 | 0.759749 | 2.047662 | 3.140496 | 1.693196 |
| ANKRD22 | 0.759278 | 2.222103 | 3.140936 | 1.692643 |
| DSG2 | 0.753074 | 1.838189 | 5.901564 | 1.68538 |
| ERBB3 | 0.752371 | 1.100858 | 4.578012 | 1.684559 |
| HKDC1 | 0.750958 | 4.071056 | 2.674721 | 1.68291 |
| GALNT3 | 0.750455 | 2.260315 | 4.356902 | 1.682323 |
| ESRP1 | 0.750446 | 2.110413 | 5.248754 | 1.682313 |
| HNF4G | 0.750176 | 3.35369 | 3.370535 | 1.681998 |
| COL10A1 | 0.748453 | 5.035225 | 2.065399 | 1.679991 |
| UNC93A | 0.746034 | 0.260528 | 3.266648 | 1.677176 |
| SPRR1B | 0.741036 | 2.460727 | 1.565855 | 1.671375 |
| GCNT3 | 0.739258 | 6.293309 | 2.434519 | 1.669317 |
| S100A14 | 0.73863 | 2.841647 | 2.131269 | 1.66859 |
| SPINK4 | 0.738422 | 3.042445 | 1.473921 | 1.66835 |
| ARHGEF38 | 0.737851 | 1.872054 | 3.696776 | 1.66769 |
| FUT6 | 0.735916 | 4.47464 | 2.697692 | 1.665454 |
| SPRR1A | 0.735574 | 1.93355 | 1.692724 | 1.66506 |
| EGLN3 | 0.73552 | 1.161731 | 2.977771 | 1.664998 |
| CTRL | 0.734328 | 3.120654 | 1.301015 | 1.663623 |
| FCGBP | 0.731513 | 1.726929 | 2.339954 | 1.660379 |
| MICALCL | 0.730419 | 0.851949 | 4.029425 | 1.659121 |
| SLC13A5 | 0.729278 | 3.424935 | 1.642753 | 1.657809 |
| TMEM97 | 0.729218 | 0.313144 | 4.213466 | 1.65774 |
| DMBT1 | 0.727514 | 6.680586 | 1.480225 | 1.655783 |
| LPAR3 | 0.726265 | -1.02611 | 3.155393 | 1.65435 |
| SI | 0.725846 | 1.638453 | 1.499073 | 1.65387 |
| FABP1 | 0.725605 | 1.350415 | 1.565963 | 1.653594 |
| LGR4 | 0.725384 | 0.663399 | 5.543613 | 1.653341 |
| GPR81 | 0.724389 | 0.408054 | 2.598652 | 1.6522 |
| LAMB3 | 0.724064 | 3.156419 | 2.352671 | 1.651829 |
| B3GNT7 | 0.721954 | 2.51758 | 3.357541 | 1.649414 |
| LRG1 | 0.72192 | 2.017164 | 3.036023 | 1.649376 |
| C1orf106 | 0.72062 | 3.029083 | 2.347709 | 1.64789 |
| KIAA0754 | 0.719802 | 0.934562 | 2.974955 | 1.646956 |
| RPSAP52 | 0.718957 | 1.64063 | 3.303137 | 1.645992 |
| KLF5 | 0.718874 | 2.301756 | 3.537086 | 1.645896 |

| | | | | |
|---|---|---|---|---|
| DAZ1 | 0.718345 | 0.720196 | 1.508911 | 1.645294 |
| B3GALT5 | 0.717454 | 4.828087 | 2.882994 | 1.644278 |
| CASP14 | 0.717328 | -0.27336 | 2.421431 | 1.644135 |
| SLC9A4 | 0.714971 | 2.525387 | 1.662653 | 1.64145 |
| HOXB9 | 0.71486 | 1.47862 | 1.895681 | 1.641323 |
| CREB3L3 | 0.714015 | 2.062091 | 1.877675 | 1.640362 |
| VSIG2 | 0.712668 | 4.8949 | 2.033857 | 1.638833 |
| ERO1L | 0.711405 | 0.919494 | 4.96189 | 1.637399 |
| UHMK1 | 0.709776 | -0.07784 | 3.617921 | 1.63555 |
| LRRC31 | 0.707094 | 2.115215 | 1.885381 | 1.632512 |
| FAT1 | 0.706413 | 0.97259 | 5.217492 | 1.631742 |
| MMP3 | 0.704618 | 1.733379 | 2.241859 | 1.629713 |
| BTNL8 | 0.703068 | 4.047771 | 1.738412 | 1.627963 |
| C3orf52 | 0.701596 | 1.795933 | 3.799715 | 1.626303 |
| IYD | 0.701528 | 3.351696 | 2.05725 | 1.626227 |
| PDZK1IP1 | 0.700412 | 3.24739 | 2.264458 | 1.624969 |
| EPYC | 0.699954 | 2.783277 | 1.506051 | 1.624453 |
| GATA4 | 0.699084 | 5.342343 | 2.22677 | 1.623473 |
| ADAMTS12 | 0.69841 | 2.45889 | 2.450477 | 1.622716 |
| IGF2BP3 | 0.696922 | 1.487328 | 2.21448 | 1.621042 |
| TNFRSF11B | 0.696893 | 1.950092 | 2.617412 | 1.62101 |
| TMEM45B | 0.696427 | 2.706634 | 3.02194 | 1.620486 |
| AADAC | 0.696127 | 3.983295 | 2.138193 | 1.620149 |
| TRHDE | 0.695157 | 1.433749 | 2.294787 | 1.61906 |
| PTK6 | 0.694075 | 2.80799 | 2.523665 | 1.617846 |
| TNFSF15 | 0.69329 | 0.557251 | 3.43609 | 1.616967 |
| TLR3 | 0.692855 | 0.736252 | 3.913288 | 1.616479 |
| RNF39 | 0.692639 | 1.726949 | 3.012022 | 1.616237 |
| STON1-GTF2A1L | 0.68967 | 0.664282 | 2.377286 | 1.612915 |
| ANKRD56 | 0.686627 | 1.204111 | 4.862424 | 1.609516 |
| KPNA7 | 0.686096 | 3.383165 | 2.717433 | 1.608923 |
| SERINC5 | 0.686035 | 0.134304 | 3.356832 | 1.608855 |
| TFAP2C | 0.685765 | -1.48584 | 2.484176 | 1.608554 |
| FAM59A | 0.685516 | 0.414642 | 5.535136 | 1.608277 |
| GJB2 | 0.684578 | 2.144397 | 2.242873 | 1.607232 |
| SPINK5 | 0.683616 | 1.738375 | 2.383487 | 1.606161 |
| CLDN4 | 0.683117 | 2.584789 | 3.404618 | 1.605605 |
| GPT2 | 0.680258 | -0.44675 | 4.533785 | 1.602427 |
| LAMC2 | 0.67829 | 4.129341 | 2.392868 | 1.600242 |
| DUSP4 | 0.678104 | 1.926457 | 5.057686 | 1.600036 |
| FAM83D | 0.677665 | -0.16084 | 3.226678 | 1.599549 |
| PVRL4 | 0.677591 | 2.500103 | 2.113544 | 1.599466 |
| EPPK1 | 0.677534 | 1.056871 | 2.919391 | 1.599404 |
| MOGAT3 | 0.67629 | 1.545655 | 2.059407 | 1.598025 |
| GJB1 | 0.676288 | 3.801664 | 2.509042 | 1.598023 |
| HOXC11 | 0.675604 | 0.633794 | 1.729635 | 1.597265 |

| | | | | |
|---|---|---|---|---|
| OCLN | 0.674844 | 1.195066 | 4.861067 | 1.596424 |
| GPA33 | 0.673705 | 3.173669 | 2.114416 | 1.595164 |
| RHBDL2 | 0.672063 | 2.196133 | 3.035549 | 1.59335 |
| KIAA1199 | 0.670586 | 2.694494 | 2.555586 | 1.591719 |
| SLC9A2 | 0.667781 | 1.790246 | 1.97601 | 1.588628 |
| KRT6A | 0.667545 | 2.675337 | 1.199406 | 1.588368 |
| IGF2BP2 | 0.665801 | 1.48543 | 3.105044 | 1.586449 |
| LOC100127888 | 0.665675 | 3.204526 | 2.332944 | 1.58631 |
| MEP1A | 0.665434 | 1.139519 | 1.711766 | 1.586045 |
| XDH | 0.664677 | 3.205719 | 2.37819 | 1.585214 |
| CDH3 | 0.664335 | 1.795385 | 2.734607 | 1.584838 |
| FCGR3B | 0.663758 | 1.112422 | 2.039845 | 1.584204 |
| COL11A1 | 0.661684 | 4.097805 | 1.470785 | 1.581928 |
| SLC39A5 | 0.661289 | 3.962246 | 1.614617 | 1.581495 |
| FUT2 | 0.661115 | 3.604669 | 3.274475 | 1.581304 |
| B4GALNT2 | 0.660952 | -0.77716 | 2.44977 | 1.581126 |
| MST1R | 0.660693 | 3.502768 | 2.438335 | 1.580842 |
| MIA2 | 0.660335 | 3.813104 | 2.74031 | 1.58045 |
| AGR3 | 0.660162 | 4.888252 | 1.996641 | 1.58026 |
| ATP8B1 | 0.659384 | 1.161809 | 5.287673 | 1.579408 |
| ADAM9 | 0.658518 | 1.158792 | 4.899471 | 1.578461 |
| RASSF6 | 0.658215 | 3.020569 | 4.051497 | 1.578129 |
| MUC1 | 0.656737 | 3.668089 | 2.310594 | 1.576513 |
| PRR11 | 0.656678 | -0.46857 | 2.730385 | 1.576449 |
| GJB3 | 0.654593 | 3.766767 | 2.028668 | 1.574172 |
| C14orf34 | 0.653174 | 1.561835 | 2.359971 | 1.572624 |
| CXCL17 | 0.653038 | 4.054921 | 1.635924 | 1.572476 |
| C20orf114 | 0.652447 | 3.187088 | 1.233773 | 1.571832 |
| PPP1R1B | 0.648959 | 3.776974 | 1.761348 | 1.568037 |
| NPR3 | 0.647967 | 1.334424 | 2.516089 | 1.566958 |
| FOXA3 | 0.647657 | 5.738747 | 2.823994 | 1.566622 |
| TMEM92 | 0.646426 | 3.615664 | 2.389926 | 1.565285 |
| MYO5B | 0.645632 | 0.939487 | 5.121354 | 1.564425 |
| IRF6 | 0.645052 | 1.293044 | 4.45604 | 1.563796 |
| LY6G6C | 0.644773 | 0.086965 | 2.494272 | 1.563493 |
| MAP3K13 | 0.644649 | 0.976964 | 4.511612 | 1.563359 |
| ANXA3 | 0.644024 | 2.642917 | 2.675838 | 1.562682 |
| GATA5 | 0.643816 | -0.10967 | 1.849842 | 1.562457 |
| TEX11 | 0.643593 | 2.115248 | 2.178797 | 1.562215 |
| UGT8 | 0.642829 | 2.539979 | 3.437438 | 1.561388 |
| CLCN1 | 0.642406 | 2.170034 | 2.253366 | 1.560931 |
| CXCL5 | 0.641171 | 5.493161 | 1.367279 | 1.559595 |
| AREG | 0.640597 | 2.705624 | 2.544574 | 1.558974 |
| DKK4 | 0.640165 | 2.05027 | 2.053222 | 1.558508 |
| XKR9 | 0.639639 | 1.763238 | 2.813322 | 1.557939 |
| ITGA2 | 0.639466 | 2.10116 | 3.723966 | 1.557752 |

| | | | | |
|---|---|---|---|---|
| PADI1 | 0.639412 | 5.264886 | 1.283342 | 1.557695 |
| WNT2 | 0.638505 | 2.579734 | 2.122486 | 1.556715 |
| PITX1 | 0.638394 | 2.245732 | 1.946102 | 1.556595 |
| MBNL3 | 0.637174 | 0.370933 | 3.626337 | 1.55528 |
| MPZL3 | 0.636587 | 1.010735 | 3.489109 | 1.554647 |
| MAL2 | 0.636465 | 2.175277 | 3.597943 | 1.554515 |
| B3GNT3 | 0.636334 | 4.657438 | 3.019204 | 1.554375 |
| HAS3 | 0.635795 | 0.205544 | 2.81179 | 1.553794 |
| HSD17B2 | 0.635695 | 5.542528 | 2.0727 | 1.553686 |
| LRRC8E | 0.634913 | 0.6861 | 3.474176 | 1.552844 |
| GPR109B | 0.633873 | -0.35825 | 2.386782 | 1.551725 |
| KLK6 | 0.63308 | 4.410482 | 1.324136 | 1.550872 |
| NBEAL1 | 0.632954 | 0.623042 | 3.50319 | 1.550737 |
| HRASLS2 | 0.63189 | 2.410228 | 1.920653 | 1.549594 |
| LPP | 0.631889 | 0.503351 | 3.975494 | 1.549593 |
| TNFRSF10A | 0.630553 | 1.272107 | 3.540466 | 1.548159 |
| ANLN | 0.628975 | 0.043592 | 2.88126 | 1.546466 |
| TMEM195 | 0.627184 | 2.103852 | 1.778942 | 1.544547 |
| MSLN | 0.62671 | 6.132755 | 1.38034 | 1.54404 |
| MUC21 | 0.626019 | 0.235175 | 1.807308 | 1.543301 |
| CGN | 0.625171 | 2.362616 | 3.926266 | 1.542394 |
| SSFA2 | 0.624357 | 0.82287 | 5.670749 | 1.541523 |
| FAM3D | 0.623736 | 4.58971 | 1.595317 | 1.54086 |
| FAM108C1 | 0.623629 | 1.238152 | 3.754574 | 1.540745 |
| GPR87 | 0.623175 | 2.140335 | 1.512558 | 1.540261 |
| MECOM | 0.622483 | 1.67198 | 3.813214 | 1.539523 |
| GALNT7 | 0.622466 | 0.612821 | 4.583137 | 1.539505 |
| CCL24 | 0.622276 | 2.385981 | 2.05968 | 1.539302 |
| PPAP2C | 0.621922 | 2.017492 | 2.982079 | 1.538924 |
| SH3RF2 | 0.621338 | 2.602507 | 2.313695 | 1.538301 |
| CYP2C18 | 0.619448 | 5.065958 | 1.78883 | 1.536287 |
| ITGA6 | 0.618514 | 1.112898 | 4.6275 | 1.535293 |
| SFTA2 | 0.618167 | 5.398023 | 1.733184 | 1.534924 |
| DNAJC22 | 0.618014 | 1.827042 | 3.477428 | 1.534761 |
| SHH | 0.617905 | 3.152606 | 2.261657 | 1.534645 |
| EPB41L4B | 0.616106 | 1.311254 | 3.684393 | 1.532732 |
| GDPD2 | 0.615164 | 1.603101 | 1.988632 | 1.531733 |
| SH2D4A | 0.614917 | 1.31121 | 3.732984 | 1.53147 |
| FGFBP1 | 0.614523 | 2.431399 | 1.676833 | 1.531052 |
| SLC30A2 | 0.614414 | 2.536901 | 1.463222 | 1.530936 |
| ZDHHC20 | 0.613426 | 0.562856 | 3.089375 | 1.529887 |
| EFNA2 | 0.612141 | 2.27501 | 2.185503 | 1.528525 |
| BTBD16 | 0.611073 | 1.853714 | 2.409784 | 1.527395 |
| UPK1B | 0.610071 | 3.261252 | 1.24176 | 1.526335 |
| LCOR | 0.610007 | 0.486156 | 3.260378 | 1.526267 |
| RHOF | 0.609535 | 1.822365 | 2.742177 | 1.525768 |

| | | | | |
|---|---|---|---|---|
| HK2 | 0.609314 | 0.504301 | 2.808375 | 1.525534 |
| ARL5B | 0.609018 | 0.014034 | 5.372308 | 1.525221 |
| TJP3 | 0.609013 | 3.110067 | 2.364741 | 1.525215 |
| SLC34A2 | 0.6072 | 1.752796 | 1.627656 | 1.5233 |
| TRIM31 | 0.60613 | 5.361354 | 1.669679 | 1.522171 |
| LOC153328 | 0.606086 | -0.89103 | 3.498691 | 1.522124 |
| DNAH3 | 0.604531 | 1.379064 | 2.476299 | 1.520484 |
| C4orf19 | 0.604169 | 2.62148 | 3.602077 | 1.520103 |
| SLC38A5 | 0.603431 | 1.774597 | 1.976457 | 1.519325 |
| ANXA13 | 0.603237 | 4.164542 | 1.810421 | 1.519121 |
| LCN2 | 0.602742 | 6.025128 | 1.419112 | 1.5186 |
| PAX7 | 0.601637 | -0.25776 | 2.081501 | 1.517437 |
| C19orf33 | 0.601512 | 3.930233 | 1.798227 | 1.517306 |
| DSC2 | 0.601116 | 0.869057 | 4.595714 | 1.516889 |
| ARNTL2 | 0.601021 | 1.69877 | 3.918156 | 1.51679 |
| IGSF9 | 0.600763 | 1.247899 | 2.184257 | 1.516518 |
| PRKAA2 | 0.600195 | 0.375168 | 3.067104 | 1.515921 |
| MSX2 | 0.599961 | 1.50654 | 2.642971 | 1.515676 |
| EPS8L3 | 0.599757 | 7.995032 | 1.753581 | 1.515461 |
| TBX4 | 0.598827 | -0.7799 | 2.881837 | 1.514485 |
| GAN | 0.596844 | 0.355477 | 4.79405 | 1.512404 |
| FAM25A | 0.596239 | 0.091045 | 2.993465 | 1.51177 |
| UGT2B7 | 0.595532 | 2.560373 | 1.708488 | 1.51103 |
| WBSCR26 | 0.595506 | 2.749392 | 2.227551 | 1.511002 |
| SPDEF | 0.594572 | 3.182807 | 1.863879 | 1.510025 |
| TACSTD2 | 0.594454 | 2.613173 | 1.906367 | 1.509901 |
| PTPLB | 0.593828 | -0.11404 | 3.264325 | 1.509246 |
| KRT13 | 0.593462 | 1.528508 | 1.302373 | 1.508864 |
| RHOV | 0.593225 | 1.530076 | 1.996961 | 1.508615 |
| SCEL | 0.591938 | 3.721138 | 1.536589 | 1.507271 |
| CHMP4C | 0.59174 | 1.657256 | 4.625498 | 1.507063 |
| PAQR5 | 0.590816 | 0.93351 | 3.053549 | 1.506099 |
| BCAT1 | 0.590701 | 0.908356 | 2.714207 | 1.505979 |
| GTF2A1 | 0.5894 | -0.2578 | 3.330031 | 1.504621 |
| REST | 0.588903 | -0.11475 | 2.981179 | 1.504103 |
| RHPN2 | 0.588303 | 1.383183 | 3.449288 | 1.503478 |
| DDX60 | 0.586341 | 0.728089 | 3.732333 | 1.501434 |
| PRB1 | 0.582678 | 0.838628 | 2.365371 | 1.497626 |
| SLC2A1 | 0.582608 | 0.916859 | 2.652926 | 1.497554 |
| HHLA2 | 0.582466 | 4.813129 | 1.769147 | 1.497406 |
| TMC7 | 0.582165 | 2.167489 | 3.00102 | 1.497095 |
| GIPC2 | 0.581772 | 2.592847 | 2.868421 | 1.496687 |
| ADAP1 | 0.581751 | 2.226106 | 2.968868 | 1.496665 |
| PTPN14 | 0.581718 | 0.237297 | 4.104599 | 1.49663 |
| MMP1 | 0.581534 | 4.791043 | 1.490463 | 1.496439 |
| GPR35 | 0.580982 | 3.464784 | 2.453684 | 1.495867 |

| | | | | |
|---|---|---|---|---|
| ZPLD1 | 0.580681 | 2.709147 | 1.964592 | 1.495555 |
| EPN3 | 0.580611 | 1.815645 | 2.210702 | 1.495483 |
| LOC93432 | 0.580584 | 2.91814 | 1.723067 | 1.495455 |
| KLK13 | 0.580057 | 0.179414 | 2.186225 | 1.494908 |
| KRT16 | 0.579105 | 3.931997 | 1.295048 | 1.493922 |
| SGMS2 | 0.578191 | 1.472175 | 4.62378 | 1.492976 |
| LEMD1 | 0.577023 | 3.450614 | 1.653288 | 1.491768 |
| PRAP1 | 0.576483 | 2.408642 | 1.209838 | 1.491209 |
| SLC2A10 | 0.576332 | 0.925115 | 3.74392 | 1.491054 |
| LUZP2 | 0.576128 | 0.954169 | 2.266743 | 1.490843 |
| SLCO4A1 | 0.575996 | 1.957324 | 2.657208 | 1.490707 |
| HAPLN1 | 0.575323 | -0.12153 | 1.891792 | 1.490011 |
| COL12A1 | 0.574744 | 2.209335 | 2.417476 | 1.489413 |
| PARD3B | 0.574732 | 0.941063 | 2.89118 | 1.4894 |
| DUOX2 | 0.574548 | 5.710105 | 1.339606 | 1.48921 |
| HOXA11 | 0.574087 | -0.6721 | 1.621904 | 1.488735 |
| EPHA2 | 0.573499 | 2.043712 | 2.779913 | 1.488128 |
| ECT2 | 0.572992 | 0.225647 | 4.056789 | 1.487605 |
| STRN | 0.572175 | -0.09829 | 3.815804 | 1.486763 |
| MGAT5 | 0.571185 | 0.419403 | 3.160451 | 1.485743 |
| GDA | 0.570788 | 4.615526 | 2.051207 | 1.485335 |
| PSAT1 | 0.570662 | -1.59861 | 2.689129 | 1.485205 |
| METTL11B | 0.570551 | 0.912487 | 3.064355 | 1.48509 |
| MOCOS | 0.570153 | 0.552712 | 3.130811 | 1.484681 |
| LONRF3 | 0.568848 | 1.363729 | 3.946491 | 1.483339 |
| NR1I2 | 0.568399 | 3.863247 | 1.544409 | 1.482877 |
| CXCL11 | 0.568103 | 0.110239 | 2.066337 | 1.482573 |
| CAPN8 | 0.568011 | 6.03774 | 1.726625 | 1.482479 |
| CD55 | 0.567947 | 1.806618 | 3.423416 | 1.482412 |
| TNNI3 | 0.566689 | 0.06548 | 1.794144 | 1.481121 |
| GREB1L | 0.56625 | 0.782567 | 2.243052 | 1.48067 |
| IHH | 0.56623 | 4.136121 | 1.600676 | 1.480649 |
| RASSF3 | 0.565809 | 0.398839 | 3.035156 | 1.480218 |
| HPDL | 0.563979 | 0.646751 | 2.630833 | 1.478341 |
| CXorf61 | 0.563949 | 0.700604 | 1.627215 | 1.478311 |
| TSPAN1 | 0.563702 | 3.3308 | 2.415437 | 1.478057 |
| INPP4B | 0.562316 | 1.976962 | 3.246121 | 1.476638 |
| PLA2G4F | 0.56114 | 0.390745 | 2.086722 | 1.475435 |
| TC2N | 0.559532 | 1.655375 | 4.233775 | 1.473791 |
| BTC | 0.559413 | 0.968372 | 3.218445 | 1.473669 |
| DDI2 | 0.558539 | -0.25755 | 3.00358 | 1.472777 |
| ABP1 | 0.558473 | 4.603355 | 1.863529 | 1.472709 |
| ETV3 | 0.558159 | 0.107157 | 3.699214 | 1.472389 |
| KANK4 | 0.557158 | 2.27644 | 1.934703 | 1.471368 |
| MACC1 | 0.556543 | 2.519015 | 2.905928 | 1.47074 |
| MUC2 | 0.556423 | 1.998925 | 0.97766 | 1.470619 |

| | | | | |
|---|---|---|---|---|
| MMP12 | 0.555823 | 2.633455 | 1.918414 | 1.470007 |
| FGFR2 | 0.555731 | 0.246294 | 2.435597 | 1.469913 |
| SLC25A21 | 0.55462 | -0.03377 | 3.109981 | 1.468781 |
| STYK1 | 0.554152 | 2.43433 | 2.403869 | 1.468305 |
| ATL3 | 0.553944 | 0.088122 | 5.25119 | 1.468094 |
| RNF128 | 0.552546 | 3.116316 | 2.99863 | 1.466671 |
| FAR2 | 0.552396 | 0.479825 | 3.346558 | 1.466519 |
| MFI2 | 0.552116 | 2.022681 | 2.267752 | 1.466235 |
| WBSCR27 | 0.552003 | 0.107293 | 2.509206 | 1.46612 |
| PI3 | 0.551595 | 3.36265 | 1.44501 | 1.465705 |
| DSC3 | 0.551548 | 0.117505 | 1.736702 | 1.465658 |
| TAOK1 | 0.551137 | 0.024357 | 3.09172 | 1.46524 |
| ACSL5 | 0.549917 | 1.949731 | 2.569944 | 1.464001 |
| CDHR2 | 0.54938 | 6.184954 | 1.474542 | 1.463456 |
| PRR15 | 0.548882 | 2.693412 | 2.646311 | 1.462951 |
| LOC201651 | 0.547127 | 1.766504 | 2.216637 | 1.461173 |
| DLGAP5 | 0.546841 | 0.164385 | 2.538123 | 1.460883 |
| EGFR | 0.546213 | 0.181938 | 3.282618 | 1.460248 |
| ANPEP | 0.545084 | 1.549054 | 1.833121 | 1.459105 |
| CLDN1 | 0.544616 | 1.772285 | 2.601091 | 1.458632 |
| BCL2L15 | 0.544356 | 5.124517 | 2.028791 | 1.458369 |
| TGFA | 0.544226 | 1.492761 | 2.915006 | 1.458238 |
| F3 | 0.543539 | 2.622616 | 2.905406 | 1.457543 |
| REP15 | 0.543397 | 0.767973 | 2.359718 | 1.4574 |
| HPGD | 0.541403 | 2.229396 | 2.147816 | 1.455388 |
| C17orf73 | 0.541095 | 2.943748 | 1.439571 | 1.455076 |
| CREB3L1 | 0.540266 | 3.118014 | 2.716214 | 1.45424 |
| PARD6B | 0.539961 | 0.420798 | 4.035204 | 1.453933 |
| GPR126 | 0.539795 | 1.719349 | 2.591849 | 1.453766 |
| ASXL2 | 0.539581 | -0.0826 | 3.170209 | 1.45355 |
| CEP55 | 0.538771 | 0.330255 | 2.743667 | 1.452735 |
| CHRM3 | 0.538706 | 0.41403 | 2.216054 | 1.452668 |
| C9orf152 | 0.537772 | 3.770629 | 1.922407 | 1.451729 |
| GDF15 | 0.537461 | 0.597834 | 2.132719 | 1.451416 |
| HNF4A | 0.536587 | 6.062346 | 2.449124 | 1.450537 |
| AZGP1 | 0.536163 | 2.074016 | 1.77724 | 1.450111 |
| CDCP1 | 0.536139 | 2.110076 | 4.047572 | 1.450087 |
| VWA2 | 0.535007 | 2.561477 | 2.380144 | 1.448949 |
| NEK2 | 0.534967 | 0.135263 | 2.660174 | 1.448909 |
| TCEA3 | 0.533922 | 0.368868 | 2.911265 | 1.44786 |
| SEL1L | 0.533793 | 0.263556 | 4.98651 | 1.44773 |
| RASAL1 | 0.533629 | 2.469089 | 2.146774 | 1.447565 |
| DENND1B | 0.533022 | -0.02028 | 3.676369 | 1.446957 |
| LGALS3 | 0.532893 | 1.326822 | 2.936546 | 1.446828 |
| DLX3 | 0.532609 | -1.12655 | 2.379257 | 1.446543 |
| SGK196 | 0.532363 | -0.2547 | 3.191676 | 1.446296 |

| | | | | |
|---|---|---|---|---|
| PRSS12 | 0.53157 | 2.60815 | 2.63622 | 1.445502 |
| HIST1H2BN | 0.531417 | -0.62388 | 3.639439 | 1.445348 |
| NMU | 0.530906 | 2.628939 | 1.463274 | 1.444836 |
| NFE2L3 | 0.53042 | 1.000828 | 3.987689 | 1.44435 |
| F2RL1 | 0.529548 | 2.279772 | 3.301408 | 1.443477 |
| BANF2 | 0.529213 | 0.283466 | 3.387859 | 1.443142 |
| C7orf10 | 0.528765 | 1.10071 | 2.191949 | 1.442694 |
| LANCL3 | 0.528673 | 1.055643 | 3.092469 | 1.442602 |
| TYRP1 | 0.528501 | 0.771528 | 2.112473 | 1.44243 |
| COL8A1 | 0.527871 | 2.031525 | 1.874436 | 1.4418 |
| DNASE1 | 0.526438 | 0.425836 | 2.917312 | 1.440369 |
| PROM2 | 0.526103 | 2.090485 | 1.625772 | 1.440034 |
| WDR72 | 0.525521 | 2.362812 | 1.324814 | 1.439453 |
| MMP7 | 0.525284 | 5.77453 | 1.313493 | 1.439217 |
| MYPN | 0.524355 | 1.256678 | 2.530674 | 1.438291 |
| ERP27 | 0.523875 | 1.515639 | 1.343862 | 1.437812 |
| MUC15 | 0.523842 | 0.263668 | 1.37899 | 1.437779 |
| NPSR1 | 0.522709 | 2.873967 | 1.095417 | 1.43665 |
| PRELID2 | 0.522644 | 1.31234 | 4.275343 | 1.436586 |
| SLC27A2 | 0.522602 | -0.29327 | 2.394135 | 1.436544 |
| TMEM38A | 0.521665 | 0.676497 | 3.402201 | 1.435611 |
| UGT1A6 | 0.521229 | 2.762482 | 1.4381 | 1.435177 |
| GCOM1 | 0.521216 | 2.217194 | 2.442924 | 1.435164 |
| GALNT4 | 0.519675 | 1.839358 | 2.774633 | 1.433632 |
| CBS | 0.519136 | -1.25795 | 2.360148 | 1.433097 |
| ESYT3 | 0.518772 | 0.410524 | 2.759975 | 1.432735 |
| PIWIL2 | 0.518207 | 1.364316 | 2.398206 | 1.432174 |
| LASS6 | 0.517921 | 0.337563 | 4.679157 | 1.43189 |
| KCNK1 | 0.517921 | 2.119438 | 3.100912 | 1.43189 |
| SFTPA2 | 0.517805 | 1.050245 | 1.79991 | 1.431775 |
| CDH1 | 0.517543 | 1.702941 | 4.07099 | 1.431515 |
| ZNF704 | 0.517427 | -0.08271 | 3.19934 | 1.4314 |
| PTCHD3 | 0.517307 | 1.077624 | 2.137747 | 1.431281 |
| NCOA2 | 0.51716 | -0.23234 | 2.53482 | 1.431135 |
| FGFRL1 | 0.516118 | 0.099958 | 3.15763 | 1.430102 |
| HSD17B6 | 0.515749 | -0.27218 | 2.518823 | 1.429736 |
| ARHGAP32 | 0.515668 | 0.687601 | 4.901191 | 1.429656 |
| AK3L1 | 0.515221 | -0.3353 | 2.632217 | 1.429213 |
| PRRG4 | 0.514356 | 0.431832 | 4.195988 | 1.428356 |
| THBS2 | 0.513816 | 2.717779 | 2.171463 | 1.427822 |
| LEFTY1 | 0.513366 | 3.942897 | 1.107183 | 1.427377 |
| MKI67 | 0.512792 | 0.224682 | 2.714958 | 1.426808 |
| CDHR5 | 0.512693 | 5.332438 | 1.626137 | 1.426711 |
| SDC4 | 0.512208 | 0.945418 | 3.576471 | 1.426231 |
| OTX1 | 0.51197 | 0.238855 | 2.761949 | 1.425996 |
| FXYD3 | 0.511651 | 3.853279 | 2.229567 | 1.425681 |

| | | | | |
|---|---|---|---|---|
| FKBP9L | 0.511151 | 1.263772 | 3.609065 | 1.425187 |
| KRTCAP3 | 0.511145 | 1.910005 | 2.095031 | 1.425181 |
| C1orf130 | 0.510987 | 3.445086 | 2.321412 | 1.425025 |
| FBXW12 | 0.51084 | 0.516651 | 2.436222 | 1.424879 |
| IFI27 | 0.510522 | 1.441327 | 2.236054 | 1.424566 |
| FGD6 | 0.509458 | 1.042417 | 3.442251 | 1.423515 |
| SLC12A2 | 0.50886 | 1.167389 | 3.338142 | 1.422925 |
| ERN2 | 0.508025 | 7.202153 | 1.263117 | 1.422102 |
| CCDC155 | 0.507918 | -0.15601 | 3.378809 | 1.421996 |
| ASPH | 0.50777 | 0.51244 | 3.904793 | 1.421851 |
| C12orf39 | 0.507026 | -1.15762 | 2.465302 | 1.421118 |
| NQO1 | 0.506776 | 1.666417 | 2.513626 | 1.420871 |
| LY6D | 0.506774 | 2.727017 | 0.947844 | 1.420869 |
| VTCN1 | 0.505884 | 2.449704 | 1.583931 | 1.419993 |
| TMTC2 | 0.505872 | 0.650643 | 4.143325 | 1.419982 |
| MYOF | 0.505299 | 1.195829 | 3.175234 | 1.419418 |
| ANO1 | 0.504201 | 2.573713 | 3.03791 | 1.418337 |
| ITGB4 | 0.50394 | 2.28089 | 2.026253 | 1.418081 |
| PPM1L | 0.502844 | 0.215836 | 3.442977 | 1.417005 |
| KRT7 | 0.502335 | 4.135697 | 1.512934 | 1.416504 |
| HSN2 | 0.502116 | 0.286903 | 2.688406 | 1.41629 |
| TRIM15 | 0.502 | 5.081244 | 1.569372 | 1.416175 |
| DPEP1 | 0.501991 | 4.465042 | 1.835469 | 1.416167 |
| ARHGAP42 | 0.501488 | 0.635178 | 3.383284 | 1.415673 |
| CCDC68 | 0.500177 | 2.288126 | 3.045233 | 1.414387 |
| PCDH1 | 0.499632 | 1.742793 | 4.354158 | 1.413853 |
| WNT7A | 0.499304 | 3.764683 | 1.247305 | 1.413531 |
| FAM190A | 0.499269 | 1.07273 | 3.507277 | 1.413497 |
| SLC5A1 | 0.498497 | 4.69153 | 1.780952 | 1.412741 |
| LOC731779 | 0.498411 | 1.104793 | 1.990208 | 1.412657 |
| SH3RF1 | 0.496994 | 0.965242 | 4.961522 | 1.41127 |
| TMPRSS15 | 0.496263 | 1.064783 | 1.224702 | 1.410555 |
| SLC30A1 | 0.496254 | 0.189724 | 5.036966 | 1.410547 |
| UMOD | 0.495735 | 0.304014 | 1.944895 | 1.410039 |
| ABCC3 | 0.495494 | 3.150209 | 2.06741 | 1.409803 |
| MUC13 | 0.494766 | 7.590866 | 1.606192 | 1.409092 |
| C16orf11 | 0.494562 | 0.000973 | 3.262627 | 1.408893 |
| STXBP6 | 0.494455 | 1.65046 | 2.614021 | 1.408788 |
| ATOH1 | 0.494295 | 0.760731 | 1.661901 | 1.408632 |
| PTGS2 | 0.494204 | 1.739444 | 1.791283 | 1.408544 |
| SOX11 | 0.494023 | 0.566791 | 1.770177 | 1.408367 |
| ALPI | 0.49325 | 1.357984 | 1.411549 | 1.407612 |
| LOC100271831 | 0.49296 | 1.341031 | 1.782405 | 1.40733 |
| VIL1 | 0.492189 | 6.33512 | 1.616831 | 1.406578 |
| PGA5 | 0.492093 | 0.206567 | 1.606193 | 1.406484 |
| DUOXA2 | 0.491805 | 5.542204 | 1.14773 | 1.406203 |

| | | | | |
|---|---|---|---|---|
| SDCBP2 | 0.491501 | 3.314081 | 2.376717 | 1.405907 |
| GPR110 | 0.491186 | 3.774366 | 1.429172 | 1.4056 |
| ALDH1L2 | 0.490836 | 1.31863 | 2.451127 | 1.405259 |
| FAM63B | 0.490163 | 0.26157 | 3.270752 | 1.404603 |
| NMUR2 | 0.4897 | 3.124249 | 1.206442 | 1.404153 |
| LOC100188947 | 0.489119 | 0.041352 | 3.067251 | 1.403587 |
| BMPR1B | 0.488927 | 0.318963 | 2.316128 | 1.403401 |
| GBP3 | 0.488519 | 0.926371 | 2.250561 | 1.403004 |
| C6orf132 | 0.488473 | 1.536757 | 2.394376 | 1.402959 |
| SAMD9 | 0.488462 | 1.257499 | 3.144449 | 1.402948 |
| GALNT6 | 0.487587 | 0.984441 | 2.426247 | 1.402098 |
| CASC5 | 0.487305 | -0.0768 | 2.466848 | 1.401824 |
| ANKFN1 | 0.485847 | 1.602069 | 1.907305 | 1.400408 |
| C6orf223 | 0.485419 | 3.119391 | 1.745185 | 1.399992 |
| ABO | 0.484897 | 2.036112 | 1.658088 | 1.399486 |
| EDIL3 | 0.484806 | 1.818922 | 1.901347 | 1.399397 |
| PMAIP1 | 0.483856 | 0.163231 | 3.255383 | 1.398476 |
| FOXL1 | 0.483628 | 4.043702 | 1.971528 | 1.398256 |
| NCCRP1 | 0.482819 | 0.404165 | 1.362839 | 1.397472 |
| AQP2 | 0.480941 | 1.680956 | 1.773134 | 1.395654 |
| TMEM144 | 0.480163 | 0.380451 | 3.525844 | 1.394901 |
| RHCG | 0.479943 | -0.49426 | 1.567666 | 1.394689 |
| SMPDL3B | 0.479934 | 1.478726 | 2.809226 | 1.39468 |
| CKAP2L | 0.479694 | -0.26632 | 2.699556 | 1.394448 |
| CCDC109A | 0.479313 | 1.173404 | 4.120098 | 1.39408 |
| IL1RN | 0.477967 | 2.15998 | 1.719233 | 1.39278 |
| FAM114A1 | 0.477521 | 0.700905 | 3.993785 | 1.392349 |
| INHA | 0.47752 | 1.176434 | 1.902035 | 1.392348 |
| SLC44A4 | 0.477452 | 4.576925 | 1.973932 | 1.392282 |
| RAET1L | 0.477299 | 1.780944 | 1.688032 | 1.392135 |
| KLHDC7A | 0.47688 | 1.742612 | 1.964692 | 1.39173 |
| RPS27 | 0.476833 | 0.484538 | 1.762445 | 1.391685 |
| AMY2B | 0.476724 | 2.551266 | 1.072504 | 1.39158 |
| BARX2 | 0.47635 | 2.409965 | 1.878997 | 1.39122 |
| PYCR1 | 0.475961 | 0.408227 | 3.430312 | 1.390844 |
| P2RY2 | 0.47588 | 0.662809 | 2.210296 | 1.390766 |
| CTSL2 | 0.475641 | 0.310423 | 2.258734 | 1.390536 |
| PHGR1 | 0.47545 | 3.612175 | 1.102146 | 1.390351 |
| EXOC6B | 0.47437 | -0.09772 | 3.083079 | 1.389311 |
| CEACAM20 | 0.474258 | 0.397854 | 2.016047 | 1.389203 |
| OAS1 | 0.474084 | 0.707167 | 2.746088 | 1.389036 |
| HABP2 | 0.474041 | 4.873295 | 1.229722 | 1.388995 |
| LEPROT | 0.473788 | 0.297958 | 3.33419 | 1.388751 |
| SRD5A3 | 0.472858 | 0.983281 | 3.742664 | 1.387856 |
| EPCAM | 0.472817 | 3.001685 | 3.45131 | 1.387817 |
| BSPRY | 0.472407 | 1.363828 | 3.53322 | 1.387422 |

| | | | | |
|---|---|---|---|---|
| SERTAD4 | 0.472215 | 0.061978 | 2.52362 | 1.387238 |
| C9orf129 | 0.472207 | 0.263375 | 3.072584 | 1.38723 |
| DOCK5 | 0.472006 | 1.091175 | 2.830578 | 1.387037 |
| YIPF6 | 0.471176 | -0.01157 | 5.960836 | 1.386239 |
| BRIP1 | 0.471083 | -0.76251 | 2.702446 | 1.38615 |
| LMTK2 | 0.47105 | 0.308221 | 3.148771 | 1.386118 |
| LAD1 | 0.470368 | 3.553586 | 2.230847 | 1.385463 |
| CYP3A7 | 0.470094 | 1.281962 | 2.356839 | 1.385199 |
| HS3ST1 | 0.469735 | 2.293309 | 2.270929 | 1.384855 |
| GRHL1 | 0.469295 | 0.094505 | 2.856851 | 1.384433 |
| TMBIM1 | 0.468904 | 1.306387 | 4.508646 | 1.384058 |
| SLC44A1 | 0.468493 | 0.512266 | 5.099967 | 1.383663 |
| KIF14 | 0.468339 | -0.13244 | 2.332177 | 1.383515 |
| SLC4A4 | 0.468156 | 3.329763 | 1.334491 | 1.38334 |
| BACH1 | 0.468147 | 0.223158 | 4.78456 | 1.383332 |
| SLK | 0.467614 | 0.664269 | 4.786411 | 1.382821 |
| HPSE | 0.466879 | 0.804909 | 3.631763 | 1.382116 |
| PDZD8 | 0.46685 | 0.82725 | 3.876278 | 1.382089 |
| CENPI | 0.466587 | -0.52963 | 2.641307 | 1.381837 |
| ATE1 | 0.466355 | -0.04496 | 3.738965 | 1.381614 |
| RAB11FIP1 | 0.46629 | 1.481863 | 4.041309 | 1.381552 |
| RRM2 | 0.465747 | -0.13458 | 2.550341 | 1.381032 |
| RCC1 | 0.464837 | -0.27004 | 2.754164 | 1.380162 |
| SLC35A3 | 0.464597 | 0.160703 | 4.378525 | 1.379932 |
| ANKS4B | 0.464572 | 4.906031 | 1.66949 | 1.379908 |
| SLC16A1 | 0.463856 | -0.20381 | 3.194625 | 1.379223 |
| ILDR1 | 0.46356 | 1.691628 | 3.109349 | 1.378941 |
| RBM47 | 0.463492 | 0.701533 | 4.480267 | 1.378875 |
| FRK | 0.463086 | 1.10827 | 3.367739 | 1.378488 |
| ABHD2 | 0.463071 | 1.363581 | 3.624588 | 1.378473 |
| TMPPE | 0.462166 | 0.149197 | 4.210951 | 1.377609 |
| E2F8 | 0.462007 | 0.096617 | 2.337085 | 1.377457 |
| EPO | 0.461736 | -0.1804 | 2.028098 | 1.377198 |
| CYP3A4 | 0.461606 | 1.577495 | 1.347744 | 1.377074 |
| TOP2A | 0.461248 | -0.22087 | 2.491178 | 1.376732 |
| HS3ST5 | 0.460811 | 1.055534 | 1.895189 | 1.376315 |
| CD9 | 0.460583 | -0.32119 | 3.417741 | 1.376098 |
| TMEM159 | 0.46055 | 0.560329 | 3.995575 | 1.376066 |
| PRSS16 | 0.459406 | 1.335707 | 2.618901 | 1.374976 |
| HMMR | 0.45919 | -0.04422 | 2.722579 | 1.37477 |
| ANXA2P3 | 0.458643 | 0.605824 | 2.698176 | 1.374249 |
| ESCO2 | 0.458577 | -0.15968 | 2.77579 | 1.374185 |
| OLFM4 | 0.458366 | 9.081458 | 0.846814 | 1.373985 |
| FABP2 | 0.458304 | 1.534283 | 1.163114 | 1.373926 |
| CCNT1 | 0.45813 | -0.17099 | 2.397434 | 1.37376 |
| SERPINB2 | 0.458049 | 3.627075 | 1.284116 | 1.373683 |

| | | | | |
|---|---|---|---|---|
| LMO7 | 0.457813 | 1.943687 | 3.498819 | 1.373459 |
| C12orf56 | 0.457733 | -0.68941 | 1.87872 | 1.373382 |
| FLNB | 0.457671 | 1.010022 | 3.128887 | 1.373323 |
| LYPD2 | 0.45764 | 2.111267 | 0.989685 | 1.373293 |
| IPMK | 0.457594 | -0.14988 | 2.651983 | 1.373249 |
| GALE | 0.457544 | 0.771706 | 3.018784 | 1.373202 |
| POTEE | 0.457407 | -0.34049 | 3.238993 | 1.373072 |
| NEBL | 0.456863 | 0.462934 | 3.362097 | 1.372554 |
| CLCN3 | 0.45682 | 0.295578 | 4.330573 | 1.372513 |
| CDC25C | 0.456807 | -0.15511 | 2.322461 | 1.372501 |
| SOX9 | 0.455947 | 1.552771 | 3.015876 | 1.371683 |
| MYLK2 | 0.455617 | 0.861734 | 1.526212 | 1.37137 |
| CCNI2 | 0.455408 | 1.766696 | 2.632901 | 1.37117 |
| FAM111B | 0.455273 | -0.41766 | 2.786013 | 1.371042 |
| MYO1E | 0.454918 | 1.119771 | 4.052685 | 1.370705 |
| FRRS1 | 0.454868 | 0.475374 | 2.08538 | 1.370658 |
| BMP4 | 0.454437 | 2.273564 | 2.241141 | 1.370248 |
| SLC16A7 | 0.454393 | 1.530966 | 2.176662 | 1.370206 |
| NHLRC2 | 0.454198 | 0.042246 | 3.810348 | 1.370021 |
| CD109 | 0.454099 | 0.475169 | 2.175926 | 1.369927 |
| INSL4 | 0.454055 | 0.139216 | 2.751246 | 1.369885 |
| PLEKHG6 | 0.453863 | 2.381015 | 2.953383 | 1.369703 |
| SPP1 | 0.453574 | 2.014731 | 1.640549 | 1.369428 |
| EIF2AK2 | 0.453437 | 0.042043 | 4.009996 | 1.369298 |
| A2ML1 | 0.453178 | 0.28266 | 0.847322 | 1.369053 |
| UGT1A9 | 0.45291 | 2.220075 | 1.163775 | 1.368799 |
| PLA2R1 | 0.452546 | 1.082812 | 2.545137 | 1.368453 |
| SGPP2 | 0.452258 | 2.110333 | 2.62693 | 1.36818 |
| PPARG | 0.451976 | 1.917385 | 2.168494 | 1.367912 |
| KCNN4 | 0.451802 | 2.940806 | 1.535672 | 1.367747 |
| CLSPN | 0.451654 | -0.59648 | 2.237714 | 1.367607 |
| IL1RAP | 0.45162 | 0.573119 | 2.632825 | 1.367575 |
| PCDHGA10 | 0.451557 | 0.076376 | 1.914948 | 1.367516 |
| NPTX1 | 0.451453 | 3.284599 | 1.730737 | 1.367417 |
| REEP3 | 0.450845 | 0.471797 | 4.541107 | 1.366841 |
| CLOCK | 0.450761 | 0.135227 | 3.268696 | 1.366761 |
| DIAPH2 | 0.449944 | 0.518908 | 4.813196 | 1.365987 |
| WNT5A | 0.449896 | 0.969127 | 2.267021 | 1.365942 |
| REL | 0.449706 | 0.204558 | 2.712297 | 1.365762 |
| SHANK2 | 0.449129 | 0.615384 | 3.104963 | 1.365216 |
| FAM196B | 0.447991 | -0.31072 | 2.280087 | 1.364139 |
| CD2AP | 0.447488 | 0.68943 | 4.51769 | 1.363664 |
| CPA4 | 0.4474 | 0.353399 | 1.725473 | 1.363581 |
| TDRD1 | 0.445579 | 0.850581 | 1.585156 | 1.361861 |
| CCL20 | 0.445538 | 2.868722 | 1.331389 | 1.361822 |
| GPR109A | 0.444995 | -0.34885 | 1.600008 | 1.36131 |

| | | | | |
|---|---|---|---|---|
| SLC16A10 | 0.444921 | 0.907595 | 2.384149 | 1.361239 |
| TOB1 | 0.444723 | 0.622403 | 3.840682 | 1.361052 |
| LOC284441 | 0.44439 | -0.05681 | 3.144275 | 1.360739 |
| LY75 | 0.444236 | 1.67504 | 2.257181 | 1.360593 |
| LYZ | 0.44413 | 3.333715 | 1.503094 | 1.360493 |
| SCN5A | 0.443673 | 0.517591 | 2.153613 | 1.360062 |
| FAM55B | 0.442841 | 0.402871 | 2.265887 | 1.359279 |
| FZD5 | 0.442773 | 0.874353 | 3.631822 | 1.359214 |
| SPAG4 | 0.442752 | 1.005628 | 2.633904 | 1.359194 |
| PAWR | 0.44275 | 0.357987 | 4.860965 | 1.359192 |
| CRCT1 | 0.441933 | 1.588661 | 1.298722 | 1.358423 |
| GPC4 | 0.441878 | 0.719659 | 3.327617 | 1.358372 |
| LDLR | 0.441692 | 1.908699 | 2.611755 | 1.358196 |
| KIF18A | 0.441506 | -0.54437 | 2.452195 | 1.358021 |
| PPP2R2C | 0.441029 | 1.226857 | 1.48123 | 1.357573 |
| ANTXR2 | 0.440612 | 1.695038 | 3.06048 | 1.35718 |
| TNFRSF11A | 0.44051 | 2.251925 | 2.352921 | 1.357084 |
| ZNF702P | 0.439393 | 0.109551 | 2.21147 | 1.356034 |
| SMAGP | 0.438917 | 1.524158 | 2.902227 | 1.355586 |
| PPY | 0.438769 | 7.96182 | 0.645799 | 1.355448 |
| C5orf46 | 0.438729 | 2.858367 | 1.560809 | 1.35541 |
| FBP2 | 0.438667 | 1.280782 | 1.521808 | 1.355351 |
| MMEL1 | 0.438609 | 2.043242 | 1.807916 | 1.355297 |
| HECW1 | 0.438232 | 0.950251 | 1.707341 | 1.354943 |
| DEPDC1 | 0.437866 | -0.4974 | 2.314862 | 1.354599 |
| OAS3 | 0.437832 | 0.226607 | 2.914291 | 1.354567 |
| UGCG | 0.437772 | 0.419098 | 2.832697 | 1.354511 |
| LIMS1 | 0.437368 | 0.347823 | 2.818963 | 1.354131 |
| IQGAP2 | 0.437228 | 1.123002 | 2.494407 | 1.354 |
| C1orf116 | 0.436461 | 3.28464 | 2.689352 | 1.353281 |
| P4HA1 | 0.436412 | 0.128346 | 3.655316 | 1.353235 |
| ECM1 | 0.436339 | 1.479901 | 2.159067 | 1.353166 |
| ENTPD8 | 0.436196 | 2.911365 | 1.632133 | 1.353032 |
| SEMG1 | 0.436039 | 1.294766 | 1.802379 | 1.352885 |
| AKR1B10 | 0.435918 | 3.991404 | 1.039569 | 1.352771 |
| RIF1 | 0.43568 | -0.01346 | 3.539184 | 1.352548 |
| PLOD2 | 0.435679 | 0.164538 | 2.634941 | 1.352548 |
| MYOM3 | 0.435564 | 2.35061 | 1.820022 | 1.35244 |
| C10orf118 | 0.435332 | 0.481933 | 3.731214 | 1.352222 |
| PBK | 0.435282 | -0.4821 | 2.542252 | 1.352175 |
| HIST1H2BC | 0.434942 | 0.0757 | 2.259595 | 1.351856 |
| PCSK9 | 0.434811 | 2.940431 | 1.693722 | 1.351733 |
| EVPL | 0.434787 | 1.68783 | 1.583339 | 1.351711 |
| SEC14L4 | 0.434031 | 1.646566 | 1.783691 | 1.351003 |
| DPP10 | 0.433856 | 0.524949 | 1.330762 | 1.350839 |
| TPX2 | 0.433713 | -0.33053 | 2.333873 | 1.350705 |

| | | | | |
|---|---|---|---|---|
| CXCR1 | 0.432808 | 1.158241 | 1.637316 | 1.349858 |
| PTGES | 0.432631 | 1.459715 | 1.667862 | 1.349693 |
| EPR1 | 0.432406 | -0.26478 | 2.125426 | 1.349482 |
| NRG4 | 0.432339 | 1.070182 | 1.625578 | 1.349419 |
| MAN2A1 | 0.432138 | 0.405638 | 2.846442 | 1.349231 |
| C8orf12 | 0.432088 | 0.032232 | 2.346807 | 1.349185 |
| STK31 | 0.431979 | 1.856821 | 1.598244 | 1.349083 |
| SLC5A3 | 0.431667 | -0.82495 | 3.738585 | 1.348791 |
| NR4A2 | 0.431595 | 0.126288 | 2.272321 | 1.348724 |
| TMEM125 | 0.431449 | 1.91605 | 2.895605 | 1.348587 |
| CST4 | 0.431043 | 3.249536 | 1.447127 | 1.348208 |
| SH2D3A | 0.430423 | 2.097025 | 1.750132 | 1.347628 |
| DSG4 | 0.430287 | 0.266195 | 2.808325 | 1.347501 |
| CBLC | 0.429588 | 3.220658 | 2.147797 | 1.346849 |
| FGL1 | 0.429372 | 3.218614 | 0.914917 | 1.346647 |
| CLDN7 | 0.429313 | 2.049049 | 2.941926 | 1.346592 |
| C4BPA | 0.429253 | 3.897109 | 1.054871 | 1.346536 |
| C6orf150 | 0.428519 | 0.694411 | 3.107523 | 1.345852 |
| BATF2 | 0.428215 | 0.791924 | 3.067849 | 1.345568 |
| CLDN23 | 0.428019 | 1.897883 | 2.088529 | 1.345385 |
| TOX3 | 0.427978 | 2.946714 | 1.987013 | 1.345347 |
| KRT23 | 0.427853 | 3.596988 | 1.268622 | 1.34523 |
| VNN1 | 0.427795 | 3.770656 | 1.404856 | 1.345176 |
| FGG | 0.427391 | 3.666825 | 0.904155 | 1.3448 |
| FOXQ1 | 0.427259 | 3.34476 | 1.873538 | 1.344677 |
| LNPEP | 0.426776 | 0.150209 | 3.254122 | 1.344226 |
| RETNLB | 0.426759 | 0.074588 | 2.190903 | 1.344211 |
| FNIP2 | 0.426721 | 0.489282 | 2.773895 | 1.344175 |
| PKP2 | 0.42659 | 1.844626 | 2.669006 | 1.344053 |
| AP1S3 | 0.426412 | 1.634503 | 2.824975 | 1.343887 |
| MAB21L2 | 0.426018 | 3.051809 | 1.231671 | 1.34352 |
| BUB1 | 0.425926 | -0.22826 | 2.352325 | 1.343434 |
| GAS2L3 | 0.425918 | 0.016541 | 1.974765 | 1.343427 |
| IRAK2 | 0.425883 | 1.387386 | 2.750366 | 1.343394 |
| BACE2 | 0.425813 | 1.619845 | 2.814995 | 1.343329 |
| BAG4 | 0.425173 | -0.24585 | 3.93794 | 1.342734 |
| ITGA11 | 0.425152 | 2.339011 | 1.734577 | 1.342714 |
| LOC641298 | 0.424852 | -0.04178 | 1.82692 | 1.342435 |
| SYTL4 | 0.424441 | 1.094307 | 3.243889 | 1.342052 |
| GPR115 | 0.423527 | 3.186686 | 1.693714 | 1.341202 |
| TCF7L2 | 0.423283 | 0.806367 | 3.653411 | 1.340975 |
| C4BPB | 0.42328 | 4.388281 | 1.393234 | 1.340973 |
| PTAFR | 0.42323 | 1.206148 | 2.23543 | 1.340926 |
| BCMO1 | 0.423091 | 2.290428 | 2.151778 | 1.340797 |
| NTN4 | 0.422795 | 1.021779 | 2.910152 | 1.340522 |
| FN1 | 0.422749 | 1.409428 | 1.737176 | 1.340479 |

| | | | | |
|---|---|---|---|---|
| ASPM | 0.422548 | -0.03083 | 2.108612 | 1.340292 |
| KIF20A | 0.422444 | -0.2833 | 2.209205 | 1.340196 |
| KRT18 | 0.421216 | 2.191715 | 2.823525 | 1.339056 |
| TMEM139 | 0.42027 | 2.033879 | 1.962364 | 1.338178 |
| HIST1H2BJ | 0.420131 | -0.14714 | 2.012198 | 1.338049 |
| C20orf151 | 0.420071 | 1.172312 | 1.699874 | 1.337993 |
| MBOAT2 | 0.419505 | 0.723641 | 2.950601 | 1.337468 |
| C1orf58 | 0.419163 | -0.0116 | 3.486561 | 1.337152 |
| E2F7 | 0.419145 | -0.30872 | 2.096784 | 1.337135 |
| PARD3 | 0.418951 | 0.095414 | 5.365957 | 1.336955 |
| EXPH5 | 0.418891 | 0.585871 | 3.551021 | 1.336899 |
| FLJ23867 | 0.418817 | 2.038423 | 2.037916 | 1.336831 |
| ASAM | 0.418698 | 1.622913 | 1.554973 | 1.336721 |
| KRT80 | 0.418477 | 2.068939 | 1.690252 | 1.336516 |
| CLDN10 | 0.418416 | 4.817734 | 0.985902 | 1.33646 |
| PPP1R3B | 0.418198 | 0.064296 | 3.827566 | 1.336258 |
| SHROOM3 | 0.418172 | 2.197081 | 3.483899 | 1.336233 |
| BARD1 | 0.417723 | -0.21564 | 2.259614 | 1.335818 |
| DIAPH3 | 0.416984 | -0.11538 | 2.399273 | 1.335133 |
| TSPAN15 | 0.416601 | 1.644594 | 2.773331 | 1.334779 |
| CHRNA5 | 0.416597 | 1.352062 | 2.394859 | 1.334775 |
| PRSS22 | 0.416253 | 3.790637 | 1.7657 | 1.334457 |
| TMEM30B | 0.415762 | 1.965909 | 3.408434 | 1.334003 |
| GSTT1 | 0.415713 | 0.683348 | 0.800756 | 1.333958 |
| C15orf38 | 0.415554 | 0.12167 | 4.877177 | 1.333811 |
| LOXL2 | 0.414442 | 1.304646 | 2.160281 | 1.332783 |
| IDE | 0.413981 | -0.02364 | 4.009394 | 1.332357 |
| AMIGO2 | 0.413941 | 1.774992 | 2.278347 | 1.332321 |
| COBL | 0.413648 | 1.0249 | 2.985651 | 1.33205 |
| GPR160 | 0.413519 | 0.518035 | 2.873001 | 1.331931 |
| SPACA3 | 0.413325 | 0.922373 | 1.637673 | 1.331752 |
| ANXA2P1 | 0.41301 | 1.364924 | 2.582387 | 1.331461 |
| HNF1B | 0.412958 | 5.074307 | 1.488764 | 1.331413 |
| GDNF | 0.412626 | -0.00467 | 2.733256 | 1.331107 |
| KLHL23 | 0.412231 | 0.216035 | 2.351889 | 1.330742 |
| ST14 | 0.411552 | 2.006827 | 2.869284 | 1.330116 |
| NCEH1 | 0.411479 | 0.705779 | 3.308848 | 1.330049 |
| ARL6IP1 | 0.411442 | 0.119115 | 4.565281 | 1.330014 |
| IGFBP1 | 0.411222 | 2.568303 | 1.181513 | 1.329812 |
| SERPINA4 | 0.411103 | 7.151679 | 1.173002 | 1.329702 |
| AHNAK2 | 0.411074 | 1.888905 | 1.452232 | 1.329675 |
| ?\|652919 | 0.409973 | 0.179548 | 1.202296 | 1.328661 |
| NAALADL2 | 0.409845 | 1.067705 | 2.728857 | 1.328543 |
| ALB | 0.40944 | 2.730791 | 0.821896 | 1.32817 |
| LOC100124692 | 0.409015 | 4.307025 | 1.078182 | 1.327779 |
| UEVLD | 0.408925 | 0.057104 | 4.486642 | 1.327696 |

| | | | | |
|---|---|---|---|---|
| PLEKHA7 | 0.408502 | 1.334576 | 2.943139 | 1.327307 |
| MME | 0.408471 | -0.27816 | 1.551322 | 1.327278 |
| CDA | 0.408335 | 1.858416 | 1.639893 | 1.327153 |
| ROR1 | 0.408302 | 1.640763 | 2.096946 | 1.327123 |
| SPRR3 | 0.408241 | 3.114098 | 0.830879 | 1.327067 |
| UGT1A7 | 0.408161 | -0.35474 | 1.805214 | 1.326994 |
| SLC41A2 | 0.407995 | 1.468717 | 2.864435 | 1.32684 |
| BAIAP2L1 | 0.407818 | 1.918711 | 2.825984 | 1.326678 |
| ELOVL6 | 0.407763 | 0.363729 | 3.208627 | 1.326628 |
| KALRN | 0.407579 | 1.126411 | 3.001365 | 1.326458 |
| PRB2 | 0.407185 | 0.809622 | 1.603377 | 1.326096 |
| ANXA4 | 0.407162 | 2.338795 | 2.285532 | 1.326074 |
| EPHB6 | 0.407101 | 1.501762 | 1.819301 | 1.326018 |
| TMPRSS3 | 0.406877 | 4.151331 | 1.558453 | 1.325812 |
| FAM83G | 0.406767 | 1.095156 | 3.029655 | 1.325711 |
| CEACAM18 | 0.406714 | 1.369361 | 1.440665 | 1.325663 |
| CCRL1 | 0.406511 | 1.911025 | 2.084925 | 1.325476 |
| EPHX4 | 0.406501 | 1.381563 | 1.907973 | 1.325467 |
| SEMA4B | 0.406237 | 0.764713 | 3.050799 | 1.325224 |
| PRKG1 | 0.406213 | 1.383562 | 1.842054 | 1.325203 |
| VILL | 0.40616 | 3.342886 | 1.64965 | 1.325154 |
| CDCA7 | 0.405938 | 0.913553 | 1.513292 | 1.32495 |
| KIF23 | 0.405449 | -0.2341 | 2.395697 | 1.324501 |
| ZNF217 | 0.405338 | -0.13854 | 3.503843 | 1.324399 |
| SCUBE3 | 0.405274 | 0.6903 | 1.457186 | 1.32434 |
| GULP1 | 0.405047 | 1.428751 | 2.87672 | 1.324132 |
| STK38L | 0.405019 | 0.883282 | 4.215662 | 1.324106 |
| PLA2G4A | 0.404919 | 1.345319 | 2.517408 | 1.324015 |
| MTFR1 | 0.404703 | -0.27304 | 5.670109 | 1.323816 |
| GPR128 | 0.404445 | 2.598253 | 1.080911 | 1.32358 |
| RAB3D | 0.404135 | -0.14919 | 3.951974 | 1.323295 |
| SULF1 | 0.403992 | 2.615025 | 1.668268 | 1.323164 |
| KIAA1211 | 0.403788 | 2.38051 | 2.370809 | 1.322977 |
| ANO6 | 0.403279 | 0.185655 | 3.627921 | 1.32251 |
| C10orf12 | 0.403114 | 0.204153 | 3.866664 | 1.322359 |
| SULT2B1 | 0.402303 | 1.408209 | 1.481796 | 1.321616 |
| HAVCR1 | 0.402295 | 0.996099 | 1.309291 | 1.321609 |
| ELF3 | 0.402081 | 3.443389 | 2.053575 | 1.321412 |
| CDS1 | 0.401403 | 1.009695 | 3.478204 | 1.320792 |
| PAIP2B | 0.401394 | -0.75251 | 1.860892 | 1.320783 |
| TAF1L | 0.401298 | -0.16873 | 3.131347 | 1.320696 |
| MELK | 0.40116 | -0.05986 | 1.912869 | 1.320569 |
| FAM13A | 0.400985 | 0.270136 | 3.325868 | 1.320409 |
| NGEF | 0.400916 | 1.55267 | 1.716675 | 1.320346 |
| SYTL2 | 0.400304 | 1.281015 | 2.440079 | 1.319786 |
| EIF2C2 | 0.399972 | 0.168466 | 3.721712 | 1.319482 |

| | | | | |
|---|---|---|---|---|
| APOOL | 0.399887 | -0.42952 | 3.135391 | 1.319405 |
| RC3H2 | 0.399786 | -0.06312 | 3.570703 | 1.319312 |
| GRB7 | 0.399652 | 2.011167 | 2.53177 | 1.31919 |
| TSPAN5 | 0.399483 | 0.161331 | 2.529898 | 1.319035 |
| KCNE3 | 0.39893 | 1.838982 | 1.846071 | 1.31853 |
| ASAP2 | 0.398489 | 0.96649 | 3.443914 | 1.318127 |
| PANK3 | 0.398173 | 0.039621 | 4.095219 | 1.317838 |
| ANTXR1 | 0.398164 | 1.405677 | 2.005198 | 1.31783 |
| ERN1 | 0.398149 | 0.571671 | 2.076846 | 1.317816 |
| C4orf36 | 0.398047 | -0.05591 | 3.053059 | 1.317723 |
| MIPOL1 | 0.397879 | 0.391962 | 3.691198 | 1.317569 |
| PPTC7 | 0.397075 | 0.038075 | 2.780577 | 1.316835 |
| MARVELD3 | 0.396848 | 1.571982 | 3.389197 | 1.316628 |
| TRIM40 | 0.396766 | 1.229421 | 1.632541 | 1.316553 |
| PRRG1 | 0.396365 | 0.795362 | 3.476915 | 1.316187 |
| SPOCK1 | 0.396054 | 0.925558 | 1.809174 | 1.315904 |
| KIAA1522 | 0.395922 | 0.684624 | 3.254072 | 1.315783 |
| CXCR2 | 0.395804 | 0.483821 | 1.479972 | 1.315676 |
| BAGE2 | 0.395657 | -0.26413 | 2.902602 | 1.315542 |
| DNMBP | 0.395594 | 0.88439 | 3.857769 | 1.315484 |
| AZIN1 | 0.395472 | -0.07031 | 6.152577 | 1.315373 |
| ABTB2 | 0.395236 | 0.736216 | 2.459927 | 1.315158 |
| EPS8 | 0.395112 | 0.989672 | 3.082037 | 1.315045 |
| KRT6C | 0.395103 | 1.471397 | 0.994665 | 1.315037 |
| TGIF1 | 0.394916 | 0.132983 | 2.923011 | 1.314866 |
| SLC39A8 | 0.394614 | -0.36978 | 3.091227 | 1.314591 |
| C1QL3 | 0.39458 | -0.18102 | 1.794586 | 1.31456 |
| MYO5C | 0.39391 | 1.346467 | 3.134488 | 1.313949 |
| PCDH7 | 0.393608 | 1.653881 | 1.782094 | 1.313675 |
| SLC1A5 | 0.393445 | 0.240151 | 2.9892 | 1.313526 |
| PYGB | 0.393125 | 0.490551 | 3.215904 | 1.313235 |
| GSDMB | 0.392446 | 2.543922 | 1.762194 | 1.312617 |
| PPAPDC1A | 0.39243 | 2.52755 | 1.368095 | 1.312602 |
| ENTHD1 | 0.392331 | 1.213472 | 1.543029 | 1.312512 |
| C5orf23 | 0.39219 | 1.393907 | 1.682333 | 1.312384 |
| MYH16 | 0.391801 | 2.48973 | 1.319125 | 1.31203 |
| CHST4 | 0.391676 | 6.004372 | 1.16035 | 1.311917 |
| ODAM | 0.390676 | 4.014442 | 1.083074 | 1.311008 |
| MATN4 | 0.390261 | 0.570795 | 1.661144 | 1.310631 |
| F11R | 0.390236 | 0.764032 | 3.514339 | 1.310607 |
| DNAH2 | 0.390087 | 2.422751 | 1.525794 | 1.310473 |
| C1orf210 | 0.389939 | 2.083703 | 2.577151 | 1.310338 |
| MTNR1A | 0.389939 | 0.968685 | 1.899827 | 1.310338 |
| DQX1 | 0.389678 | 1.826103 | 1.024382 | 1.310101 |
| SUCNR1 | 0.389364 | 0.854051 | 1.37059 | 1.309816 |
| LRRC66 | 0.389271 | 3.53296 | 1.52437 | 1.309731 |

| | | | | |
|---|---|---|---|---|
| CHDH | 0.388346 | 1.192914 | 2.77791 | 1.308892 |
| CORO2A | 0.388203 | 1.552676 | 2.14629 | 1.308762 |
| SLC35F5 | 0.38791 | 0.089027 | 5.246326 | 1.308496 |
| EPHA7 | 0.387146 | -0.35689 | 1.208419 | 1.307803 |
| NLN | 0.387059 | -0.43822 | 3.532119 | 1.307725 |
| ETV4 | 0.386934 | 1.610399 | 2.176716 | 1.307611 |
| TREM1 | 0.386548 | 1.783965 | 1.459808 | 1.307262 |
| CTNND1 | 0.386546 | 0.394492 | 4.703836 | 1.30726 |
| HIST1H2AG | 0.386477 | -0.63722 | 2.394299 | 1.307197 |
| HOXA11AS | 0.386032 | -0.48769 | 1.250584 | 1.306794 |
| IGFL3 | 0.386003 | 1.14108 | 1.449154 | 1.306768 |
| SLC26A3 | 0.385493 | 0.394264 | 0.967062 | 1.306306 |
| CAPN5 | 0.385362 | 2.092468 | 2.076639 | 1.306188 |
| LDHA | 0.38536 | 0.077059 | 2.998534 | 1.306186 |
| KIAA1161 | 0.385198 | 0.755718 | 2.639599 | 1.306039 |
| HIST1H1C | 0.38494 | 0.121872 | 2.112475 | 1.305805 |
| YES1 | 0.3846 | 0.101741 | 5.342865 | 1.305498 |
| ANKRD36BP1 | 0.384582 | -0.10793 | 1.923493 | 1.305481 |
| ANG | 0.384374 | 1.570096 | 2.358001 | 1.305293 |
| TRIM44 | 0.384249 | -0.04574 | 3.846499 | 1.30518 |
| MMP8 | 0.384152 | 0.845237 | 1.514938 | 1.305093 |
| SPIN4 | 0.383658 | 0.18485 | 3.21499 | 1.304646 |
| CDH11 | 0.383222 | 1.833734 | 1.708971 | 1.304251 |
| ATP2C2 | 0.383146 | 2.285003 | 1.781702 | 1.304183 |
| MFSD9 | 0.383062 | 0.249491 | 4.705325 | 1.304107 |
| TTC22 | 0.382804 | 1.910479 | 2.679772 | 1.303874 |
| PLBD1 | 0.38247 | 2.044929 | 1.848995 | 1.303572 |
| ABCC13 | 0.382068 | -0.22352 | 2.20629 | 1.303209 |
| PAFAH1B2 | 0.382003 | 0.010061 | 3.234056 | 1.30315 |
| MAML2 | 0.381765 | 0.531612 | 2.525198 | 1.302935 |
| KIAA1244 | 0.380972 | 1.136268 | 1.917316 | 1.302219 |
| SMYD1 | 0.380917 | -0.32998 | 1.434102 | 1.302169 |
| PDE11A | 0.380253 | 2.038757 | 2.028344 | 1.30157 |
| NOX1 | 0.380201 | 1.380694 | 1.038375 | 1.301523 |
| CYP2J2 | 0.379441 | 0.545844 | 2.164336 | 1.300838 |
| C11orf90 | 0.379259 | 1.206221 | 2.119387 | 1.300674 |
| KRT19 | 0.379215 | 3.973703 | 1.392059 | 1.300634 |

**Table S3.** Top 1000 significantly down-regulated DEGs in high versus low-risk PDAC patients in TCGA dataset

| Gene symbol | logFC | AveExpr | t | Fold Change (FC) |
|---|---|---|---|---|
| AGAP6 | -0.82896 | 0.298031 | -7.28266 | 0.562933 |
| ZNF789 | -0.73484 | 0.185556 | -7.25867 | 0.600886 |
| CHKB-CPT1B | -0.97932 | 0.444076 | -6.95515 | 0.507217 |
| LOC100130557 | -0.79092 | -0.01894 | -6.87096 | 0.577976 |
| ?\|155060 | -0.74192 | 0.087002 | -6.88417 | 0.597945 |
| LOC100129387 | -0.65593 | -0.33968 | -6.90581 | 0.634664 |
| TDRD10 | -1.00335 | -0.1307 | -6.82133 | 0.498841 |
| ANKRD23 | -0.70175 | 0.175058 | -6.8109 | 0.614825 |
| KCNIP2 | -1.16445 | 0.069399 | -6.74413 | 0.446134 |
| FAM13AOS | -0.83475 | 0.693129 | -6.75041 | 0.560679 |
| LOC100128288 | -0.78869 | -0.08332 | -6.73324 | 0.578869 |
| TRPV1 | -0.74996 | -0.20077 | -6.73033 | 0.594619 |
| NPFF | -0.98305 | 0.636419 | -6.64283 | 0.505909 |
| MYH3 | -0.93248 | -0.05713 | -6.63969 | 0.523957 |
| CAPN3 | -0.92034 | -0.24453 | -6.54769 | 0.528384 |
| SSPO | -1.33802 | -0.23303 | -6.52886 | 0.395564 |
| REC8 | -1.1813 | 0.599561 | -6.51718 | 0.440953 |
| C17orf86 | -0.63963 | -0.22703 | -6.49203 | 0.641876 |
| ZNF767 | -0.62923 | 0.179294 | -6.49339 | 0.64652 |
| AGER | -0.95471 | -0.29094 | -6.44271 | 0.515946 |
| EGFL8 | -0.94211 | 0.482507 | -6.43974 | 0.52047 |
| LOC221442 | -0.97101 | 0.338736 | -6.38557 | 0.510149 |
| LOC338799 | -0.93198 | 0.158939 | -6.37134 | 0.524138 |
| C9orf45 | -0.91522 | -0.52864 | -6.39088 | 0.530264 |
| GABBR1 | -0.8246 | 0.414914 | -6.37382 | 0.564638 |
| LOC390595 | -0.82391 | -0.14762 | -6.4095 | 0.56491 |
| LOC91316 | -0.79584 | 0.194536 | -6.39629 | 0.576006 |
| FAM179A | -1.28586 | 1.202948 | -6.34731 | 0.410126 |
| CDK3 | -0.79248 | 0.156282 | -6.33814 | 0.577351 |
| LY6G5B | -0.77118 | 0.15492 | -6.31865 | 0.585938 |
| SGSM2 | -0.67129 | -0.1488 | -6.31526 | 0.627944 |
| IL11RA | -0.65432 | -0.19443 | -6.31477 | 0.635375 |
| HERC2P2 | -0.94287 | 0.221057 | -6.28724 | 0.520198 |
| PLGLB2 | -0.83256 | 0.016386 | -6.28559 | 0.56153 |
| NPPA | -0.8098 | -0.12176 | -6.27495 | 0.570461 |
| CELF6 | -1.04335 | 0.384394 | -6.27008 | 0.4852 |
| KIAA0895L | -0.66868 | 0.178569 | -6.26522 | 0.629082 |
| NCRNA00202 | -0.88218 | -0.48453 | -6.22672 | 0.542547 |
| SEC31B | -1.02138 | 0.244576 | -6.21517 | 0.492644 |
| TCTE3 | -0.64803 | 0.127002 | -6.21777 | 0.638152 |
| PPIEL | -0.93456 | -0.04495 | -6.19391 | 0.523202 |
| ACRC | -0.70467 | 0.193424 | -6.19046 | 0.613584 |
| UCP3 | -0.6953 | -0.30983 | -6.19271 | 0.617582 |

| | | | | |
|---|---|---|---|---|
| ZGLP1 | -0.91233 | -0.04383 | -6.18171 | 0.531325 |
| AGAP4 | -0.67352 | 0.381433 | -6.18156 | 0.626976 |
| TTLL3 | -0.74404 | 0.620898 | -6.16893 | 0.597066 |
| FAM186A | -0.78792 | 0.004904 | -6.14577 | 0.579178 |
| DFNB59 | -0.67241 | 0.225408 | -6.13527 | 0.627457 |
| CATSPER2 | -0.74384 | 0.430986 | -6.12411 | 0.59715 |
| PNMA3 | -1.37686 | -0.58005 | -6.07871 | 0.385056 |
| C3orf47 | -0.80324 | -0.12361 | -6.07697 | 0.57306 |
| PILRB | -0.85615 | 0.108331 | -6.0538 | 0.552426 |
| NCRNA00174 | -0.7598 | -0.13386 | -6.05093 | 0.590577 |
| LOC728392 | -1.16608 | 1.24698 | -6.03614 | 0.44563 |
| NFKBID | -0.81243 | 0.437924 | -6.03209 | 0.569423 |
| C6orf81 | -0.92714 | -0.32326 | -5.99973 | 0.5259 |
| LOC286367 | -0.7 | 0.306923 | -5.99236 | 0.615572 |
| LOC100132287 | -0.74537 | 0.200077 | -5.97021 | 0.596515 |
| COLQ | -0.72821 | 0.284038 | -5.9664 | 0.603654 |
| LOC168474 | -0.83265 | -0.49101 | -5.95969 | 0.561498 |
| EGFL7 | -0.86763 | 0.242738 | -5.92304 | 0.548045 |
| NPIPL3 | -0.87565 | 0.415212 | -5.8822 | 0.545007 |
| CROCCL2 | -0.64998 | 0.264692 | -5.8741 | 0.637291 |
| RLTPR | -1.28929 | 0.516076 | -5.86797 | 0.409153 |
| CYP2D7P1 | -1.07414 | 0.192393 | -5.8616 | 0.474955 |
| MTMR9L | -0.70447 | 0.93583 | -5.85496 | 0.613666 |
| GOLGA8A | -0.93225 | 0.354871 | -5.81267 | 0.52404 |
| KIAA1683 | -0.9016 | 0.004983 | -5.80431 | 0.535292 |
| PROCA1 | -0.63232 | 0.26235 | -5.78439 | 0.645138 |
| DNHD1 | -0.71984 | 0.077745 | -5.76504 | 0.607164 |
| ZMAT1 | -0.94556 | 0.20096 | -5.75267 | 0.519228 |
| CSAD | -0.73857 | -0.19516 | -5.75356 | 0.599331 |
| PRRT1 | -0.73185 | 0.076506 | -5.74567 | 0.602131 |
| LTB4R | -0.6892 | -0.04121 | -5.74299 | 0.620197 |
| ZCCHC18 | -1.03571 | 0.939406 | -5.72513 | 0.487775 |
| ATG16L2 | -0.68445 | 0.39021 | -5.72598 | 0.622242 |
| CRYGS | -0.81109 | 0.506134 | -5.6911 | 0.56995 |
| C19orf76 | -0.85901 | 0.497706 | -5.66727 | 0.551331 |
| CPT1B | -0.93174 | 0.288227 | -5.66007 | 0.524226 |
| WNT10B | -1.00895 | 0.198374 | -5.6494 | 0.496907 |
| ZNF540 | -0.87997 | 0.141268 | -5.64884 | 0.54338 |
| SNHG10 | -0.66304 | 0.112092 | -5.64692 | 0.631544 |
| ENO3 | -0.73739 | -0.80463 | -5.63817 | 0.599825 |
| TMEM151B | -0.87993 | -0.0227 | -5.62799 | 0.543395 |
| C7orf51 | -1.0675 | 1.457896 | -5.61418 | 0.477145 |
| SGK494 | -0.64735 | -0.30701 | -5.61416 | 0.638454 |
| CCR10 | -0.90416 | 0.184724 | -5.58995 | 0.534345 |
| KANK3 | -0.75536 | 0.220713 | -5.58897 | 0.592398 |
| MAPK11 | -0.7034 | 0.253619 | -5.5888 | 0.614124 |

| | | | | |
|---|---|---|---|---|
| RFPL3S | -0.74321 | 0.072657 | -5.58745 | 0.59741 |
| PGAM2 | -0.77087 | -0.51182 | -5.58052 | 0.586065 |
| KIAA1875 | -1.05131 | 0.068222 | -5.54887 | 0.48253 |
| LRRC37A3 | -0.71393 | -0.3328 | -5.53316 | 0.609656 |
| SH2D3C | -0.69606 | 0.129933 | -5.5333 | 0.617255 |
| C10orf68 | -0.69805 | 0.51434 | -5.52024 | 0.616404 |
| H2BFXP | -0.78065 | 0.53864 | -5.508 | 0.582105 |
| SLC25A18 | -0.7661 | -0.80586 | -5.50525 | 0.588005 |
| ADCY4 | -0.7012 | 1.093276 | -5.4539 | 0.615059 |
| DPY19L2 | -1.08944 | 0.14723 | -5.44441 | 0.469943 |
| LOC100133331 | -0.65827 | 0.190559 | -5.44356 | 0.633636 |
| LOC349114 | -0.68108 | 0.26264 | -5.42822 | 0.623696 |
| LPAL2 | -0.71128 | 0.149274 | -5.41393 | 0.610777 |
| ARHGAP33 | -0.6868 | -0.22453 | -5.40952 | 0.621231 |
| CHRNA10 | -0.69472 | 0.006875 | -5.40445 | 0.61783 |
| LCNL1 | -0.92375 | 0.566363 | -5.40138 | 0.527139 |
| GHRLOS | -0.6436 | 0.010211 | -5.3611 | 0.640114 |
| C16orf79 | -1.03091 | 0.261435 | -5.35363 | 0.489403 |
| C20orf160 | -0.70756 | 0.240226 | -5.35206 | 0.612356 |
| PABPC1L | -0.91789 | 0.304028 | -5.34715 | 0.529281 |
| SLC12A1 | -0.84771 | -0.76061 | -5.34729 | 0.555665 |
| AGAP7 | -0.77148 | -0.06153 | -5.3404 | 0.585815 |
| SCNN1D | -0.99253 | -0.02264 | -5.33048 | 0.502596 |
| LOC100129726 | -0.68827 | -0.03654 | -5.31419 | 0.620599 |
| A1BG | -0.79169 | -0.30465 | -5.30196 | 0.577666 |
| VENTX | -0.80795 | 0.934427 | -5.2853 | 0.571193 |
| L3MBTL | -0.73432 | 0.241636 | -5.27407 | 0.601101 |
| CCDC17 | -0.76524 | 0.066193 | -5.27309 | 0.588357 |
| ACCN3 | -1.0122 | 0.203273 | -5.26404 | 0.495789 |
| CYP21A2 | -1.04596 | 0.037575 | -5.2605 | 0.484323 |
| ZNF692 | -0.65679 | -0.11269 | -5.25174 | 0.63429 |
| FGF17 | -1.05251 | 0.226014 | -5.24962 | 0.482129 |
| PLAC9 | -0.97309 | 1.218229 | -5.24135 | 0.509413 |
| NTN5 | -0.77089 | -0.1068 | -5.24053 | 0.586055 |
| EID2B | -0.66131 | -0.03554 | -5.24085 | 0.632302 |
| C6orf163 | -0.68893 | -0.31049 | -5.23497 | 0.620314 |
| GOLGA8B | -0.98911 | 0.359257 | -5.22237 | 0.50379 |
| FBXO41 | -0.70056 | 0.307576 | -5.21805 | 0.615331 |
| SLC25A27 | -0.91321 | 0.300753 | -5.20842 | 0.531001 |
| C21orf58 | -0.85182 | 0.033213 | -5.20888 | 0.554085 |
| RELL2 | -0.65374 | 0.105157 | -5.19161 | 0.635632 |
| KLRA1 | -0.7708 | 0.328678 | -5.18873 | 0.586093 |
| LOC100216545 | -0.65223 | 0.081466 | -5.18407 | 0.636297 |
| NOVA1 | -1.21317 | -0.26208 | -5.17294 | 0.431321 |
| RAMP2 | -0.65258 | 0.290991 | -5.14088 | 0.63614 |
| SNHG3 | -0.63251 | 0.14486 | -5.13879 | 0.645051 |

| | | | | |
|---|---|---|---|---|
| FAM18A | -0.649 | 0.206388 | -5.13732 | 0.637721 |
| HFM1 | -1.00247 | -0.15457 | -5.13307 | 0.499144 |
| RASL10A | -0.71144 | -0.22605 | -5.13313 | 0.61071 |
| SOX18 | -0.76987 | 0.150212 | -5.13204 | 0.58647 |
| MEG3 | -1.05812 | 2.683388 | -5.12147 | 0.480259 |
| VN1R1 | -0.63385 | 0.075992 | -5.10645 | 0.644454 |
| SOX17 | -0.74993 | 0.198948 | -5.10015 | 0.594633 |
| GRASP | -0.66536 | 0.887768 | -5.09605 | 0.630533 |
| TMEM86B | -0.73539 | 0.535441 | -5.09205 | 0.600656 |
| NCRNA00107 | -0.73102 | 0.137444 | -5.08267 | 0.602479 |
| CPLX3 | -0.64213 | -0.93319 | -5.08122 | 0.640767 |
| HRC | -0.63588 | -0.10412 | -5.06599 | 0.643547 |
| C1QTNF4 | -1.03259 | 0.306061 | -5.06394 | 0.488831 |
| NEIL1 | -0.73945 | 0.551086 | -5.05606 | 0.598967 |
| MZF1 | -0.67645 | 0.213541 | -5.05633 | 0.625702 |
| HPX | -0.98827 | -0.298 | -5.05197 | 0.504082 |
| KCNAB3 | -0.75993 | 0.350168 | -5.05239 | 0.590524 |
| CNIH2 | -1.29615 | 0.727154 | -5.04905 | 0.407212 |
| ROBO3 | -0.67962 | 0.624719 | -5.04441 | 0.624331 |
| ASPDH | -1.33617 | 0.413998 | -5.04278 | 0.39607 |
| C6orf26 | -0.74934 | 0.643272 | -5.03737 | 0.594877 |
| LOC100131434 | -0.81697 | 0.528389 | -5.03408 | 0.567634 |
| LOC100270804 | -0.67253 | 0.110494 | -5.03119 | 0.627404 |
| AHSA2 | -0.71427 | 0.545225 | -4.99969 | 0.609512 |
| KCNT1 | -0.75255 | 0.140222 | -4.98866 | 0.593554 |
| BZRAP1 | -0.82123 | -0.02502 | -4.98221 | 0.56596 |
| 1-Sep | -0.94269 | 0.218755 | -4.98058 | 0.520262 |
| YJEFN3 | -0.95086 | -0.06651 | -4.9782 | 0.517325 |
| C2orf58 | -0.64441 | 0.486096 | -4.97759 | 0.639753 |
| NSUN5P1 | -0.82754 | 0.217622 | -4.97418 | 0.563491 |
| GPR162 | -0.79465 | 0.495687 | -4.97414 | 0.576482 |
| PPP1R3F | -0.64513 | -0.04433 | -4.97049 | 0.639434 |
| PHF21B | -1.16121 | 0.100781 | -4.96642 | 0.447139 |
| C14orf139 | -0.76519 | 0.10946 | -4.96678 | 0.588375 |
| C21orf29 | -1.15351 | 0.580181 | -4.96076 | 0.449531 |
| PDZD4 | -0.88911 | 0.796618 | -4.96022 | 0.539947 |
| LOC100133161 | -0.80398 | 0.432161 | -4.94844 | 0.572766 |
| PVRIG | -0.79014 | 0.365873 | -4.94874 | 0.578287 |
| SYT2 | -0.70484 | -0.45037 | -4.94858 | 0.613511 |
| SOAT2 | -0.79754 | 0.274814 | -4.94554 | 0.575327 |
| C3orf35 | -0.6809 | 0.5295 | -4.94436 | 0.623777 |
| C6orf174 | -0.94577 | 0.689085 | -4.93976 | 0.519151 |
| C1orf220 | -0.71636 | 0.611904 | -4.9394 | 0.60863 |
| STAR | -0.93488 | -0.47655 | -4.93129 | 0.523086 |
| FAM69B | -0.73349 | -0.61211 | -4.92839 | 0.601445 |
| LRDD | -0.66682 | -0.2453 | -4.92258 | 0.629896 |

| | | | | |
|---|---|---|---|---|
| USHBP1 | -0.71591 | 0.40427 | -4.90916 | 0.608819 |
| FBLL1 | -1.25276 | 0.776641 | -4.9005 | 0.419646 |
| LOC401431 | -0.85748 | -0.26895 | -4.89734 | 0.551914 |
| NRN1L | -0.81904 | -0.00554 | -4.88583 | 0.566819 |
| LOC730668 | -0.63633 | 0.250867 | -4.8855 | 0.643348 |
| LENG8 | -0.62538 | 0.642735 | -4.88366 | 0.648248 |
| LRRC39 | -0.74992 | -0.22738 | -4.87975 | 0.594637 |
| NSUN5P2 | -0.77457 | 0.207177 | -4.87338 | 0.584561 |
| LOC149134 | -0.7434 | -0.31288 | -4.85751 | 0.597331 |
| CPNE9 | -0.81548 | 0.477719 | -4.8559 | 0.568219 |
| MEF2B | -0.89972 | 0.152682 | -4.85298 | 0.53599 |
| TAL1 | -0.68805 | 0.289638 | -4.84993 | 0.620692 |
| C5orf27 | -0.74484 | -0.07 | -4.83171 | 0.596733 |
| FAM71F2 | -0.74994 | 0.325315 | -4.82995 | 0.594628 |
| PLA2G6 | -0.62369 | -0.14163 | -4.81818 | 0.649009 |
| FOXD4L1 | -0.71116 | 0.077815 | -4.81628 | 0.610829 |
| LOC219347 | -0.68013 | -1.02831 | -4.80955 | 0.624108 |
| C1orf228 | -0.81643 | 0.090574 | -4.80211 | 0.567847 |
| IGLL3 | -0.8073 | 0.656543 | -4.80236 | 0.571451 |
| CLDN5 | -0.72437 | 0.898831 | -4.79047 | 0.60526 |
| FLJ45244 | -0.65998 | 0.104677 | -4.78351 | 0.632888 |
| ZFR2 | -1.0468 | 0.155328 | -4.7781 | 0.48404 |
| FAM43B | -0.78658 | 1.57812 | -4.77152 | 0.579719 |
| FAM95B1 | -0.8629 | 0.762288 | -4.76885 | 0.549846 |
| LAT | -0.73221 | 0.381866 | -4.7505 | 0.60198 |
| GNB3 | -0.7402 | 0.787766 | -4.7441 | 0.598658 |
| JAKMIP1 | -1.12379 | 0.273021 | -4.74189 | 0.458885 |
| MAPK12 | -0.6867 | -0.25889 | -4.73879 | 0.621275 |
| MUSTN1 | -0.92455 | 0.53432 | -4.73427 | 0.526846 |
| EML6 | -0.81884 | -0.30485 | -4.73255 | 0.566898 |
| RASA4 | -0.65494 | 0.502037 | -4.72234 | 0.635101 |
| LRRC16B | -0.98183 | 0.212891 | -4.71796 | 0.506336 |
| ANKLE1 | -0.69328 | -0.76922 | -4.71754 | 0.618448 |
| LOC338758 | -0.66577 | -0.07723 | -4.71438 | 0.63035 |
| TDRD6 | -0.776 | 0.081256 | -4.71332 | 0.583985 |
| DNAH1 | -0.62774 | 0.543585 | -4.70477 | 0.647189 |
| TNFRSF25 | -0.82167 | 0.429895 | -4.69432 | 0.565788 |
| P2RX2 | -0.93279 | 0.326174 | -4.69335 | 0.523845 |
| REM2 | -0.72562 | 0.566841 | -4.68321 | 0.604737 |
| LRRC4B | -1.00962 | -0.1119 | -4.67967 | 0.496678 |
| CEACAM19 | -0.76854 | 0.734272 | -4.67315 | 0.587011 |
| DLGAP3 | -0.98519 | 0.155061 | -4.66806 | 0.50516 |
| PTH1R | -0.67248 | 0.571739 | -4.66533 | 0.627427 |
| HSD17B3 | -0.98253 | 0.230104 | -4.66173 | 0.506092 |
| LYL1 | -0.68023 | 0.412031 | -4.66215 | 0.624067 |
| RASAL3 | -0.79478 | 0.441742 | -4.63646 | 0.576429 |

| | | | | |
|---|---|---|---|---|
| ARRDC5 | -0.72905 | 0.618126 | -4.63311 | 0.603302 |
| CACNA1A | -1.20016 | 1.90498 | -4.62933 | 0.435227 |
| HSD17B14 | -0.72889 | -0.20441 | -4.6124 | 0.603366 |
| C9orf139 | -0.691 | 0.25511 | -4.60678 | 0.619424 |
| SPTBN5 | -0.94163 | 1.075522 | -4.59688 | 0.520644 |
| VWCE | -0.68963 | -1.16068 | -4.59425 | 0.620014 |
| ARHGAP4 | -0.79876 | 0.17388 | -4.59321 | 0.574842 |
| LOC100190938 | -0.71764 | -0.83486 | -4.58396 | 0.608091 |
| LRRC24 | -1.18129 | -0.39089 | -4.57712 | 0.440956 |
| LOC644165 | -0.73276 | 0.197166 | -4.56467 | 0.601752 |
| ARHGEF15 | -0.63734 | 0.544121 | -4.55603 | 0.642899 |
| GNRH1 | -0.63663 | 0.905035 | -4.54967 | 0.643212 |
| LOC100240726 | -0.72713 | 0.367596 | -4.53866 | 0.604106 |
| GPX3 | -0.95863 | -0.40464 | -4.53574 | 0.514546 |
| LYG1 | -0.66665 | -0.08442 | -4.52972 | 0.629969 |
| STAT4 | -0.80199 | 0.81231 | -4.52547 | 0.573557 |
| SPEG | -0.96548 | 0.592612 | -4.52395 | 0.512108 |
| FXYD7 | -0.836 | -0.16377 | -4.51649 | 0.560194 |
| SCRT1 | -1.13678 | 0.935742 | -4.51394 | 0.454774 |
| RAMP3 | -0.64503 | 0.7686 | -4.51181 | 0.639478 |
| RASGRP2 | -1.05891 | 0.625865 | -4.49851 | 0.479995 |
| NCRNA00173 | -0.77528 | -1.46306 | -4.49879 | 0.584277 |
| TSPAN32 | -0.82652 | 0.314082 | -4.49574 | 0.563886 |
| GDAP1L1 | -1.22031 | 0.898765 | -4.49419 | 0.429191 |
| TSPAN7 | -1.00923 | -0.23006 | -4.49132 | 0.49681 |
| SPTBN4 | -1.12094 | 0.598114 | -4.49025 | 0.459793 |
| C21orf15 | -0.89041 | -0.05557 | -4.48967 | 0.539462 |
| NCRNA00181 | -0.69677 | -0.23625 | -4.48771 | 0.616952 |
| TCAP | -0.80075 | -0.28759 | -4.4846 | 0.574049 |
| SLC12A5 | -1.13925 | 0.30603 | -4.48341 | 0.453996 |
| ATP1B2 | -0.83114 | -0.36622 | -4.48097 | 0.562085 |
| CES8 | -0.7468 | 0.115851 | -4.47835 | 0.595924 |
| FXYD1 | -0.89616 | 0.985664 | -4.47754 | 0.537315 |
| MGC16703 | -0.79763 | 0.284721 | -4.47383 | 0.575293 |
| C19orf18 | -0.69519 | 0.116759 | -4.46833 | 0.617627 |
| ICA1L | -0.65087 | -0.02746 | -4.46825 | 0.636898 |
| CXorf50B | -0.62511 | 0.562119 | -4.4641 | 0.648372 |
| MCART6 | -0.94047 | 0.644986 | -4.46329 | 0.521062 |
| RUNDC3A | -1.41069 | 1.213283 | -4.45277 | 0.376132 |
| NGFR | -1.18675 | 1.124355 | -4.45178 | 0.439292 |
| TTC23L | -0.68614 | -0.49393 | -4.44133 | 0.621516 |
| DGCR9 | -0.86573 | 0.081002 | -4.44056 | 0.54877 |
| MIR17HG | -0.69539 | 0.386633 | -4.43975 | 0.617544 |
| C2orf85 | -0.82941 | -0.11655 | -4.43818 | 0.562759 |
| SCN4A | -0.87729 | -0.79097 | -4.43598 | 0.544388 |
| STMN3 | -0.68296 | 0.259871 | -4.43395 | 0.622887 |

| | | | | |
|---|---|---|---|---|
| MIAT | -0.90999 | 0.626472 | -4.43159 | 0.532188 |
| LINGO3 | -1.03561 | 0.050626 | -4.42807 | 0.487811 |
| TSNAXIP1 | -0.63916 | 0.384096 | -4.42697 | 0.642087 |
| C19orf35 | -0.74599 | 0.538443 | -4.41685 | 0.59626 |
| PCSK1N | -1.61595 | 2.74651 | -4.41351 | 0.32625 |
| OR13A1 | -0.81646 | 0.681991 | -4.41123 | 0.567835 |
| ATOH8 | -0.72192 | -0.4024 | -4.40601 | 0.606291 |
| TREML1 | -0.75002 | 0.335073 | -4.40429 | 0.594595 |
| UPB1 | -0.78545 | -0.51218 | -4.40329 | 0.58017 |
| CRB2 | -0.81138 | -1.54987 | -4.39088 | 0.569837 |
| GOLGA2B | -0.66411 | -0.0948 | -4.38559 | 0.631077 |
| KRT72 | -0.71128 | 0.08661 | -4.38417 | 0.610777 |
| KCNK17 | -1.05414 | 2.202967 | -4.37633 | 0.481583 |
| MTMR7 | -0.76849 | 0.053233 | -4.37424 | 0.58703 |
| CPLX1 | -0.77452 | 0.327534 | -4.36158 | 0.584584 |
| SNAP91 | -1.19574 | 2.178211 | -4.36122 | 0.436563 |
| SLC22A17 | -0.84199 | 0.548745 | -4.35866 | 0.557872 |
| SLC6A13 | -0.7189 | -2.06864 | -4.35348 | 0.607562 |
| NCRNA00105 | -0.84188 | 0.441869 | -4.3524 | 0.557917 |
| FAM107A | -0.88566 | -0.07421 | -4.35157 | 0.541242 |
| 5-Sep | -0.62692 | -0.05185 | -4.35108 | 0.647559 |
| TRPM6 | -0.90265 | -0.19776 | -4.34456 | 0.534904 |
| LCN10 | -1.10551 | 0.887654 | -4.34237 | 0.464739 |
| BRSK1 | -0.71599 | 0.31733 | -4.34231 | 0.608788 |
| C5orf39 | -0.62755 | 0.322548 | -4.33062 | 0.647275 |
| MSTN | -0.69411 | -0.26222 | -4.32951 | 0.618091 |
| GCGR | -1.17913 | 1.604325 | -4.32832 | 0.441617 |
| MGC42105 | -0.83504 | 0.269354 | -4.3277 | 0.560568 |
| GIMAP1 | -0.63549 | 0.415052 | -4.32691 | 0.643721 |
| CCDC78 | -0.85853 | 0.168821 | -4.32515 | 0.551514 |
| FOXD4 | -0.74798 | -0.21322 | -4.32335 | 0.595435 |
| B3GAT1 | -1.08134 | -0.98425 | -4.3151 | 0.472588 |
| NLRP9 | -0.83107 | 0.745666 | -4.31525 | 0.562112 |
| PIPOX | -0.89552 | -0.2739 | -4.30949 | 0.537553 |
| CA3 | -0.85418 | 0.870934 | -4.30441 | 0.553181 |
| LIMD2 | -0.6952 | 0.104942 | -4.30125 | 0.617623 |
| HCG27 | -0.62676 | 0.620081 | -4.29912 | 0.647627 |
| GNG7 | -0.8002 | 0.094035 | -4.29506 | 0.574271 |
| CHST8 | -1.07422 | 0.853187 | -4.29268 | 0.474928 |
| GRRP1 | -0.76719 | 0.909192 | -4.2856 | 0.587561 |
| GRIN2C | -0.70343 | -1.13739 | -4.28441 | 0.614112 |
| SHISA7 | -1.20015 | 0.433501 | -4.27692 | 0.435229 |
| BCAM | -0.70037 | -0.50193 | -4.27348 | 0.615413 |
| C15orf59 | -0.74732 | 0.375369 | -4.27179 | 0.595709 |
| RBP5 | -0.79864 | 0.32128 | -4.2705 | 0.57489 |
| CACNA1I | -1.00581 | 0.270547 | -4.26811 | 0.49799 |

| | | | | |
|---|---|---|---|---|
| ZAP70 | -0.96591 | 0.529352 | -4.25644 | 0.511954 |
| CDH12 | -0.84475 | -0.08251 | -4.24562 | 0.556809 |
| HSF4 | -0.82418 | 0.326862 | -4.23797 | 0.564802 |
| ZNF781 | -0.67989 | 0.220049 | -4.2336 | 0.624212 |
| GIMAP5 | -0.70773 | 0.195711 | -4.2326 | 0.612281 |
| GPIHBP1 | -0.74188 | 0.020695 | -4.2204 | 0.597959 |
| DHH | -0.76782 | 0.86177 | -4.21792 | 0.587304 |
| SUSD4 | -0.91414 | 0.85555 | -4.21389 | 0.530661 |
| CNTNAP4 | -0.79623 | -0.55616 | -4.21319 | 0.575852 |
| MPZ | -0.73816 | 0.20845 | -4.21447 | 0.599503 |
| ITGA7 | -0.67354 | 0.224605 | -4.21245 | 0.626967 |
| C5orf38 | -1.25479 | -1.04851 | -4.21157 | 0.419054 |
| LGI4 | -0.72077 | 0.536774 | -4.20562 | 0.606774 |
| FAM19A1 | -0.6311 | -0.09259 | -4.20585 | 0.645682 |
| LBP | -1.55231 | 0.365659 | -4.20389 | 0.340964 |
| NCRNA00176 | -0.69173 | -0.14096 | -4.19843 | 0.619111 |
| ATP6V1G2 | -0.65358 | -0.26226 | -4.19248 | 0.635702 |
| LIPE | -0.67918 | 0.128638 | -4.19216 | 0.624519 |
| CCDC154 | -0.78843 | -0.25975 | -4.19017 | 0.578975 |
| CRIP3 | -0.90834 | 0.177319 | -4.18948 | 0.532797 |
| PTPRCAP | -0.90679 | 0.35269 | -4.18969 | 0.533371 |
| NPR1 | -0.65288 | 0.322057 | -4.18934 | 0.636009 |
| LOC283663 | -1.00294 | 1.221775 | -4.18554 | 0.498982 |
| DUSP15 | -0.75698 | -0.4047 | -4.18091 | 0.591736 |
| LTC4S | -0.81119 | 0.493599 | -4.18056 | 0.569912 |
| CYP2D6 | -0.83444 | 0.200295 | -4.17729 | 0.5608 |
| SCML4 | -0.87789 | 1.058395 | -4.17692 | 0.544164 |
| CFP | -0.8781 | 0.547308 | -4.17399 | 0.544085 |
| SERPINF2 | -0.73442 | 0.07589 | -4.1735 | 0.601058 |
| RIC3 | -1.12189 | 0.43932 | -4.16862 | 0.459493 |
| PCDHA2 | -0.74951 | 0.267103 | -4.16539 | 0.594806 |
| TRIM46 | -0.67347 | 0.682062 | -4.16353 | 0.626998 |
| MST1 | -0.63238 | -0.38229 | -4.15792 | 0.645112 |
| OLFM2 | -0.67379 | -0.23358 | -4.154 | 0.626859 |
| CERKL | -0.64199 | 0.740591 | -4.15288 | 0.64083 |
| DDX25 | -1.18896 | 0.370997 | -4.15085 | 0.43862 |
| CPT1C | -0.62457 | 1.491962 | -4.14924 | 0.648611 |
| FNDC5 | -0.68567 | -0.46767 | -4.14808 | 0.621716 |
| C4orf44 | -0.64875 | 0.278908 | -4.14326 | 0.637833 |
| CARD9 | -0.72092 | 0.264277 | -4.14102 | 0.606709 |
| CLEC3B | -0.74453 | 0.163774 | -4.12986 | 0.596862 |
| MGC16121 | -0.66759 | 0.339392 | -4.12849 | 0.629558 |
| LOC100272228 | -0.66915 | 0.33363 | -4.12486 | 0.628877 |
| CRMP1 | -0.82782 | 0.642419 | -4.12174 | 0.56338 |
| KNDC1 | -1.10325 | 0.694046 | -4.11761 | 0.465466 |
| HCST | -0.63682 | 0.282258 | -4.11536 | 0.643128 |

| | | | | |
|---|---|---|---|---|
| GDF1 | -1.00241 | -0.73937 | -4.10863 | 0.499164 |
| SCN3B | -0.95156 | 0.936643 | -4.11002 | 0.517074 |
| ITGAD | -0.85386 | -0.23482 | -4.10773 | 0.553304 |
| ANKK1 | -0.76703 | 0.198582 | -4.1041 | 0.587624 |
| DMRTC1B | -1.0823 | 1.035335 | -4.09733 | 0.472276 |
| UCHL1 | -0.76562 | 0.861261 | -4.0963 | 0.588199 |
| ZNF467 | -0.67419 | -0.00847 | -4.09648 | 0.626683 |
| SFRS13B | -0.6685 | -0.02129 | -4.09467 | 0.629159 |
| CYFIP2 | -0.64427 | -0.57844 | -4.09353 | 0.639818 |
| VWA5B2 | -1.54047 | 2.484647 | -4.09055 | 0.343772 |
| GRIA3 | -1.06622 | 1.728934 | -4.08967 | 0.477568 |
| CDK5R2 | -1.19445 | 3.025336 | -4.08505 | 0.436953 |
| KLKB1 | -1.01823 | 1.35872 | -4.0757 | 0.493721 |
| CDK5R1 | -0.70439 | 0.110757 | -4.0746 | 0.613704 |
| CORO2B | -0.68168 | 0.35776 | -4.07155 | 0.623439 |
| LRRC10B | -0.96708 | 0.862585 | -4.07102 | 0.511539 |
| NECAB2 | -1.05715 | 1.596936 | -4.06799 | 0.480582 |
| DARC | -1.30543 | 1.747735 | -4.06413 | 0.404601 |
| GPR17 | -0.70445 | 0.597861 | -4.06255 | 0.613676 |
| KLHDC8A | -0.68555 | 1.212099 | -4.06284 | 0.621769 |
| SLC4A8 | -0.8298 | 0.541517 | -4.04793 | 0.562605 |
| KIAA0087 | -0.67887 | 0.08199 | -4.04794 | 0.624655 |
| SLC25A34 | -0.73082 | 0.767919 | -4.04703 | 0.602562 |
| CETP | -0.85511 | -0.38496 | -4.04422 | 0.552824 |
| TTC16 | -0.68553 | -0.23165 | -4.04198 | 0.621779 |
| CTXN2 | -1.01079 | 0.78522 | -4.04048 | 0.496273 |
| FAM129C | -1.4456 | 0.800198 | -4.03216 | 0.367138 |
| TBC1D10C | -0.87672 | 0.402358 | -4.03061 | 0.544605 |
| PRSS50 | -1.02219 | 0.44274 | -4.02972 | 0.49237 |
| FSD1 | -0.62801 | -0.33571 | -4.02825 | 0.647068 |
| GPRASP1 | -0.7301 | 0.961135 | -4.02559 | 0.602861 |
| REEP2 | -0.88317 | 0.704867 | -4.02467 | 0.542174 |
| FTCD | -1.1962 | -0.0569 | -4.0194 | 0.436424 |
| CHRNB2 | -1.16552 | 1.005288 | -4.0148 | 0.445805 |
| PPM1E | -0.91968 | -0.32306 | -4.01239 | 0.528625 |
| UNC13A | -1.10127 | 2.169837 | -4.01154 | 0.466106 |
| LASS1 | -0.93131 | 0.102827 | -4.00963 | 0.524383 |
| AQP7P1 | -0.7949 | 1.205771 | -4.00975 | 0.576384 |
| C3orf32 | -0.85765 | 0.398051 | -4.00516 | 0.551852 |
| RTN1 | -1.03774 | 0.592154 | -4.00458 | 0.487089 |
| DNASE1L3 | -1.16775 | -0.81046 | -4.00434 | 0.445115 |
| GNG3 | -0.68008 | 0.06858 | -4.00307 | 0.62413 |
| P2RY11 | -0.68812 | -0.02152 | -4.00103 | 0.620661 |
| ADAMTSL2 | -0.6355 | 2.307517 | -3.99976 | 0.643718 |
| SLC29A4 | -1.02776 | 0.698031 | -3.9989 | 0.49047 |
| MBL1P | -0.71454 | 0.871479 | -3.99832 | 0.609401 |

| | | | | |
|---|---|---|---|---|
| MYT1 | -1.23042 | 0.215187 | -3.99697 | 0.426195 |
| MAPK10 | -0.71853 | 0.358115 | -3.99651 | 0.607716 |
| PATL2 | -0.75344 | 0.240623 | -3.994 | 0.593188 |
| NCRNA00085 | -0.6467 | 0.530638 | -3.99128 | 0.638739 |
| EBI3 | -0.78344 | 0.025842 | -3.99049 | 0.580981 |
| PCBP3 | -0.67957 | 1.50777 | -3.9868 | 0.62435 |
| FAM180B | -0.8377 | 0.35064 | -3.98302 | 0.559534 |
| GALNTL1 | -0.95783 | 0.451314 | -3.98244 | 0.51483 |
| FEV | -1.32755 | 2.92809 | -3.9818 | 0.398444 |
| SAMD11 | -0.69286 | 1.594189 | -3.98046 | 0.618625 |
| PRRT4 | -0.82941 | -1.10691 | -3.97306 | 0.562758 |
| AP3B2 | -1.11842 | 0.055142 | -3.97022 | 0.460598 |
| RHOXF1 | -0.62772 | -0.07539 | -3.9704 | 0.6472 |
| PTCHD2 | -0.96072 | 0.193125 | -3.96882 | 0.5138 |
| P2RX5 | -0.94692 | 0.193691 | -3.96887 | 0.518739 |
| NPB | -0.76747 | -0.12157 | -3.96909 | 0.587445 |
| PTCH2 | -0.69392 | 1.346743 | -3.96735 | 0.61817 |
| RGAG4 | -0.70917 | 0.248625 | -3.96669 | 0.611672 |
| STAG3 | -0.83556 | -0.01862 | -3.96638 | 0.560364 |
| ZNF80 | -0.83595 | 0.473694 | -3.96359 | 0.560215 |
| C4orf39 | -0.64295 | -0.27649 | -3.9629 | 0.640401 |
| CHD5 | -1.03639 | 1.340457 | -3.96064 | 0.487544 |
| FBXL16 | -0.9662 | -0.8788 | -3.95994 | 0.511854 |
| FAM20A | -0.68419 | 0.214196 | -3.9573 | 0.622353 |
| MYT1L | -1.15887 | 1.762561 | -3.94984 | 0.447863 |
| FIGF | -0.89931 | -0.18121 | -3.94723 | 0.536144 |
| C21orf56 | -0.66076 | -0.015 | -3.9468 | 0.632544 |
| LRRN3 | -0.84721 | -0.09761 | -3.94329 | 0.555859 |
| AGAP2 | -0.66494 | 0.191571 | -3.9407 | 0.630714 |
| HEPACAM | -0.84232 | -0.64028 | -3.93842 | 0.557745 |
| ACTL6B | -1.40345 | 1.858862 | -3.93687 | 0.378025 |
| HCN2 | -0.8497 | 0.203697 | -3.93158 | 0.5549 |
| GRIA2 | -1.30759 | 1.273157 | -3.92966 | 0.403997 |
| KEL | -0.90758 | 0.059498 | -3.92042 | 0.53308 |
| RSPO4 | -0.72955 | -1.30558 | -3.92081 | 0.603091 |
| ACAP1 | -0.82344 | 0.722587 | -3.91317 | 0.565095 |
| INSRR | -0.81095 | 0.896785 | -3.91357 | 0.570008 |
| WNT9B | -0.65368 | 0.294836 | -3.91335 | 0.635659 |
| CAPN14 | -0.68737 | -0.57078 | -3.91237 | 0.620984 |
| SEMA3G | -0.69202 | 0.430286 | -3.90447 | 0.618985 |
| PLIN1 | -1.45451 | 0.664079 | -3.9015 | 0.36488 |
| TNNT3 | -1.10369 | -0.11715 | -3.90201 | 0.465324 |
| TCEAL5 | -0.80895 | 0.74706 | -3.90086 | 0.570799 |
| JAKMIP3 | -0.64247 | 0.248033 | -3.90104 | 0.640617 |
| FAM155B | -0.79928 | -0.13618 | -3.89624 | 0.574637 |
| CLEC4F | -0.8395 | 0.592604 | -3.88486 | 0.558836 |

| | | | | |
|---|---|---|---|---|
| CALY | -1.58451 | 3.720688 | -3.88061 | 0.333437 |
| WDR17 | -0.9632 | 0.36696 | -3.8792 | 0.51292 |
| ZNF667 | -0.62426 | -0.04764 | -3.87618 | 0.648751 |
| SCN3A | -0.87048 | 0.813264 | -3.87405 | 0.546964 |
| FLJ40330 | -0.85452 | 0.415832 | -3.85919 | 0.553051 |
| EIF4E1B | -0.69963 | 0.401381 | -3.85818 | 0.615729 |
| LOC389332 | -1.21862 | 2.876495 | -3.84481 | 0.429692 |
| EFR3B | -0.70442 | -0.73482 | -3.84305 | 0.613689 |
| RGS11 | -0.74414 | 1.550178 | -3.83877 | 0.597023 |
| FAM153A | -0.89532 | 0.823559 | -3.83809 | 0.537626 |
| ZDHHC8P1 | -0.96132 | 0.780684 | -3.83719 | 0.513585 |
| TUSC5 | -1.47782 | 0.974848 | -3.83422 | 0.35903 |
| GLYAT | -0.63897 | -1.30019 | -3.83314 | 0.64217 |
| ZSCAN1 | -0.77137 | -0.26374 | -3.83236 | 0.585862 |
| MRAP | -1.03447 | 0.170963 | -3.8307 | 0.488195 |
| CRHBP | -0.76522 | -0.87765 | -3.8288 | 0.588363 |
| KIF5C | -0.9642 | 0.097912 | -3.82489 | 0.512563 |
| FAM57B | -0.85323 | -0.03027 | -3.81973 | 0.553544 |
| C14orf180 | -1.0066 | 0.488938 | -3.81771 | 0.497717 |
| LCN12 | -0.77822 | 0.339871 | -3.81608 | 0.583085 |
| PSD | -0.69228 | 2.2792 | -3.8124 | 0.618873 |
| NELL2 | -1.01513 | -1.23626 | -3.81216 | 0.494785 |
| HPCA | -0.71647 | 1.147048 | -3.8022 | 0.608584 |
| DOCK3 | -0.7305 | -0.36114 | -3.80137 | 0.602694 |
| DAZL | -0.70554 | 0.473293 | -3.79607 | 0.613214 |
| PRR22 | -0.68559 | -0.4321 | -3.78763 | 0.621751 |
| HAP1 | -0.95177 | 0.089931 | -3.78656 | 0.516998 |
| SLC7A10 | -0.89256 | -0.77405 | -3.7793 | 0.538659 |
| CELF3 | -1.50887 | 3.968216 | -3.77872 | 0.351387 |
| CAMK2B | -1.28014 | 0.442709 | -3.77765 | 0.411754 |
| SNCB | -1.04686 | 0.945735 | -3.77657 | 0.484019 |
| MATK | -0.70841 | 0.003385 | -3.77027 | 0.611993 |
| ZNF831 | -1.00671 | 0.850381 | -3.76635 | 0.49768 |
| KIF26A | -0.67517 | 0.090057 | -3.75363 | 0.626257 |
| TMEM145 | -0.98533 | 0.012913 | -3.75146 | 0.505108 |
| CYP46A1 | -0.77809 | -0.24617 | -3.74855 | 0.583137 |
| BTBD17 | -0.90639 | 0.760485 | -3.74766 | 0.533517 |
| PARVG | -0.6716 | 0.652303 | -3.74473 | 0.627809 |
| MPP2 | -0.74749 | 0.124506 | -3.74343 | 0.595639 |
| FCN3 | -0.6611 | 0.433036 | -3.74131 | 0.632394 |
| CFD | -0.79884 | 0.511946 | -3.73933 | 0.574809 |
| LOC440905 | -0.98358 | -0.12112 | -3.7369 | 0.505722 |
| SAMD3 | -0.73184 | 1.138683 | -3.73598 | 0.602134 |
| MAP4K1 | -0.85403 | 0.152233 | -3.7324 | 0.553237 |
| RIMBP2 | -1.24556 | 1.946578 | -3.72978 | 0.421744 |
| SLIT1 | -1.01989 | 0.264116 | -3.72822 | 0.493155 |

| | | | | |
|---|---|---|---|---|
| BSN | -0.85797 | 0.122539 | -3.72812 | 0.551728 |
| GRM4 | -1.29808 | 1.320519 | -3.72664 | 0.406668 |
| B4GALNT4 | -0.91437 | -0.6104 | -3.72358 | 0.530576 |
| TMPRSS6 | -1.03156 | 1.481053 | -3.72036 | 0.489182 |
| DUSP26 | -1.13015 | 2.45944 | -3.71916 | 0.456869 |
| CALB1 | -1.10772 | 0.803475 | -3.71725 | 0.464026 |
| TMEM179 | -1.09754 | 1.241365 | -3.71582 | 0.467314 |
| CD300LG | -1.05271 | -0.66658 | -3.7139 | 0.482062 |
| FUT7 | -0.63935 | 0.195272 | -3.71322 | 0.642001 |
| CABP7 | -1.20355 | 2.569013 | -3.71236 | 0.434206 |
| CADM3 | -1.11512 | 0.450176 | -3.7115 | 0.461651 |
| CMTM5 | -0.75011 | -0.2187 | -3.70345 | 0.594558 |
| BAI1 | -0.72651 | 0.081579 | -3.70329 | 0.604362 |
| S100B | -0.78332 | 0.83059 | -3.7029 | 0.581027 |
| CCL14 | -0.9833 | 0.955501 | -3.69992 | 0.50582 |
| UCN | -0.65404 | -0.15689 | -3.69911 | 0.635496 |
| RIPPLY2 | -0.88065 | 0.592074 | -3.69815 | 0.543122 |
| ACCN4 | -0.68618 | -0.09742 | -3.69723 | 0.621496 |
| BNIPL | -0.70093 | -2.18298 | -3.69694 | 0.615175 |
| ST8SIA5 | -0.65051 | -0.58762 | -3.69602 | 0.637054 |
| ARHGAP9 | -0.67262 | 0.625305 | -3.69459 | 0.627367 |
| ZDHHC22 | -0.85221 | 0.421611 | -3.69397 | 0.553936 |
| ATP1A2 | -1.0544 | -0.15616 | -3.69358 | 0.481496 |
| RASD1 | -0.87767 | 0.32797 | -3.69335 | 0.544246 |
| KCNC1 | -1.03587 | 0.483054 | -3.68427 | 0.487723 |
| BMPER | -0.8053 | -0.69697 | -3.68136 | 0.572244 |
| KCNK12 | -0.67947 | 0.541688 | -3.66887 | 0.624395 |
| TTC18 | -0.70894 | 0.139449 | -3.66707 | 0.61177 |
| F7 | -0.81729 | -0.26686 | -3.66334 | 0.567506 |
| MAST1 | -0.81356 | -0.05859 | -3.65759 | 0.568974 |
| FLJ45445 | -0.76694 | 0.922939 | -3.65769 | 0.587664 |
| AFF3 | -0.83358 | -1.18164 | -3.65317 | 0.561135 |
| GPR123 | -0.99695 | 1.549376 | -3.64809 | 0.501056 |
| LY6H | -0.87867 | 1.525509 | -3.64769 | 0.543869 |
| FCRL2 | -1.15499 | 1.101172 | -3.64733 | 0.449069 |
| NAT8L | -0.92716 | -0.53204 | -3.64663 | 0.525891 |
| PPP1R1A | -1.4378 | 1.653894 | -3.64637 | 0.369129 |
| LOC642597 | -0.96332 | 1.628409 | -3.63911 | 0.512877 |
| KCNH3 | -0.80302 | 0.277566 | -3.63652 | 0.573148 |
| PTGDS | -0.92523 | 1.520051 | -3.63285 | 0.526595 |
| MAP6 | -0.71127 | 1.269134 | -3.63167 | 0.610781 |
| RND2 | -0.6555 | -1.30996 | -3.63052 | 0.634857 |
| RYR3 | -0.62414 | -0.92106 | -3.62683 | 0.648807 |
| NR0B1 | -1.19209 | 1.90611 | -3.62486 | 0.437668 |
| LOC100302650 | -0.71259 | 0.182849 | -3.6219 | 0.610222 |
| GNG2 | -0.65534 | 0.446041 | -3.62032 | 0.634926 |

| | | | | |
|---|---|---|---|---|
| P2RX6 | -0.71871 | 0.251804 | -3.61605 | 0.607642 |
| SYT5 | -1.202 | 2.337937 | -3.6136 | 0.434674 |
| SEZ6 | -1.19859 | 1.717914 | -3.60874 | 0.4357 |
| C19orf36 | -0.62889 | 0.509204 | -3.60612 | 0.646676 |
| IQSEC3 | -0.83093 | 1.178938 | -3.60367 | 0.562165 |
| ADIPOQ | -1.95151 | 1.904391 | -3.60165 | 0.258546 |
| SYN1 | -0.67885 | 1.394256 | -3.6016 | 0.624664 |
| SYP | -1.00769 | 1.285623 | -3.60002 | 0.497341 |
| FAM159A | -0.67856 | 0.714168 | -3.60005 | 0.624787 |
| CIDEA | -1.2047 | 0.279626 | -3.59687 | 0.43386 |
| SLC6A17 | -1.13882 | 2.073935 | -3.59676 | 0.454132 |
| C3orf36 | -0.63762 | 1.673401 | -3.59595 | 0.642771 |
| C1orf127 | -1.21191 | 2.913154 | -3.59322 | 0.431696 |
| DPY19L2P4 | -0.68581 | -0.45794 | -3.58958 | 0.621656 |
| EMCN | -0.64082 | 0.175485 | -3.58722 | 0.641346 |
| BAI3 | -0.895 | 0.804925 | -3.58668 | 0.537746 |
| ASCL2 | -0.78755 | -0.41094 | -3.5813 | 0.579326 |
| TMEM63C | -1.26739 | 0.171124 | -3.57958 | 0.415412 |
| DLG2 | -0.78112 | 0.469104 | -3.5792 | 0.581915 |
| WDR86 | -0.7159 | 0.41841 | -3.57881 | 0.608826 |
| JPH4 | -0.72471 | 0.053881 | -3.57607 | 0.605119 |
| NKAIN1 | -0.74853 | -1.42902 | -3.57519 | 0.59521 |
| KCNQ2 | -1.15401 | 1.203252 | -3.5749 | 0.449375 |
| CNR1 | -0.87992 | 1.020114 | -3.57127 | 0.543398 |
| LCN6 | -0.66677 | 0.386037 | -3.56702 | 0.629917 |
| HEPN1 | -0.68998 | -0.83393 | -3.56473 | 0.619864 |
| HOXA7 | -0.72641 | -1.51271 | -3.55956 | 0.604404 |
| LRRC50 | -0.74425 | -0.3576 | -3.55553 | 0.596978 |
| SSTR3 | -1.17031 | 2.310048 | -3.55064 | 0.444327 |
| CBLN4 | -1.00142 | 1.439833 | -3.53702 | 0.499508 |
| 4-Mar | -0.98057 | 2.29217 | -3.53136 | 0.506779 |
| RGS22 | -0.72396 | 0.719158 | -3.52865 | 0.605432 |
| KCNC2 | -0.76516 | -0.0222 | -3.5261 | 0.588388 |
| F10 | -0.79974 | 2.092645 | -3.52485 | 0.574453 |
| DNASE1L2 | -0.71933 | 0.100598 | -3.5221 | 0.607379 |
| PPP1R16B | -0.63241 | 0.235959 | -3.52188 | 0.645097 |
| TMEM121 | -0.68167 | -0.05159 | -3.51821 | 0.623444 |
| CDH10 | -1.10517 | 1.318577 | -3.51254 | 0.464846 |
| DPYSL4 | -0.73405 | 0.619666 | -3.51027 | 0.601212 |
| TMC8 | -0.63275 | 0.562008 | -3.51058 | 0.644948 |
| TAGLN3 | -1.16523 | 1.90508 | -3.50883 | 0.445894 |
| KCNH6 | -1.35347 | 2.222117 | -3.50206 | 0.391349 |
| DSCAML1 | -0.97819 | 1.13255 | -3.5014 | 0.507617 |
| RUNDC3B | -0.6312 | 0.039022 | -3.50047 | 0.645641 |
| GPR113 | -0.66191 | 0.294572 | -3.49902 | 0.632043 |
| ODZ2 | -0.81111 | 0.376164 | -3.49791 | 0.569943 |

| | | | | |
|---|---|---|---|---|
| GNAO1 | -0.74949 | 1.136611 | -3.49791 | 0.594816 |
| LOC162632 | -0.90775 | 0.353429 | -3.49737 | 0.533015 |
| TBX1 | -0.78659 | -1.10925 | -3.49287 | 0.579712 |
| TTBK1 | -0.75317 | 0.399144 | -3.49041 | 0.593297 |
| DPF1 | -0.62695 | -0.91593 | -3.48621 | 0.647545 |
| PLD4 | -0.7914 | 0.535843 | -3.48551 | 0.577783 |
| MAP3K15 | -1.21149 | 1.247421 | -3.48239 | 0.431824 |
| INA | -1.10332 | 1.926569 | -3.48011 | 0.465443 |
| GCK | -1.05611 | 2.139817 | -3.47637 | 0.480926 |
| ARHGAP15 | -0.68638 | 0.525714 | -3.47539 | 0.621411 |
| STXBP5L | -1.07114 | 0.841631 | -3.47443 | 0.475943 |
| RXRG | -1.17612 | 0.624758 | -3.47268 | 0.44254 |
| SCAMP5 | -0.65468 | 0.331935 | -3.47035 | 0.635216 |
| FAM184A | -0.63034 | -0.77412 | -3.46029 | 0.646024 |
| PSTPIP1 | -0.63942 | 0.411942 | -3.45408 | 0.641972 |
| CACNA2D2 | -0.98222 | 0.793368 | -3.45215 | 0.506199 |
| P2RY8 | -0.65171 | 0.494488 | -3.44752 | 0.636526 |
| PROK1 | -0.82385 | 0.379676 | -3.447 | 0.564934 |
| SCG2 | -1.02772 | 3.915725 | -3.4467 | 0.490484 |
| C19orf30 | -1.34506 | 4.06727 | -3.43877 | 0.393638 |
| SLC6A16 | -0.68297 | 0.034623 | -3.4387 | 0.622882 |
| SVOP | -1.27794 | 3.239154 | -3.43832 | 0.412385 |
| SELP | -0.83905 | 1.806044 | -3.43588 | 0.559013 |
| JPH3 | -0.78109 | 1.470924 | -3.43137 | 0.581927 |
| LOC100188949 | -0.66349 | 0.724225 | -3.43102 | 0.63135 |
| CACNA1B | -1.09262 | 0.527383 | -3.42864 | 0.46891 |
| FMN2 | -1.05368 | 1.731681 | -3.42317 | 0.481739 |
| SEZ6L | -1.26438 | 2.756138 | -3.42137 | 0.416279 |
| NOL4 | -1.22619 | 2.049495 | -3.40829 | 0.427445 |
| GRM7 | -0.67737 | 0.551305 | -3.40436 | 0.625306 |
| ZNF385D | -0.77176 | 1.173024 | -3.40155 | 0.585701 |
| TMEM196 | -1.04321 | 2.108437 | -3.40073 | 0.485247 |
| GFRA2 | -0.66821 | -0.28759 | -3.38959 | 0.629288 |
| PAX6 | -0.82432 | 1.378371 | -3.38801 | 0.56475 |
| TMEM59L | -0.81369 | 1.726545 | -3.38679 | 0.568925 |
| CASKIN1 | -0.78797 | -1.38562 | -3.38087 | 0.57916 |
| KCNJ10 | -0.65668 | -1.61232 | -3.37893 | 0.634335 |
| GNG4 | -0.8863 | 1.085081 | -3.36864 | 0.541001 |
| UNC80 | -1.2346 | 1.901895 | -3.36441 | 0.424961 |
| C14orf115 | -0.68156 | 1.349744 | -3.36223 | 0.623493 |
| CCDC151 | -0.65647 | -0.52709 | -3.35624 | 0.634429 |
| HRNBP3 | -0.81887 | 0.364703 | -3.35506 | 0.566885 |
| PNMT | -0.87332 | 0.809132 | -3.35488 | 0.545888 |
| WNT6 | -0.63257 | -0.21869 | -3.35116 | 0.645027 |
| RMST | -0.78673 | 0.374455 | -3.3463 | 0.579656 |
| LONRF2 | -0.72101 | 0.99296 | -3.34351 | 0.606675 |

| | | | | |
|---|---|---|---|---|
| CCR7 | -0.9677 | 0.958757 | -3.3416 | 0.511321 |
| NRSN1 | -0.84081 | 1.154679 | -3.33706 | 0.55833 |
| ST18 | -1.07316 | 2.668705 | -3.33265 | 0.475278 |
| RAB39B | -0.84793 | -0.21234 | -3.33141 | 0.555581 |
| CD79B | -0.95006 | 0.670711 | -3.32646 | 0.51761 |
| HMGCLL1 | -1.01437 | 1.616494 | -3.32484 | 0.495045 |
| MCOLN2 | -0.67167 | -0.03945 | -3.31884 | 0.627782 |
| CXorf57 | -0.68036 | 0.516551 | -3.31615 | 0.624011 |
| KCNA5 | -0.93731 | 1.804409 | -3.31597 | 0.522205 |
| SCG5 | -1.03203 | 3.898225 | -3.31469 | 0.489021 |
| PPP2R2B | -0.64873 | 0.878707 | -3.31324 | 0.637843 |
| FAM153B | -0.72936 | 0.753577 | -3.31262 | 0.603169 |
| DYNC1I1 | -0.69096 | 0.737269 | -3.3113 | 0.619442 |
| C7orf52 | -1.02471 | 1.137706 | -3.30966 | 0.491508 |
| ASTN1 | -0.98593 | 0.524243 | -3.30799 | 0.5049 |
| MGAT4C | -0.75269 | 0.062258 | -3.30806 | 0.593495 |
| APLNR | -0.62495 | 1.133362 | -3.30308 | 0.648443 |
| CELF4 | -0.92532 | 0.800163 | -3.29976 | 0.526563 |
| C1QL1 | -0.97918 | 0.369036 | -3.29725 | 0.507267 |
| FLJ43390 | -1.30858 | 3.774262 | -3.29671 | 0.403718 |
| ADRA2C | -0.65401 | 0.958143 | -3.29545 | 0.635514 |
| FAM151A | -0.71519 | -0.25334 | -3.29495 | 0.609126 |
| DCX | -0.87006 | 1.156465 | -3.29349 | 0.547125 |
| KLHL14 | -0.85741 | -0.96543 | -3.29062 | 0.551944 |
| AQP7 | -0.71027 | 1.586854 | -3.28272 | 0.611205 |
| C8orf84 | -0.63755 | 0.082263 | -3.27948 | 0.642805 |
| CHODL | -0.6492 | -0.94934 | -3.27686 | 0.637633 |
| LGI3 | -0.97442 | -0.05009 | -3.27646 | 0.508946 |
| GRIK5 | -0.82182 | 0.863337 | -3.27247 | 0.565729 |
| RTN4RL1 | -0.74739 | 0.275177 | -3.26968 | 0.595682 |
| ITIH1 | -0.82895 | 1.017724 | -3.26753 | 0.562939 |
| NEFH | -0.64513 | -0.09404 | -3.26667 | 0.639436 |
| SNAP25 | -0.92657 | 2.049423 | -3.26637 | 0.526107 |
| BLK | -1.27356 | 1.335143 | -3.26551 | 0.413638 |
| NAPSB | -0.78801 | 1.055447 | -3.2641 | 0.579144 |
| CDO1 | -0.67085 | 1.253559 | -3.2614 | 0.628138 |
| SSTR2 | -0.78825 | 0.570502 | -3.23836 | 0.579047 |
| NTS | -1.18759 | 0.312304 | -3.23611 | 0.439036 |
| TF | -0.97304 | -1.11643 | -3.23612 | 0.509433 |
| GP1BA | -0.79806 | 0.139999 | -3.23449 | 0.575121 |
| MAL | -0.8154 | -2.08863 | -3.23088 | 0.56825 |
| FRMPD1 | -0.84659 | -0.16978 | -3.22936 | 0.556099 |
| PAX4 | -0.7153 | 1.336717 | -3.22556 | 0.609079 |
| KIF19 | -0.85434 | 0.960591 | -3.22317 | 0.553117 |
| OXGR1 | -0.71193 | -0.5238 | -3.22312 | 0.610504 |
| NXF2 | -0.83151 | 0.056393 | -3.22255 | 0.56194 |

| | | | | |
|---|---|---|---|---|
| VPREB3 | -1.08804 | 0.619901 | -3.21669 | 0.4704 |
| PCDHA3 | -0.63366 | 1.201311 | -3.20883 | 0.64454 |
| TNMD | -0.72767 | -0.36873 | -3.20668 | 0.603878 |
| 10-Mar | -0.66172 | 0.233778 | -3.20508 | 0.632126 |
| HIF3A | -0.62798 | 1.363314 | -3.20422 | 0.647083 |
| C5orf20 | -0.79784 | 0.028748 | -3.20047 | 0.575211 |
| CNTFR | -1.02743 | -0.98596 | -3.19925 | 0.490582 |
| RGS7 | -0.78372 | 2.054046 | -3.1986 | 0.580866 |
| PTPRN | -1.16267 | 5.564357 | -3.19698 | 0.446685 |
| NAP1L2 | -0.72228 | 0.083685 | -3.19097 | 0.606138 |
| LOC100132354 | -0.64924 | 0.716682 | -3.18627 | 0.637617 |
| DPYS | -0.65632 | -0.93508 | -3.1827 | 0.634495 |
| BCL11A | -0.69592 | -0.30373 | -3.17933 | 0.617315 |
| BTLA | -0.84691 | 0.602267 | -3.17894 | 0.555974 |
| MAPK8IP2 | -0.76787 | 0.039103 | -3.17861 | 0.587284 |
| GNAZ | -0.63779 | 0.377684 | -3.17171 | 0.642698 |
| KCNJ11 | -0.77697 | 0.783974 | -3.16366 | 0.58359 |
| PTPRQ | -0.63395 | -0.1657 | -3.16098 | 0.644409 |
| CD37 | -0.67222 | 0.830292 | -3.15973 | 0.627542 |
| IGSF11 | -0.68356 | 0.860837 | -3.15916 | 0.622626 |
| C1orf168 | -0.90753 | 0.200846 | -3.15845 | 0.533095 |
| IRX2 | -1.10603 | -0.62181 | -3.15656 | 0.464571 |
| ARX | -1.14241 | 2.352593 | -3.15606 | 0.453002 |
| ATCAY | -0.9512 | 1.616401 | -3.1557 | 0.517204 |
| VWDE | -0.99518 | 1.441825 | -3.15463 | 0.501672 |
| APOE | -0.69129 | -0.32451 | -3.15455 | 0.619299 |
| PRR18 | -0.66328 | 0.105363 | -3.15429 | 0.631441 |
| TCL1A | -1.4211 | 1.006958 | -3.14868 | 0.373427 |
| RSPH4A | -0.64197 | -0.28538 | -3.14847 | 0.640838 |
| PLCXD3 | -0.89439 | 2.735429 | -3.14589 | 0.537973 |
| HHATL | -0.86997 | -0.45983 | -3.14555 | 0.547158 |
| RFX6 | -1.21026 | 3.904981 | -3.1314 | 0.432192 |
| FFAR1 | -1.0082 | 2.656742 | -3.13144 | 0.497165 |
| BEX2 | -0.74704 | -0.14904 | -3.12768 | 0.595825 |
| MAPT | -0.75697 | -1.16182 | -3.12487 | 0.591737 |
| CD22 | -0.92203 | 0.595437 | -3.12399 | 0.527767 |
| LHFPL4 | -1.08466 | 2.574168 | -3.1234 | 0.471503 |
| DSCAM | -0.93975 | 0.937318 | -3.12329 | 0.521323 |
| CACNA1G | -0.65082 | -0.06238 | -3.11717 | 0.636917 |
| TCEAL2 | -0.98322 | 1.11108 | -3.11536 | 0.505849 |
| TEKT2 | -0.76276 | 0.364353 | -3.11295 | 0.589369 |
| ABCC8 | -1.457 | 5.312901 | -3.11216 | 0.364249 |
| INSM1 | -1.15054 | 3.125834 | -3.10781 | 0.450458 |
| NPM2 | -0.70671 | 0.830461 | -3.10687 | 0.612715 |
| ODZ1 | -0.82616 | -1.41658 | -3.10674 | 0.56403 |
| GIPR | -0.71826 | 3.301002 | -3.10338 | 0.60783 |

| | | | | |
|---|---|---|---|---|
| KCNB2 | -0.81105 | 1.806757 | -3.09999 | 0.569967 |
| NFASC | -0.64123 | 0.828458 | -3.09783 | 0.641166 |
| KLRK1 | -0.65515 | 0.240423 | -3.09185 | 0.635011 |
| OLFM1 | -0.65801 | 0.450373 | -3.09164 | 0.633753 |
| VGF | -1.06795 | 2.933798 | -3.09139 | 0.476995 |
| EFNB3 | -0.64534 | 0.409503 | -3.08606 | 0.639344 |
| TRAF3IP3 | -0.6417 | 0.48156 | -3.08582 | 0.640956 |
| NTRK3 | -0.62536 | -0.32879 | -3.08518 | 0.648256 |
| FCER2 | -1.26925 | 1.074202 | -3.0842 | 0.414875 |
| BEX1 | -1.17632 | 2.473284 | -3.07933 | 0.44248 |
| LGI1 | -0.71184 | 0.617874 | -3.07081 | 0.610542 |
| ANK2 | -0.69794 | 0.246071 | -3.06594 | 0.61645 |
| ASPA | -0.62486 | 0.335586 | -3.06186 | 0.648481 |
| SLC16A11 | -0.65787 | 0.395856 | -3.05883 | 0.633812 |
| ADCYAP1 | -1.02119 | 3.52895 | -3.0521 | 0.49271 |
| SBK2 | -0.65439 | 0.791984 | -3.05196 | 0.635345 |
| PON1 | -0.84179 | 0.529419 | -3.0507 | 0.557951 |
| ACSL6 | -0.73234 | -0.22147 | -3.05078 | 0.601925 |
| RGS9 | -0.71397 | 1.503384 | -3.04639 | 0.609641 |
| RERGL | -0.79585 | 0.486351 | -3.03755 | 0.576002 |
| ANKRD55 | -0.6394 | 0.322672 | -3.03676 | 0.64198 |
| KIAA1409 | -0.98644 | 1.276231 | -3.03383 | 0.504721 |
| ATP1A3 | -0.86202 | 0.589288 | -3.02594 | 0.550183 |
| PCDHA1 | -0.83247 | 0.240081 | -3.02495 | 0.561566 |
| TACR1 | -0.75728 | 0.29227 | -3.02265 | 0.591611 |
| KCNH2 | -0.74881 | 1.935123 | -3.02045 | 0.595093 |
| DRD2 | -0.69175 | 0.548632 | -3.01626 | 0.619103 |
| CCL19 | -1.26327 | 2.324693 | -3.01294 | 0.416598 |
| KL | -0.76808 | 0.246189 | -3.01297 | 0.5872 |
| C1orf175 | -0.6499 | 1.25716 | -3.00999 | 0.637324 |
| TNNT2 | -0.88459 | 1.148688 | -3.00832 | 0.541642 |
| UGT2B4 | -0.83824 | -0.1979 | -3.00623 | 0.559326 |
| PPP4R4 | -0.82566 | -0.35669 | -3.00247 | 0.564224 |
| PLIN4 | -1.08001 | 0.678995 | -2.99728 | 0.473024 |
| POMC | -0.6506 | 0.321389 | -2.99223 | 0.637017 |
| TLR10 | -0.80564 | 0.501907 | -2.99145 | 0.572108 |
| SPAG6 | -0.89747 | -0.18756 | -2.98687 | 0.536826 |
| PRG4 | -0.72825 | 2.899617 | -2.98567 | 0.603637 |
| LOC283174 | -0.64765 | 1.4508 | -2.98267 | 0.638321 |
| PCP4 | -0.9333 | 0.705458 | -2.98094 | 0.523659 |
| TMEM132C | -0.80697 | 2.296961 | -2.98023 | 0.571579 |
| CRYBA2 | -1.34051 | 4.276082 | -2.97904 | 0.39488 |
| LINGO4 | -0.64226 | 0.190519 | -2.97827 | 0.640711 |
| MAPK4 | -0.82438 | 0.126555 | -2.97522 | 0.564724 |
| CBLN1 | -0.80726 | 0.904684 | -2.973 | 0.571466 |
| CCNA1 | -0.62538 | -0.77056 | -2.97068 | 0.648247 |

| | | | | |
|---|---|---|---|---|
| ZNHIT2 | -0.63203 | 0.512264 | -2.97 | 0.645266 |
| GZMM | -0.65084 | 0.222172 | -2.96449 | 0.636907 |
| MSI1 | -0.63421 | 0.333308 | -2.9609 | 0.644296 |
| C4orf7 | -1.44018 | 2.808245 | -2.95955 | 0.368522 |
| SLCO1A2 | -0.67713 | -1.30425 | -2.948 | 0.625407 |
| C12orf34 | -0.64776 | -0.29322 | -2.94638 | 0.638272 |
| GPR142 | -0.81671 | 2.175908 | -2.94526 | 0.567733 |
| FCRL1 | -1.10419 | 1.017199 | -2.94505 | 0.465163 |
| FAM71E2 | -0.72976 | 0.763751 | -2.94222 | 0.603005 |
| AGT | -0.72061 | 1.775999 | -2.9421 | 0.606841 |
| AMH | -0.87471 | 0.296914 | -2.93591 | 0.545365 |
| APLP1 | -0.84606 | 2.225724 | -2.93164 | 0.556302 |
| GJD2 | -1.11651 | 3.459355 | -2.92104 | 0.461207 |
| KCNMB2 | -1.03263 | 2.311613 | -2.91989 | 0.488819 |
| SLC35F4 | -0.63896 | 0.810212 | -2.91898 | 0.642174 |
| IGFN1 | -0.83299 | 1.066397 | -2.91616 | 0.561364 |
| NKX2-2 | -1.20768 | 4.342805 | -2.91477 | 0.432965 |
| DCLK1 | -0.64215 | 0.015486 | -2.91221 | 0.640758 |
| THRSP | -0.83338 | -1.1583 | -2.90114 | 0.561214 |
| TNFRSF13B | -0.88519 | 0.681281 | -2.90061 | 0.541417 |
| DPYSL5 | -0.70792 | -0.17252 | -2.89804 | 0.612201 |
| OGDHL | -0.95472 | -0.27662 | -2.89792 | 0.515941 |
| HLA-DOB | -0.69729 | 0.721378 | -2.89556 | 0.616728 |
| TNFRSF13C | -0.76568 | 0.370002 | -2.89491 | 0.588174 |
| ADH1B | -1.06868 | 2.829442 | -2.88747 | 0.476756 |
| NCF1 | -0.63622 | 0.450022 | -2.88375 | 0.643396 |
| NRXN1 | -0.88293 | 2.565223 | -2.87756 | 0.542265 |
| SERPINA10 | -0.97867 | 1.818929 | -2.87454 | 0.507446 |
| FAIM3 | -0.70457 | 0.560106 | -2.87416 | 0.613627 |
| SLITRK2 | -0.63667 | -0.11089 | -2.86539 | 0.643197 |
| DPY19L2P1 | -0.63682 | -0.98608 | -2.86379 | 0.643128 |
| LOC389493 | -0.75981 | 0.521497 | -2.85958 | 0.590576 |
| C1orf158 | -0.65707 | 0.283045 | -2.8563 | 0.634166 |
| SYCE1L | -0.74196 | 0.284353 | -2.85559 | 0.597928 |
| PLA2G2D | -0.9652 | 0.430357 | -2.85152 | 0.512206 |
| MCOLN3 | -0.69665 | 0.605217 | -2.84746 | 0.617003 |
| IRX1 | -0.84279 | -1.03102 | -2.84344 | 0.557564 |
| CHGA | -1.32038 | 6.586079 | -2.83623 | 0.400429 |
| LOC284749 | -0.76547 | 0.931309 | -2.83532 | 0.588262 |
| CLU | -0.63788 | 0.311312 | -2.83405 | 0.642657 |
| GLRA3 | -0.64565 | 0.51994 | -2.83254 | 0.639206 |
| FAM5B | -0.63659 | -0.79751 | -2.82791 | 0.643231 |
| C5orf49 | -0.64156 | 0.079702 | -2.82289 | 0.641018 |
| GPR18 | -0.65962 | 0.385659 | -2.82072 | 0.633043 |
| PLCH2 | -0.65612 | 0.949605 | -2.81421 | 0.634581 |
| PACSIN1 | -0.66398 | -0.23913 | -2.80764 | 0.631133 |

| | | | | |
|---|---|---|---|---|
| FAM123C | -0.95051 | 3.03474 | -2.80717 | 0.51745 |
| LECT1 | -0.72106 | 0.679969 | -2.80562 | 0.606651 |
| TPO | -0.70345 | 0.512954 | -2.80226 | 0.614104 |
| PTPN5 | -0.71539 | 1.476811 | -2.79806 | 0.609041 |
| KIAA0748 | -0.6526 | 0.536905 | -2.79749 | 0.636131 |
| DKFZp434J0226 | -0.72056 | 2.305441 | -2.79692 | 0.60686 |
| INE2 | -0.75527 | -0.74428 | -2.79353 | 0.592435 |
| CTNNA2 | -0.75369 | 0.941649 | -2.7925 | 0.593087 |
| NCF1B | -0.62545 | 0.421916 | -2.79249 | 0.648218 |
| PI16 | -1.09437 | 1.492588 | -2.79199 | 0.468342 |
| NEFM | -0.64498 | 1.229669 | -2.79119 | 0.6395 |
| FABP4 | -1.02304 | 1.08765 | -2.78676 | 0.492079 |
| IGSF1 | -0.81807 | 0.566907 | -2.78539 | 0.5672 |
| SLC35F3 | -0.73465 | 1.051698 | -2.78212 | 0.600963 |
| RASGRF1 | -0.84423 | 1.06134 | -2.78077 | 0.557008 |
| KIAA0125 | -0.9429 | 0.59634 | -2.77846 | 0.520186 |
| ACTC1 | -0.8441 | 0.467959 | -2.77209 | 0.557059 |
| CD19 | -1.14627 | 0.855191 | -2.76279 | 0.451791 |
| C1orf173 | -0.73707 | -0.06372 | -2.761 | 0.599958 |
| CNTN2 | -0.69076 | 0.277268 | -2.76027 | 0.619528 |
| HMP19 | -0.84349 | 2.393335 | -2.7591 | 0.557296 |
| KCNB1 | -0.72439 | -0.01609 | -2.75636 | 0.605253 |
| C16orf89 | -0.81016 | 0.687134 | -2.7501 | 0.570319 |
| C4orf48 | -0.87032 | 0.695726 | -2.74712 | 0.547026 |
| CXCR5 | -0.90502 | 1.307041 | -2.74658 | 0.534026 |
| TRPM5 | -0.85902 | 3.018761 | -2.74595 | 0.551329 |
| DACH2 | -0.72017 | 0.912305 | -2.7452 | 0.607028 |
| GABRG2 | -0.8296 | 1.095069 | -2.74508 | 0.562686 |
| L1CAM | -0.7859 | 1.303095 | -2.74466 | 0.579989 |
| KCNJ6 | -0.68084 | 1.654029 | -2.74392 | 0.6238 |
| SULT4A1 | -0.77569 | 1.003118 | -2.7363 | 0.584108 |
| KCNH8 | -0.6371 | 2.447243 | -2.73602 | 0.643004 |
| SORCS1 | -0.74445 | 0.99102 | -2.73179 | 0.596897 |
| CYP2C8 | -0.7395 | 1.578518 | -2.72733 | 0.598945 |
| LEP | -1.04959 | 1.159216 | -2.72265 | 0.483105 |
| DPP6 | -0.97165 | 2.332919 | -2.71992 | 0.509922 |
| SCG3 | -1.15006 | 4.193168 | -2.71927 | 0.450605 |
| LGALS12 | -0.66432 | 0.512815 | -2.71353 | 0.630984 |
| CD27 | -0.64064 | 0.399461 | -2.69875 | 0.641428 |
| CLGN | -0.81884 | -0.53522 | -2.69627 | 0.566897 |
| RELN | -0.91048 | 1.355233 | -2.6932 | 0.532007 |
| LOC145837 | -0.7407 | -0.4532 | -2.68833 | 0.598449 |
| GRIK3 | -0.63218 | 0.67372 | -2.6884 | 0.645202 |
| ADH1A | -0.77287 | 1.800092 | -2.68739 | 0.585253 |
| SH3GL2 | -0.95755 | 1.20174 | -2.68721 | 0.514929 |
| RAB3C | -0.66048 | 2.114815 | -2.68384 | 0.632667 |

| | | | | |
|---|---|---|---|---|
| SYT7 | -0.70041 | 0.803595 | -2.68272 | 0.615396 |
| CD48 | -0.63068 | 0.520297 | -2.68224 | 0.645872 |
| PCSK1 | -0.98987 | 3.494406 | -2.67534 | 0.503524 |
| CHRNA1 | -0.66204 | 0.569235 | -2.67281 | 0.631982 |
| CLEC17A | -0.79735 | 0.705178 | -2.67118 | 0.575406 |
| FCRLA | -1.00523 | 0.122642 | -2.66742 | 0.498191 |
| LRFN2 | -0.64633 | 0.418411 | -2.65783 | 0.638902 |
| KCNIP1 | -0.7282 | 1.326268 | -2.65572 | 0.603657 |
| CAPSL | -0.76261 | -0.14856 | -2.65404 | 0.589428 |
| CPLX2 | -1.1865 | 4.296433 | -2.65355 | 0.439366 |
| NCF1C | -0.63328 | 0.426044 | -2.64664 | 0.644708 |
| NCAM1 | -0.72208 | 0.674593 | -2.64324 | 0.606223 |
| CA10 | -0.69998 | 0.053437 | -2.6359 | 0.615582 |
| GPD1 | -0.73396 | 1.69957 | -2.63114 | 0.60125 |
| C19orf20 | -0.69438 | -0.02664 | -2.6307 | 0.617976 |
| CD1E | -0.70701 | 1.332414 | -2.62927 | 0.612589 |
| AVPR1B | -0.62925 | 1.125793 | -2.628 | 0.646513 |
| SYT4 | -1.03887 | 4.113204 | -2.62289 | 0.486708 |
| MS4A1 | -1.26213 | 1.167415 | -2.62181 | 0.416928 |
| LMO1 | -0.76586 | 1.034764 | -2.61904 | 0.588101 |
| VTN | -0.91932 | 3.113579 | -2.61627 | 0.528759 |
| CHGB | -1.18927 | 5.573051 | -2.61594 | 0.438524 |
| TTLL6 | -0.6829 | 2.410403 | -2.61059 | 0.622914 |
| RIMS2 | -0.78702 | -0.01819 | -2.61007 | 0.579539 |
| PART1 | -0.81991 | -0.62456 | -2.60988 | 0.566478 |
| NEURL | -0.78681 | 2.427322 | -2.59896 | 0.579623 |
| LOC126536 | -0.76818 | 2.208686 | -2.59394 | 0.587157 |
| FCRL3 | -0.8359 | 0.905579 | -2.59262 | 0.560233 |
| XKR7 | -0.64991 | 0.879005 | -2.59041 | 0.63732 |
| ABCA8 | -0.70834 | 1.386693 | -2.57782 | 0.612024 |
| PCDH8 | -0.67807 | 0.165265 | -2.57698 | 0.624999 |
| CNR2 | -0.79803 | 0.740531 | -2.57297 | 0.575134 |
| ANKS1B | -0.65387 | -0.91875 | -2.57239 | 0.635574 |
| C1orf230 | -0.67461 | 1.080578 | -2.57058 | 0.626501 |
| GPR44 | -0.65165 | 1.517203 | -2.56479 | 0.636553 |
| SCARA5 | -1.021 | 1.174504 | -2.56318 | 0.492774 |
| SLC7A14 | -0.92659 | 2.970634 | -2.55754 | 0.526099 |
| APOH | -1.02869 | 3.702684 | -2.54866 | 0.490156 |
| GZMK | -0.69119 | 0.88265 | -2.54452 | 0.619343 |
| VIT | -0.73759 | 0.626403 | -2.54304 | 0.599738 |
| TMEM132D | -0.89924 | 2.102583 | -2.54228 | 0.53617 |
| WNK4 | -0.77715 | -0.04496 | -2.53951 | 0.583517 |
| HCN4 | -0.6314 | 1.008653 | -2.53964 | 0.645548 |
| UPK3BL | -0.72532 | -0.19906 | -2.53672 | 0.604862 |
| SCRT2 | -0.62578 | 0.891467 | -2.53123 | 0.648067 |
| SFRP1 | -0.78914 | 0.530132 | -2.52412 | 0.57869 |

| | | | | |
|---|---|---|---|---|
| CLEC18B | -0.62472 | -0.15206 | -2.52085 | 0.648544 |
| SLC6A4 | -0.80339 | 1.265223 | -2.51964 | 0.573003 |
| COL4A3 | -0.68944 | 0.067251 | -2.51408 | 0.620096 |
| CFHR1 | -0.63562 | 0.263842 | -2.51041 | 0.643666 |
| MIOX | -0.67422 | 0.588339 | -2.49384 | 0.626671 |
| KCNA6 | -0.65102 | 1.779859 | -2.49022 | 0.636829 |
| ASCL1 | -0.66229 | -0.21104 | -2.48443 | 0.631877 |
| C7 | -0.74808 | 4.273564 | -2.47837 | 0.595396 |
| WISP2 | -0.66293 | 0.626469 | -2.45941 | 0.631595 |
| ATP2B2 | -0.62833 | -0.87281 | -2.45918 | 0.646925 |
| CXCL13 | -1.07633 | 0.502745 | -2.45522 | 0.474235 |
| ECEL1 | -0.64199 | 1.897521 | -2.43604 | 0.640829 |
| CD1C | -0.68726 | 1.813457 | -2.43158 | 0.621033 |
| ZBTB16 | -0.70163 | 1.28588 | -2.42452 | 0.614877 |
| FAM135B | -0.77482 | 1.129467 | -2.42224 | 0.58446 |
| FAM163A | -0.64515 | 1.539612 | -2.41067 | 0.639428 |
| CDKN2A | -0.81619 | -0.19708 | -2.3983 | 0.567939 |
| SLITRK1 | -0.63389 | 0.794453 | -2.38457 | 0.644437 |
| MLXIPL | -0.64035 | 2.262297 | -2.36857 | 0.641557 |
| LY6K | -0.87127 | -1.0687 | -2.36586 | 0.546665 |
| RALYL | -0.71704 | 2.300006 | -2.3407 | 0.608345 |
| SLC4A10 | -0.77348 | 2.129407 | -2.33691 | 0.585006 |
| ERO1LB | -0.63506 | 1.144287 | -2.33608 | 0.643916 |
| UCN3 | -0.86495 | 4.31011 | -2.33526 | 0.549064 |
| ELAVL4 | -0.66863 | 1.549603 | -2.33493 | 0.629105 |
| MAFA | -0.6714 | 1.618309 | -2.32891 | 0.627899 |
| ARC | -0.63725 | -0.50852 | -2.32871 | 0.642937 |
| LILRA4 | -0.63976 | 0.316548 | -2.32447 | 0.641819 |
| PRPH | -0.76049 | 2.195416 | -2.316 | 0.590294 |
| CFC1B | -1.18122 | 5.859296 | -2.30789 | 0.440978 |
| KIF5A | -0.62553 | 1.391429 | -2.28927 | 0.648181 |
| LPPR1 | -0.62974 | -0.38031 | -2.28291 | 0.646295 |
| PRKCG | -0.75154 | 2.480952 | -2.28139 | 0.593967 |
| RTBDN | -0.7369 | 1.306767 | -2.27866 | 0.600028 |
| GHRL | -0.75545 | 2.360762 | -2.26079 | 0.592361 |
| PCSK2 | -1.08665 | 4.612013 | -2.25834 | 0.470854 |
| SPOCK3 | -0.73036 | 0.661189 | -2.23498 | 0.602753 |
| KCNK16 | -1.05173 | 5.388461 | -2.23332 | 0.482388 |
| NEUROD1 | -0.94591 | 5.232371 | -2.22268 | 0.519103 |
| JSRP1 | -0.64714 | 1.658097 | -2.21957 | 0.638543 |
| DAPL1 | -0.62589 | 0.175564 | -2.21364 | 0.64802 |
| CR2 | -1.27628 | 2.142257 | -2.20739 | 0.41286 |
| BIRC7 | -0.69164 | 1.784894 | -2.20526 | 0.619149 |
| SST | -1.01146 | 7.501049 | -2.19573 | 0.496044 |
| ATRNL1 | -0.64706 | 0.162978 | -2.1896 | 0.638582 |
| PAX5 | -0.77393 | 0.735405 | -2.18246 | 0.584821 |

| | | | | |
|---|---|---|---|---|
| GPR119 | -0.7129 | 2.43677 | -2.1754 | 0.610091 |
| CHIT1 | -0.87302 | 1.401594 | -2.1696 | 0.546003 |
| SYNGR4 | -0.64541 | 1.547629 | -2.15676 | 0.639311 |
| GPR98 | -0.67618 | -1.2883 | -2.15621 | 0.62582 |
| C13orf36 | -0.67288 | 1.19131 | -2.15546 | 0.627254 |
| PTPRT | -0.62505 | 0.602247 | -2.14414 | 0.648398 |
| CHRDL1 | -0.73573 | 1.483152 | -2.12877 | 0.600516 |
| PTX3 | -0.6497 | 1.207115 | -2.08262 | 0.637415 |
| HS6ST3 | -0.67689 | 0.753618 | -2.0812 | 0.625513 |
| CCL21 | -0.75335 | 2.613068 | -2.07 | 0.593226 |
| CLCA2 | -0.65922 | -1.55667 | -1.99446 | 0.63322 |
| IGF1 | -0.6625 | 0.741902 | -1.98512 | 0.631781 |
| CCL17 | -0.68536 | 2.244204 | -1.97826 | 0.62185 |
| ANGPTL7 | -0.75561 | 1.975744 | -1.97813 | 0.592295 |
| HP | -0.65415 | 0.84541 | -1.96303 | 0.635451 |
| MADCAM1 | -0.65363 | 3.25194 | -1.95367 | 0.635677 |
| HBA1 | -0.94451 | -1.17786 | -1.93902 | 0.519606 |
| FSTL5 | -0.63093 | 1.505637 | -1.93741 | 0.645761 |
| CD79A | -0.7146 | 1.272561 | -1.93567 | 0.609373 |
| XPNPEP2 | -0.6368 | 0.111048 | -1.93459 | 0.643136 |
| SAA1 | -0.62978 | 2.361972 | -1.8966 | 0.646276 |
| KIF1A | -0.70226 | 2.646808 | -1.89482 | 0.614609 |
| DDC | -0.66973 | 3.053047 | -1.89184 | 0.628625 |
| RPS28 | -0.79814 | 0.291033 | -1.89054 | 0.575091 |
| GAD2 | -0.80037 | 4.443524 | -1.87475 | 0.574204 |
| CRH | -0.71097 | 2.141334 | -1.8476 | 0.61091 |
| G6PC2 | -0.94707 | 5.675587 | -1.83817 | 0.518685 |
| SCGN | -0.83783 | 5.332842 | -1.82322 | 0.559486 |
| CDH22 | -0.67731 | 2.596132 | -1.80827 | 0.625332 |
| NPHS1 | -0.6697 | 2.749053 | -1.80267 | 0.628637 |
| CYP2C9 | -0.63473 | 3.982243 | -1.7717 | 0.644063 |
| DLK1 | -0.85395 | 4.07284 | -1.76121 | 0.553267 |
| IAPP | -0.95265 | 5.477357 | -1.71189 | 0.516681 |
| HEPACAM2 | -0.64299 | 3.81685 | -1.64168 | 0.640385 |
| TSIX | -0.8441 | -0.19474 | -1.46824 | 0.557058 |
| TTR | -0.83465 | 8.892266 | -1.37051 | 0.56072 |
| XIST | -0.71933 | -0.37398 | -0.85427 | 0.607378 |

**Table S4.** Association between 5-lncRNA signature-based classification and clinic-pathologic characteristics in PDAC patients involved in the study.

| Features | Low-risk group | High-risk group | *P* value |
|---|---|---|---|
| Entire TCGA cohort (N=177) | N=89 | N=88 | |
| Age at diagnosis, years | | | |
| ≤60 | 29(32.58%) | 25(28.41%) | 0.546 |
| >60 | 60(67.42%) | 63(71.59%) | |
| Gender | | | |
| Female | 40(44.94%) | 40(45.45%) | 0.944 |
| Male | 49(55.06%) | 48(54.55%) | |
| MSI status | | | |
| MSI-I | 17(19.10%) | 11(12.5%) | 0.131 |
| MSI-L | 2(2.25%) | 7(7.95%) | |
| MSS | 70(78.65%) | 70(79.55%) | |
| Histologic grade | | | |
| G1 | 17(19.32%) | 13(14.94%) | 0.547 |
| G2 | 50(56.82%) | 45(51.72%) | |
| G3 | 20(22.73%) | 28(32.18%) | |
| G4 | 1(1.14%) | 1(1.15%) | |
| TNM stage | | | |
| I | 17(19.54%) | 4(4.55%) | **0.024** |
| II | 66(75.86%) | 79(89.77%) | |
| III | 2(2.30%) | 2(2.27%) | |
| IV | 2(2.30%) | 3(3.41%) | |
| OS status | | | |
| Alive | 59(66.29%) | 26(29.55%) | **<0.001** |
| Dead | 30(33.71%) | 62(70.45%) | |
| Fudan Validation cohort (N=46) | N=23 | N=23 | |
| Age at diagnosis, years | | | |
| ≤60 | 12(52.17%) | 11(47.83%) | 0.768 |
| >60 | 11(47.83%) | 12(52.17%) | |
| Gender | | | |
| Female | 10(43.48%) | 11(47.83%) | 0.767 |
| Male | 13(56.52%) | 12(52.17%) | |
| TNM stage | | | |
| I | 6(27.27%) | 4(19.05%) | 0.450 |
| II | 12(54.55%) | 9(42.86%) | |
| III | 4(18.18%) | 7(33.33%) | |
| IV | — | 1(4.76%) | |
| OS status | | | |
| Alive | 16(69.57%) | 7(30.43%) | **0.008** |
| Dead | 7(30.43%) | 16(69.57%) | |

Microsatellite instability (MSI); Microsatellite stable (MSS); MSI-indeterminate (MSI-I); MSI-low

(MSI-L); TNM, Tumor node metastasis
